# Population Genomics Reveals Genetic Diversity, Introgression, and Genetic Differentiation in Tianshan Mountains Western Honeybees ( *Apis mellifera* )

**DOI:** 10.1111/eva.70248

**Published:** 2026-05-06

**Authors:** Gulinuer Tulaxi, Baheitiya Abaidula, Fengfeng Shi, Naisibai Parihati, Yilidana Dilixiati, Caixue Yang, Ming Li, Arong Luo, Jiwei Qi, Chaodong Zhu, Hongying Hu

**Affiliations:** ^1^ College of Life Science and Technology Xinjiang University Urumqi Xinjiang P. R. China; ^2^ Yili Kazakh Autonomous Prefecture Institute of Agricultural Sciences Yili Xinjiang P. R. China; ^3^ Xinjiang Key Laboratory of Biological Resources and Genetic Engineering Urumqi Xinjiang P. R. China; ^4^ Nileke County Animal Husbandry and Veterinary Development Center Yili Xinjiang P. R. China; ^5^ Institute of Zoology of CAS Beijing P. R. China

**Keywords:** *Apis mellifera*, conservation strategies, introgression, Tianshan Mountains, Xinjiang black honeybee

## Abstract

The Tianshan Mountains, which host two native subspecies of western honeybees, represent the easternmost natural distribution limit of *Apis mellifera* . The managed Xinjiang black honeybee (*XJ*), introduced a century ago and designated as a Chinese National Animal Genetic Resource, has expanded rapidly under anthropogenic management. However, this expansion simultaneously threatens populations of native subspecies *Apis mellifera sinisxinyuan* and *Apis mellifera pomonella*. Herein, we performed the first whole‐genome resequencing of the *XJ* population and analyzed whole‐genome data from 19 *XJ* workers and 172 global *A. mellifera* samples to clarify the evolutionary history of *XJ* and evaluate its interactions with native bees. Population structure and phylogenomic analyses showed that *XJ* clustered within C lineage but formed a divergent clade distinct from other C lineage subspecies, with the closest affinity to *Apis mellifera carnica* (FST = 0.053). Despite higher inbreeding than other C lineage subspecies, *XJ* displayed comparatively higher genetic diversity (Θπ = 2.15 × 10^−3^) and heterozygosity (0.0028) than other C lineage populations (0.0015), although *XJ*'s value falls within the global range of *A. mellifera* . This genetic pattern can be attributed to substantial introgression (~10%–15%) from the M lineage, specifically from the native *A. m. sinisxinyuan*. TreeMix and F‐branch analysis identified significant gene flow from *A. m. sinisxinyuan* into the ancestral population of the *XJ*. Functional enrichment analysis suggested that genes within introgressed regions are involved in cold adaptation and foraging efficiency, and independent transcriptome validation confirmed differential expression of key candidate genes (e.g., LOC552291/MCM4) within these introgressed regions. Overall, our findings indicate that *XJ* represents an introduced population that has undergone regional adaptation, facilitated by the introgression of potentially adaptive alleles from native taxa. This case underscores the need for conservation strategies balancing management of economically valuable populations with protection of native lineages from genetic swamping and ecological competition.

## 1 Introduction

Human activities are rapidly altering the planet and impacting both long‐term biological evolution and global biodiversity (Tollefson 2019). These processes leave imprints on the genomes of many species that are hybrid, managed, or transported across biogeographic regions (Graf et al. 2021). The ongoing decline in insect pollinators threatens both human welfare and agriculture, prompting widespread introduction of managed colonies of *A. mellifera* to meet the demand for crop pollination (Klein et al. 2018; Potts et al. 2016; Rafferty 2017). *A. mellifera* is renowned not only as a honey producer but also as a keystone agricultural pollinator for numerous crops and plants (Garibaldi et al. 2017). However, modern beekeeping practices, including migratory hive movement, standardized queen breeding, and transregional trade, have also accelerated artificial introgression of nonlocal subspecies, thereby reshaping the genetic structure of local populations (Sills et al. 2018; Yusuf et al. 2015). Hybridization and subsequent introgression represent a double‐edged sword in conservation biology (Schneider and Cannon 2025). On one hand, intentional gene flow can restore genetic diversity and rescue small, inbred populations from extinction (Baskett and Gomulkiewicz 2011). On the other hand, uncontrolled introgression from more abundant congeners or domesticated relatives can threaten the genetic integrity of rare species through genetic swamping, the loss of locally adapted alleles, or outbreeding depression (Howard‐McCombe et al. 2023; Norén and Hasselgren 2025). The net outcome depends on factors such as the direction and rate of gene flow, the adaptive landscape, and population demographic contexts (Pfennig 2021). Genetic rescue—defined as the introduction of new genetic variation into inbred populations through assisted migration—can alleviate the negative genetic consequences of population fragmentation and has been successfully implemented in several species (Whiteley et al. 2015). However, concerns regarding outbreeding depression, in which admixed offspring exhibit reduced fitness, have limited the application of this approach (Tengstedt et al. 2025). To address these concerns, Frankham et al. proposed a framework for assessing the risks of outbreeding depression based on the presence or absence of major chromosomal polymorphisms, reproductive isolation, and local adaptation (Frankham et al. 2011). Genomic analyses suggest anthropogenic disruption of postglacial biogeographic patterns in European *A. mellifera* , with > 50% of populations now carrying hybrid genomes (Meixner et al. 2015). Such disruption erodes locally adapted gene pools and drives the loss of unique subspecies, a conflict that is particularly acute in biodiversity hotspots harboring evolutionarily distinct lineages (Requier et al. 2019).

*A. mellifera* is native to Europe, Africa, and western Asia (Han et al. 2012). Ruttner (1988) compiled the most comprehensive classification of its geographic variation, grouping subspecies into four evolutionary lineages using morphometry: A in Africa, C in Europe, M in Eurasia, and O in Asia (Ruttner 1988). Modern molecular techniques have largely validated this framework (Dogantzis et al. 2021; Franck et al. 2000). Subsequent studies have expanded the system, adding Y (Arabian Peninsula), L (Egypt), and U (Madagascar) lineages, along with newly described subspecies of *A. mellifera* (Dogantzis et al. 2021; Wallberg et al. 2014). As the most ocean‐distant mountain system and the largest range within global arid regions, the Tianshan Mountains in Central Asia represent the easternmost limit of *A. mellifera* 's native distribution, harboring two subspecies: *A. m. sinisxinyuan* of M lineage and *A. m. pomonella* of O lineage (Chen et al. 2016; Sheppard and Meixner 2003). Spanning 2500 km across China, Kazakhstan, Kyrgyzstan, and Uzbekistan, the Tianshan mountain ranges form one of the world's seven major mountain systems (Li et al. 2025). This region thus offers a natural laboratory for studying adaptation in native honeybees and their competition with managed bees introduced into protected habitats. One prominent example is the Xinjiang black honeybee (*XJ*) (Figures S1 and S2), a managed population introduced to the Yili Valley by Russia in the 1920s, Designated a Chinese National Animal Genetic Resource in 1990 (Figure S3) (Xu et al. 1986), *XJ* is conserved for its high foraging efficiency, which is approximately 2.4 times that of *Apis mellifera ligustica* , and greater cold tolerance than *A. m. carnica* (Xu et al. 1986). *XJ* exhibits distinct phenotypic and behavioral traits adapted to its harsh local environment (Gen 2019; Xu et al. 1986). Morphologically, the queens are typically large‐sized, with a predominantly black integument, exhibiting reddish‐brown (or tawny) bands on the abdomen (Ge 1993; Gen 2019; Xu et al. 1986). Key morphometric parameters include a proboscis length of 6.05 ± 0.11 mm, forewing length of 9.39 ± 0.07 mm, and cubital index of 1.69 ± 0.19 (Gen 2019). Behaviorally, *XJ* shows strong reproductive performance, characterized by a high egg‐laying rate and brood area coverage typically exceeding 90% of the comb surface (Gen 2019; Xu et al. 1986). It also exhibits remarkable overwintering capacity, surviving temperatures below −40°C with colonies as small as 4–6 frames, and shows a well‐defined annual reproductive cycle of 5–7 months (Ge 1993; Gen 2019; Xu et al. 1986). These adaptive traits highlight its ecological resilience and raise important questions regarding the genomic and evolutionary mechanisms underlying such local adaptations. Now, The Yili River Valley harbors approximately 60,000 managed *XJ* colonies. However, the underlying molecular mechanisms remain unclear, and its taxonomic status remains unresolved (Gao et al. 2005). Furthermore, its expansion creates another conservation paradox: while supporting regional apicultural economies, *XJ* threatens sympatric native subspecies through resource competition and genetic introgression. To clarify the evolutionary history and genomic interactions of *XJ*, we combined whole‐genome resequencing of 19 *XJ* workers with published genomic data from 172 individuals across 20 global populations. Using analyses of phylogeny, genetic diversity, inbreeding, and introgression (Hahn and Hibbins 2019), we aimed to resolve its taxonomic status, evolutionary trajectory and adaptive introgression. Our findings show that the present *XJ* population structure reflects multiple introgression events. These insights are critical for evaluating *XJ*'s debated classification, understanding its interactions with *A. m. sinisxinyuan* (M lineage) and *A. m. pomonella* (O lineage), and developing conservation strategies that distinguish managed from native populations. Such insights can help to protect both apiculture and the unique genetic heritage of native honeybees.

## 2 Results

### 2.1 Whole‐Genome Resequencing, Mapping, and Single‐Nucleotide Polymorphisms (SNPs)

**FIGURE 1:**
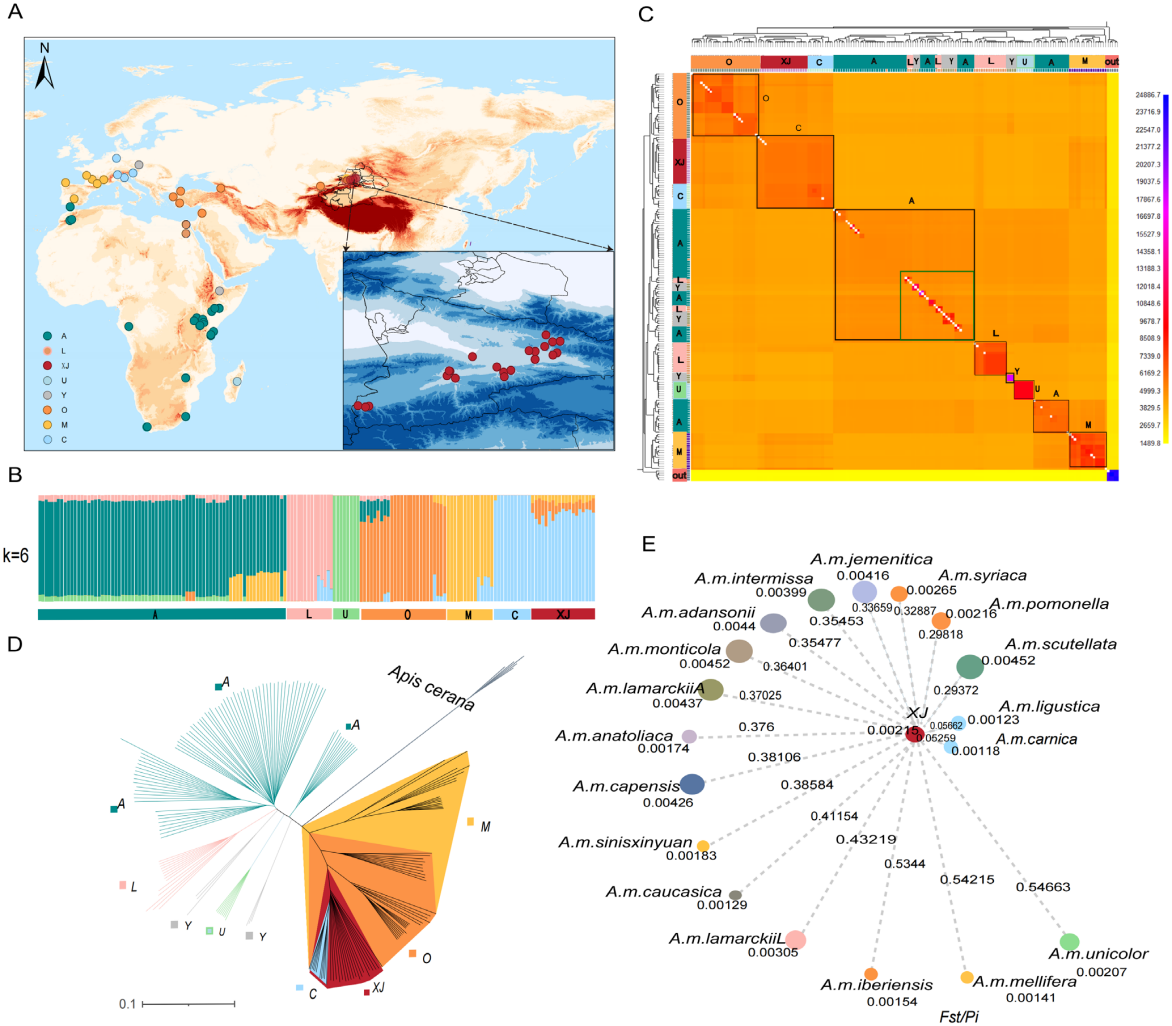
Population genomic structuring and relationships among *XJ* and *A. mellifera* lineages distributed globally. Geographic distribution of *XJ* and other *A.mellifera* samples analyzed in this study. Different colors represent distinct genetic lineages as indicated in the legend. The inset shows a detailed view of the *XJ* region and surrounding areas. Map depicting the locations of *XJ* and other *A.mellifera* samples analyzed in this study. Colors correspond to lineages. ADMIXTURE analysis results for *K* = 6, displaying individual ancestry proportions with low cross‐validation error. The analysis recovered six distinct genetic clusters corresponding to A (dark green), L (pink), U (light green), M (orange), O (yellow), and C (light blue) lineages. *XJ* is on the far right and consistently clusters with C lineage, distinct from other populations. FineSTRUCTURE co‐ancestry matrix visualized as a heatmap, where color intensity represents the degree of shared genetic ancestry from high (blue) to low (yellow) based on whole‐genome SNP data. Sample IDs are color‐coded according to their population origin, with distinct clusters corresponding to different *A.mellifera* lineages. Black‐outlined squares highlight the major genetic clusters representing distinct lineages (O, C, A, L, Y, U, M), as indicated in the legend. The matrix also showed higher shared co‐ancestry between *XJ* and C lineage. Maximum likelihood (ML) phylogenetic tree reconstructed from genome‐wide SNP data, illustrating the evolutionary relationships between *XJ* and other *A.mellifera* lineages. *Apis cerana* was used as the outgroup to root the tree. *XJ* clusters parallel to C lineage, supporting its close evolutionary relationship with this lineage. The scale bar represents genetic distance (0.1). Population genetic differentiation and diversity for *A.mellifera* lineages. Each circle represents a regional population, with circle size indicating the magnitude of genetic diversity (Θπ) and circle labels displaying the corresponding (Θπ) values. The length of connecting lines to *XJ* represents the degree of genetic differentiation (FST), with FST values labeled on each connecting line. *XJ* exhibits significantly elevated FST values with sympatric Tianshan subspecies *A. m. pomonella* and *A. m. sinisxinyuan*, indicating substantial genetic differentiation from these native bees.

We resequenced the genomes of 19 *XJ* workers from Yili (Table 1; Figure S1). This generated 32 GB of clean data with mean coverage of 97.08% and an average sequencing depth of 11.93× (Table S1). Five *Apis cerana* genomes were used as the outgroup. In addition, we then downloaded a global collection of 172 genomes from the Sequence Read Archive (Figure 1A, Table S2). Based on kinship analysis (Figure S4), we excluded 26 samples due to hybridization, first‐degree relatedness, or being haploid drones (Tables S3 and S4). In total, genotype calls or likelihoods were generated for 166 samples (including 19 *XJ*), yielding 20,639,768 high‐quality SNPs after stringent filtering.

**TABLE 1:** Information on the 19 samples of Xinjiang black honeybee (*XJ*) populations collected in different regions in Xinjiang, China.

	Sample name	Longitude	Latitude	County	Date
1	*XJ*	83.253	43.561	Xinyuan	2021.5.2
2	*XJ*	83.619	43.535	Xinyuan	2021.5.3
3	*XJ*	83.543	43.714	Nilek	2021.5.3
4	*XJ*	83.432	43.827	Nilek	2021.5.3
5	*XJ*	83.67	43.722	Nilek	2021.5.3
6	*XJ*	82.197	43.473	Gongliu	2021.5.7
7	*XJ*	82.607	43.304	Gongliu	2021.5.7
8	*XJ*	82.721	42.223	Gongliu	2021.5.7
9	*XJ*	82.731	43.148	Gongliu	2021.5.7
10	*XJ*	82.843	43.251	Gongliu	2021.5.8
11	*XJ*	80.456	42.668	Zhaosu	2021.5.9
12	*XJ*	80.272	42.677	Zhaosu	2021.5.9
13	*XJ*	80.396	42.656	Zhaosu	2021.5.9
14	*XJ*	81.768	43.163	Tekes	2021.5.9
15	*XJ*	81.909	43.123	Tekes	2021.5.9
16	*XJ*	81.82	43.236	Tekes	2021.5.9
17	*XJ*	81.804	43.242	Tekes	2021.5.9
18	*XJ*	83.549	43.499	Xinyuan	2021.5.7
19	*XJ*	83.149	43.571	Xinyuan	2021.5.7

### 2.2 Population Structure and Divergence

To examine the evolutionary history of *A. mellifera* , we used ADMIXTURE analysis and maximum‐likelihood (ML) phylogeny (Figure 1B). Cross‐validation indicated that the optimal number of genetic clusters was six (*K* = 6) (Figure S5). At *K* = 6, A, U, L, M, O, and C lineages were recovered as distinct clusters. Notably, Y lineage was not resolved as an independent cluster at this K value, likely due to its genetic similarity to A lineage and the relatively small sample size available for Y lineage in our dataset. A subtle signal of low‐level ancestry from the M lineage was detected in some A lineage individuals. In all analyses, the C lineage and *XJ* clustered consistently and were distinct from other populations.

To obtain a finer‐scale population structure, we applied fineSTRUCTURE to assess complex population assignments across values of K. The co‐ancestry matrix (Figure 1C) again identified A, U, L, Y, M, O, and C lineages, although population boundaries between Y and A lineages were less distinct. The matrix also showed higher shared co‐ancestry between *XJ* and C lineage. Next, using the whole‐genome SNP dataset, ML analysis in TreeMix resolved the major A, U, L, Y, M, O, and C lineages (Figure 1D). The *XJ* population was positioned parallel to C lineage. Taken together, these results confirm that the ancestral origin of *XJ* lies within C lineage.

To quantify divergence between *XJ* and subspecies of *A. mellifera* , we assessed genome‐wide differentiation (FST) (Figure 1E). The analysis revealed low FST values between *XJ* and *A. m. carnica* (0.05259) as well as *A. m. ligustica* (0.05662), indicating close genetic affinities. By contrast, significantly higher FST values were observed between *XJ* and the two sympatric subspecies in the Tianshan Mountains—*A. m. pomonella* (O lineage; FST = 0.29818) and *A. m. sinisxinyuan* (M lineage; FST = 0.38584)—highlighting substantial genetic differentiation from these native bees. Lineage‐level FST showed the same pattern (Figure S6, Table S6). Collectively, these results confirm that the ancestral origin of *XJ* lies within C lineage and that it is genetically distinct from the sympatric native subspecies, highlighting the necessity to examine how demographic history and human management have shaped its current genomic landscape.

### 2.3 Linkage Disequilibrium (LD) and Genomic Diversity

**FIGURE 2:**
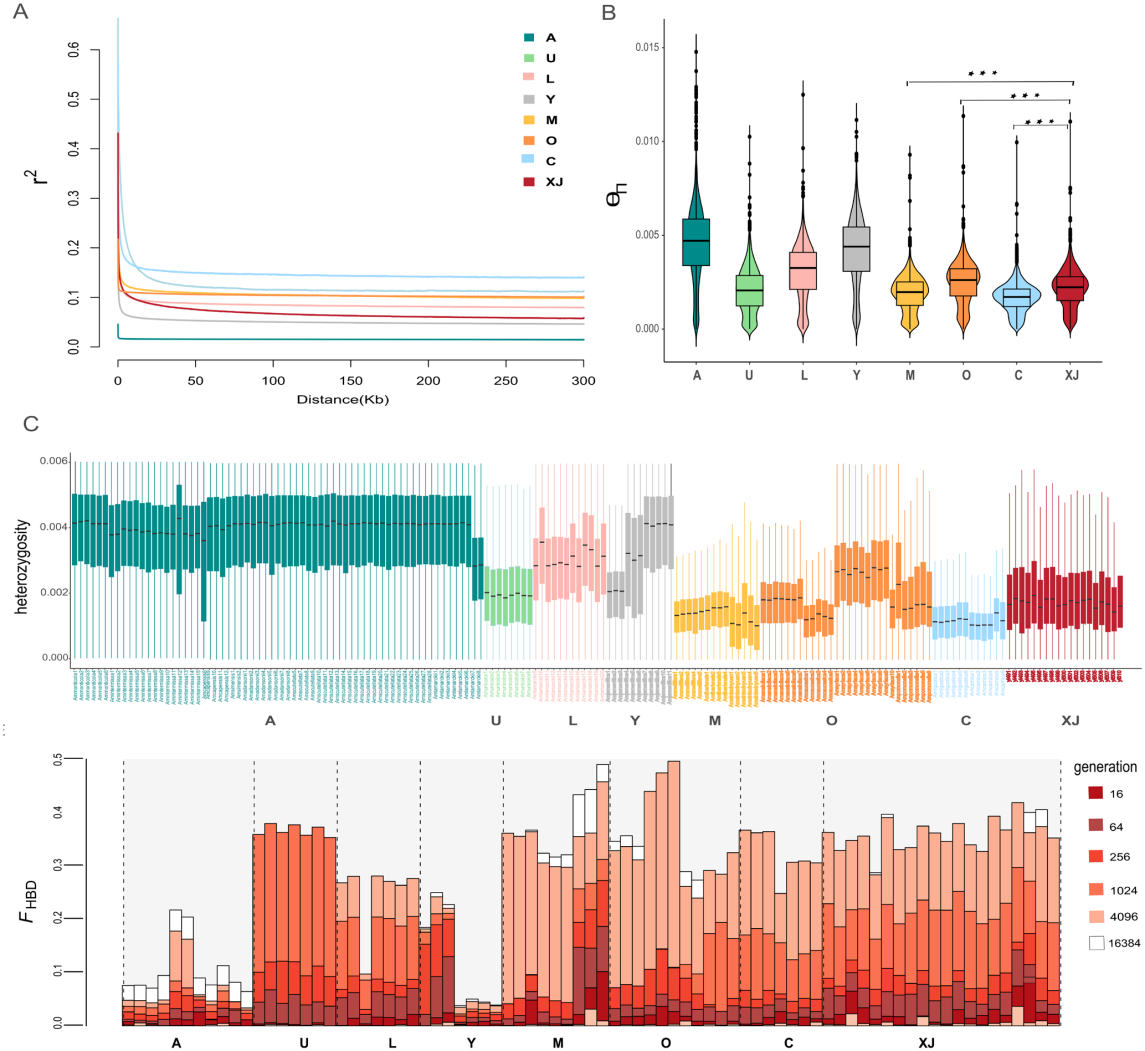
Genomic landscape of *XJ* and *A.mellifera* lineages. Linkage disequilibrium (LD) decay patterns across different *A.mellifera* lineages. LD values, represented as squared allele frequency correlations (*r* ^2^), are plotted against the physical distance between polymorphic sites. Each colored line corresponds to a distinct lineage: A (dark green), U (light green), L (pink), Y (gray), M (yellow), O (orange), C (light blue), and *XJ* (red). Notably, *XJ* exhibits faster LD decay (*r* ^2^ = 0.248 at 102 kb) compared to its ancestral C lineage, suggesting a unique demographic expansion following its divergence from C. Distribution of θΠ for *XJ* and *A.mellifera* lineages, assessed using Tracy–Widom test and calculated in nonoverlapping 10‐kb windows. Three asterisks indicate nonpaired Welch two‐sample *t*‐test *p* < 10^−3^. Notably, *XJ* exhibits a distinctΘπdistribution pattern compared to other lineages, with significantly higher values (*p* < 10^−3^) relative to its ancestral C lineage, suggesting unique genomic characteristics and evolutionary dynamics. Heterozygosity of *A.mellifera* populations. Colored lines represent different populations; higher He values reflect greater genetic diversity. Notably, *XJ* exhibits a distinct pattern with higher heterozygosity than its ancestral C lineage. Distribution of homozygosity by descent (HBD) segments across different *A.mellifera* lineages. The y‐axis represents *F*_HBD_ values, indicating the proportion of the genome covered by HBD segments. Different colors represent HBD segments of varying lengths: 16 (dark red), 64 (brown), 256 (red), 1024 (orange), 4096 (light orange), and 16,384 (white) generations. This study analyzed include A, U, L, Y, M, O, C, and *XJ*.

To infer the demographic history and management impacts on the Xinjiang black honeybee (*XJ*) genome, we analyzed Linkage Disequilibrium (LD) decay patterns. By calculating the squared allele frequency correlation (*r* ^2^) between SNP pairs, we found distinct patterns among the lineages (Figure 2A). A and Y lineages were characterized by a rapid decay, with *r* ^2^ dropping to half its maximum value and reaching a low of 0.0319 at just 16 kb. This pattern strongly suggests that A and Y have maintained a large effective population size or experienced recent expansion. In stark contrast, U lineage exhibited a substantially slower LD decay relative to the others, a classic genomic signature of a historical bottleneck or a prolonged period of small population size. Furthermore, the faster LD decay observed in *XJ* (*r* ^2^ = 0.248 at 102 kb) compared to its ancestral C lineage suggests a unique demographic expansion following its divergence from C. These findings provide important insights into *XJ*'s evolutionary history, particularly regarding its demographic expansion after diverging from C lineage, which may be related to *XJ*'s adaptation and dispersal in specific environments. Next, we determined nucleotide diversity (Θπ) for *A. mellifera* (Figures 1E and 2B). The diversity of *XJ* (Θπ = 2.15 × 10^−3^) was slightly higher than that of C lineage (Θπ = 1.66 × 10^−3^). At the population level, Θπ of *XJ* exceeded that of *A. m. carnica* (Θπ = 1.18 × 10^−3^) and *A. m. ligustica* (Θπ = 1.23 × 10^−3^). Thus, relative to these C lineage subspecies, *XJ* exhibited greater diversity. Meanwhile, when compared with sympatric subspecies, *XJ* exhibited similar genetic diversity to *A. m. pomonella* (O lineage) (Θπ = 2.16 × 10^−3^) but showed higher nucleotide diversity than *A. m. sinisxinyuan* (M lineage) (Θπ = 1.83 × 10^−3^). This pattern of elevated diversity in *XJ* aligns with and genetically substantiates the demographic expansion inferred from its LD decay profile, highlighting how its unique population history has shaped a broader standing genetic variation.

### 2.4 Inbreeding and Heterozygosity Analysis

Artificial populations are likely to increased inbreeding, which manifests as long, uninterrupted homozygous tracts known as Identical‐by‐descent (IBD) segments (Ferenčaković et al. 2017). These genomic patterns act as measurable indicators for evaluating inbreeding levels. Here, we used two methods to estimate the fraction of the genome within IBD segments (FIBD) (Gibson et al. 2006): (i) FHBD calculated from homozygous‐by‐descent (HBD) segments from model‐based approaches RZooRoH, and (ii) FROH estimated from runs of homozygosity (ROHs) using observational‐based approaches (Figure 2D, Figure S7). A lineage (African lineage) displayed the lowest homozygous segment proportion (total proportion < 0.5), with minimal representation across all segment lengths. U lineage exhibited the highest proportion of homozygous segments (total proportion ~1.2), with long segments (512–1024 generations) predominating, indicating sustained historical inbreeding by island isolation. In the HBD segment analysis, lineages M and C were clearly separated. However, within lineage M, the inbreeding outcomes were inconsistent between the European *A. m. mellifera* and the Tianshan *A. m. sinisxinyuan*. The latter subspecies showed the second‐highest homozygous segment proportion (total proportion ~1.0), with a higher prevalence of short segments (16–256 generations). C lineage (ancestral lineage of *XJ*) demonstrated a moderate homozygous segment proportion (total proportion ~0.8), with a relatively higher proportion of long segments (512–1024 generations). Comparative analysis revealed that *XJ* population showed a lower homozygous segment proportion than M lineage, but it remained higher than the C lineage average, characterized by a higher proportion of short segments (16–256 generations).

Heterozygosity (H) is a fundamental and powerful metric for assessing genetic diversity in natural populations. Populations with lower genetic diversity naturally exhibit higher inbreeding coefficients, reflecting the genetic impact of small effective population sizes and non‐random mating (Kanaka et al. 2023). In this study, lineages exhibited varying heterozygosity levels (Figure 2C): A (0.0040), L (0.0035), O (0.0029), XJ (0.0028), Y (0.0026), U (0.0021), M (0.0020), and C (0.0015). We observed a negative correlation between heterozygosity and inbreeding coefficients (Figure 2D). Lineages with high heterozygosity, such as lineage A (0.0040), exhibited low inbreeding, whereas those with low heterozygosity, such as lineage C (0.0015), showed high inbreeding. Within this global context, the *XJ* population displayed a distinct genetic pattern. Although its heterozygosity value (0.0028) falls within the global range of *A. mellifera* , it is higher than the C lineage average (0.0015). Notably, *XJ* also exhibited higher inbreeding coefficients compared to other C lineage subspecies. This pattern suggests that the demographic expansion inferred for *XJ* may have introduced new genetic variation, increasing overall heterozygosity, while its population structure or recent history involved processes that led to a detectable excess of homozygosity.

### 2.5 Detecting and Quantifying Genome‐Wide Introgression

**FIGURE 3:**
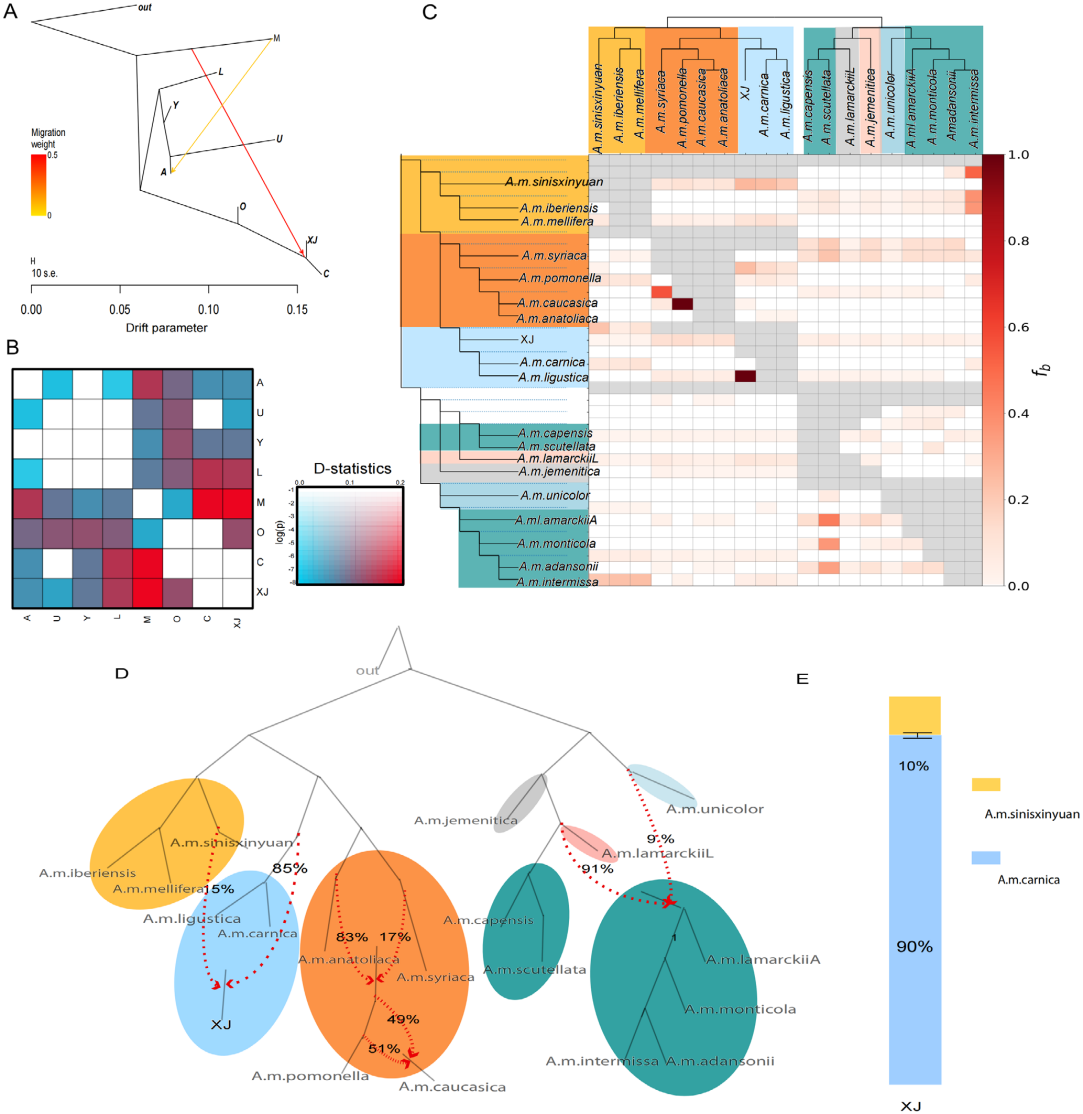
Gene flow analysis among *A.mellifera* populations using multiple population genetic methods. Gene flow was determined using TreeMix; branch lengths are proportional to experienced genetic drift. *Apis cerana* rooted the tree. Scale bar = ten SE units from the sample covariance matrix. Migration weight indicates ancestry proportion from the migration edge. Gene flow detected using ABBA–BABA statistics (Dsuite). The heatmap displays D‐statistics values, with color intensity representing the magnitude of gene flow between lineages. D‐statistics confirmed significant admixture between M lineage and *XJ* (|Z| > 3), supporting gene flow from M lineage into ancestors of *XJ*. Gene flow determined using the F‐branch method. The heatmap shows f‐branch values, indicating the extent of gene flow between different *A.mellifera* subspecies and lineages. *XJ* displays characteristic gene flow signatures that distinguish it from other analyzed populations, reflecting key evolutionary events including gene flow from sympatric *A. m. sinisxinyuan* (M lineage) into the ancestral population of *XJ*, which originated from C lineage. Introgression from O lineage (*A. m. pomonella*) was also detected. Introgression and mixture proportions among *A.mellifera* subspecies from qpGraph. The phylogenetic network illustrates complex admixture patterns, with percentages indicating the proportion of ancestry from different source populations. *XJ* shows a mixed ancestry profile, with 85% contribution from ancestral C lineage and 15% admixture from M lineage. Introgression from *A. m. sinisxinyuan* into *XJ* estimated using qpAdm. The bar chart quantifies the proportion of ancestry, showing that *XJ* derives approximately 10% of its genome from *A. m. sinisxinyuan* and 90% from *A. m. carnica*, confirming significant gene flow from the sympatric *A. m. sinisxinyuan*.

To resolve the paradoxical genomic features of *XJ*, we conducted a comprehensive analysis of gene flow. Results revealed a complex history of introgression, with *XJ* receiving contributions from both M lineage and other C lineage subspecies (Figure 3, Table S7). Evidence of migration was evaluated in TreeMix using 1–10 migration edges. OptM identified two migration events as optimal (Δ = 2000, variance explained = 99.8%) (Figure S8): (1) gene flow from M lineage into ancestors of *XJ*, and (2) additional gene flow from M lineage into A lineage (Figure 3A), consistent with the result in the ADMIXTURE analysis (Figure 1B). D‐statistics confirmed significant admixture between M lineage and *XJ* (|Z| > 3, Figure 3B). F‐branch analysis localized introgression signals to specific branches, identifying distinct phases of gene flow among *A. mellifera* subspecies (Figure 3C). A key event was gene flow from the sympatric *A. m. sinisxinyuan* (M lineage) into the ancestral population of the Xinjiang black honeybee, which originated from C lineage. Additionally, introgression from O lineage (*A. m. pomonella*) was also detected. Furthermore, f3‐ratio tests revealed another introgression event between *XJ* and the commercially managed C lineage subspecies *A. m. ligustica*, with an admixture proportion exceeding 20% (Figure 3C; Figure S9). Together, these results show that multiple introgression events have shaped the *XJ* genome.

**FIGURE 4:**
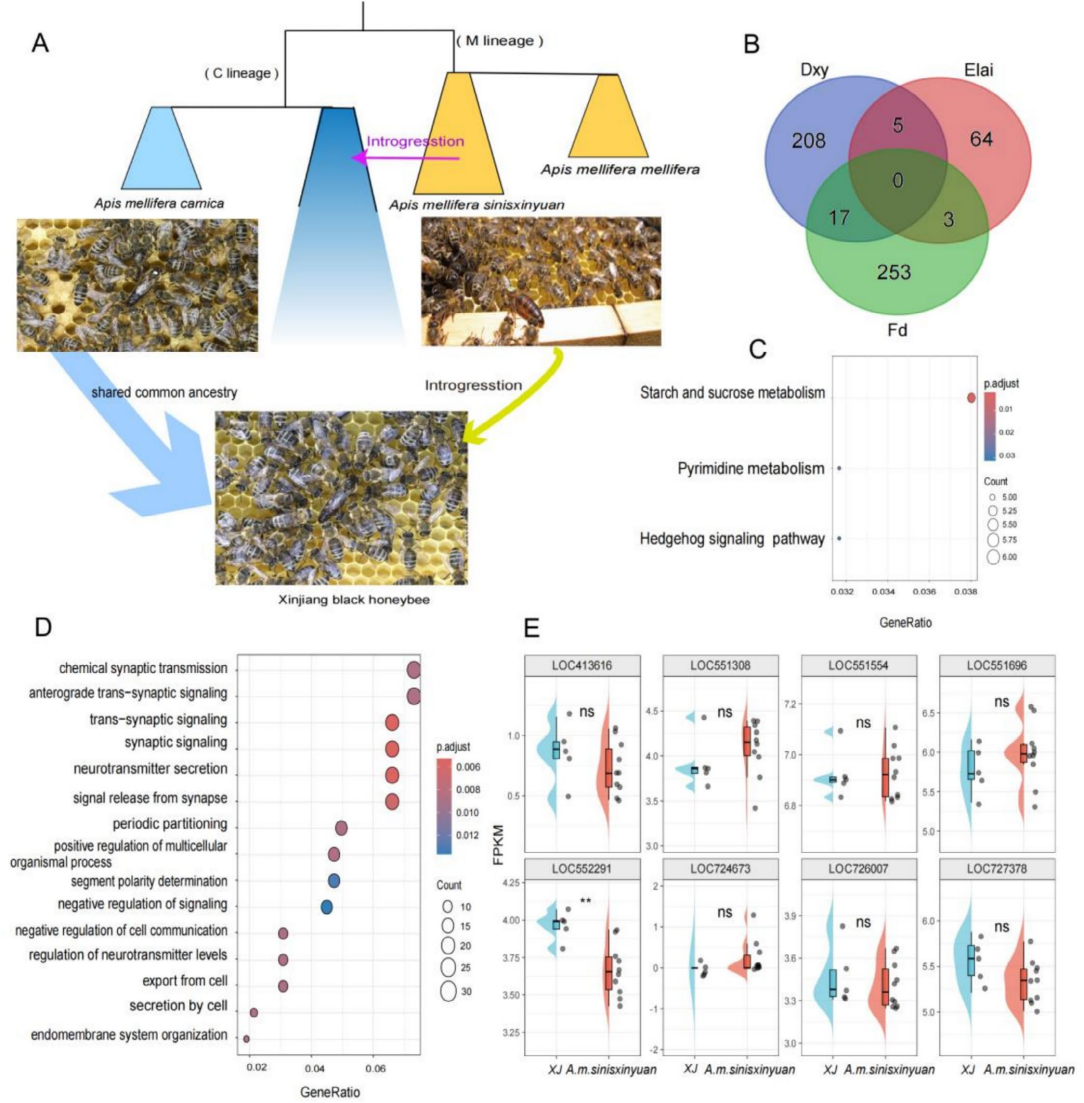
Evolutionary history and functional annotation of introgressed genes in Xinjiang black honeybee (*XJ*). Phylogenetic relationships and admixture pattern of *XJ*. This panel focuses on the evolutionary history of Xinjiang black honeybee (*XJ*), illustrating its genetic origins from two distinct lineages: *A. m. carnica* (C lineage, blue gradient) and *A. m. sinisxinyuan* (M lineage, yellow gradient). The diagram illustrates the introgression event (yellow arrow) that occurred between these lineages, with the honeycomb images providing visual context of the different honeybee populations studied. Venn diagram of introgressed genes. This analysis identifies genes with evidence of introgression using three population genetic statistics: Dxy (blue), Elai (red), and Fd (green). The overlapping regions represent genes with consistent signals of introgression across multiple metrics, with specific counts indicating the number of genes in each category (208, 64, 253, 17, 5, and 3 genes respectively). KEGG pathway enrichment analysis. The enrichment plot shows significantly enriched metabolic pathways among shared introgressed genes, including starch and sucrose metabolism, pyrimidine metabolism, and Hedgehog signaling pathway. The color gradient and dot size represent the statistical significance and enrichment ratio of each pathway. GO (Gene Ontology) enrichment analysis. This panel highlights biological processes enriched among introgressed genes, with particular emphasis on synaptic transmission‐related pathways including chemical synaptic transmission, anterograde trans‐synaptic signaling, and neurotransmitter secretion. The dot plot displays the enrichment ratio (GeneRatio) and statistical significance (p‐adjusted values) for each GO term. Differential gene expression analysis of eight candidate genes (LOC413616, LOC551308, LOC551554, LOC551696, LOC552291, LOC724673, LOC726007, and LOC727378) between two *A. mellifera* populations (*XJ* vs. *A. m. sinisxinyuan*) using FPKM values. Each panel represents a single gene, with boxplots showing the distribution of FPKM values for each population (blue: *XJ*; red: *A. m. sinisxinyuan*). Plots overlay the boxplots to visualize data density. Scatter points indicate individual sample measurements. *t*‐test statistical significance is denoted by asterisks: Ns indicates no significant difference (*p* > 0.05), while ** denotes a highly significant difference (*p* < 0.01).

We further quantified introgression using Qpgraph and qpAdm (Figure 3E; Figure S10, Table S8) and expanded analysis with ADMIXTOOLS2, allowing up to four migration events. The optimum tree model had a likelihood score of 248774.84, with the worst residual of approximately 100.3 (Figure S10). Introgression analyses indicated 15% admixture from M lineage into *XJ*, with 85% from ancestral C lineage. At the subspecies level, qpAdm modeling estimated that the *XJ* genome is derived from approximately 90% ancestry related to *A. m. carnica* (C lineage) and 10% from the sympatric native *A. m. sinisxinyuan* (M lineage) (|*Z*| = 33.02). These results confirmed that *XJ*'s present genome was shaped by contributions from both C and M lineages, with C providing the primary ancestry (Figure 4A). Considering that the *XJ* genome is predominantly derived from C lineage but retains critical adaptive alleles from the sympatric M lineage (*A. m. sinisxinyuan*), this population serves as a unique resource of local adaptation with significant potential for breeding applications.

### 2.6 Gene Ontology (GO) and Kyoto Encyclopedia of Genes and Genomes (KEGG) Annotation of Introgressed Genes

To investigate the functional impact of these putatively introgressed regions, we performed local ancestry inference analysis. We identified 550 candidate introgressed regions from the native subspecies *A. m. sinisxinyuan* (M lineage) into the *XJ* using at least one of three complementary statistical tests (*F*_d_, *D*xy, or Elai) (Table S9; Figure 4B). These regions were detected with 50‐kb sliding windows across the genome. Subsequently, we evaluated the genomic and phenotypic impacts of introgression based on the genes located within these regions. To explore functional enrichment, we identified significantly enriched GO terms. Significant enrichment was also found in chemical synaptic transmission (GO:0007268; *p*.value = 5.23E‐06), enzyme binding (GO:0019899; *p*.value = 3.47E‐06), and synapse (GO:0045202; *p*.value = 3.53E‐05) (Figure 4D, Table S10). Several genes located within these regions—including LOC413616, LOC726007, LOC724673, LOC551554, LOC551308, LOC551696, LOC727378, and LOC552291 (Table S11). Next, we conducted functional analysis using the Kyoto Encyclopedia of Genes and Genomes (KEGG) database. We found that several KEGG pathways were enriched, including starch and sucrose metabolism (dme00500; *p*.value = 2.50E‐03), Hedgehog signaling (dme04341; *p*.value = 2.55E‐02), and pyrimidine metabolism (dme00240; *p*.value = 3.12E‐02) (Figure 4C, Table S12). Notably, enrichment in starch/sucrose metabolism and Hedgehog signaling suggests potential adaptive benefits related to energy utilization and developmental regulation in the cold Tianshan environment.

### 2.7 Transcriptomic Expression Analysis

To provide functional validation for these genomic findings, we conducted transcriptome analysis on 15 adult worker bees—5 from *XJ* and 10 from *A. m. sinisxinyuan* colonies—all sampled during the peak foraging season. We assessed the expression levels of candidate genes within the introgressed regions. Notably, we found that LOC552291 (encoding DNA replication licensing factor MCM4) (Sheu et al. 2014) was significantly differentially expressed between populations (Figure 4E). MCM4 modulates DNA replication in response to environmental stress. Its differential expression provides preliminary transcriptional‐level support for the potential functional relevance of this introgressed locus. The enrichment of pathways related to starch/sucrose metabolism and Hedgehog signaling at the genomic level, combined with the differential expression of key candidates like MCM4, suggests potential adaptive advantages related to energy utilization and developmental regulation in the cold Tianshan environment. We emphasize that these expression data support but do not conclusively prove adaptive benefits, which would require future physiological validation.

## 3 Discussion

Our study presents a comprehensive genomic analysis of *XJ*, a managed population of *A. mellifera* introduced to the Tianshan Mountains—a global biodiversity hotspot and the easternmost frontier of the species' natural range—approximately one century ago (Xu et al. 1986). This population now poses a complex conservation paradox. By integrating whole‐genome resequencing with advanced population genomic analyses, we resolved the debated evolutionary history of *XJ* and quantified its genetic interactions with two native subspecies, *A. m. sinisxinyuan* (M lineage) and *A. m. pomonella* (O lineage). Our results reveal that *XJ* is not merely a product of human‐mediated introduction and selection but also a compelling example of putatively adaptive introgression (Figure 4; Table S11). Beneficial alleles introgressed from *A. m. sinisxinyuan*, particularly those linked to cold tolerance and foraging, are consistent with rapid local adaptation. This genomic architecture explains the paradoxical combination of high genetic diversity and elevated inbreeding observed in *XJ*, wherein adaptation appears to have been facilitated by the introgression of beneficial alleles from native bees. These findings necessitate a re‐evaluation of *XJ*'s taxonomic status and support the adoption of differentiated conservation strategies. Such strategies should distinguish managed from native populations and include measures such as genomic monitoring of introgression and designation of conservation zones for native subspecies.

### 3.1 Genomic Landscape of *XJ*

The genomic landscape of *XJ* presents an intriguing paradox that reflects its unique history. It shows higher nucleotide diversity (Θπ) and heterozygosity than its presumed ancestral C lineage (e.g., *A. m. carnica* and *A. m. ligustica*), yet carries signatures of inbreeding, indicated by extensive ROH (Figure 2). This paradox reflects two opposing forces: artificial selection by beekeepers and natural gene flow from sympatric populations (Matias et al. 2017). Elevated diversity and heterozygosity appear to result largely from substantial introgression from *A. m. sinisxinyuan* (M lineage) and additional gene flow from managed C lineage subspecies, as supported by TreeMix, F‐branch, and qpAdm analyses (Figure 3). These introgression events likely provided novel variation, counteracting diversity loss during domestication (Benson et al. 2011). Conversely, the elevated inbreeding coefficient (FROH) is consistent with modern breeding practices, which rely on a limited number of queens and controlled mating, increasing genome‐wide homozygosity (Blacquière and Panziera 2018). As a result, the *XJ* genome reflects a complex architecture shaped jointly by anthropogenic inbreeding and adaptive introgression, complicating its taxonomic classification.

While our genome‐wide analyses provide a comprehensive overview, we acknowledge that averaging across the entire genome can mask localized signals. For instance, long runs of homozygosity (ROH) indicative of very recent inbreeding or selection may be diluted in whole‐genome LD estimates. Our reliance on global metrics and standard population genomic models carries the inherent risk of overlooking such heterogeneous patterns. Future studies employing targeted scans for ROH or sliding‐window selection analyses will be valuable to uncover potential ‘hidden’ signatures of selection and validate the broader patterns identified here.

### 3.2 Adaptive Introgression and Local Colonization

While the effects of introgression from native relatives and management on local populations have been studied extensively in mammals at the genome‐wide scale, invertebrates remain comparatively overlooked (Chen et al. 2023; von Seth et al. 2021; Yu et al. 2020). In this study, introgression from the native *A. m. sinisxinyuan* into *XJ* appears nonrandom and adaptive, likely conferring a selective advantage in the challenging Tianshan environment. Functional annotation suggests that LOC413616 and LOC726007, which are related to developmental processes such as exoskeletal system, may encode a protein that binds histone peptides monomethylated at lysine residues, potentially regulating the expression of homeotic genes during development (Shelomi et al. 2014). LOC724673 is predicted to be associated with the olfactory sensory system, suggesting a potential role in olfactory perception (Horgue et al. 2022). LOC551554 and LOC551308 are linked to cell proliferation (Evans and Huang 2018). LOC551696 and LOC727378 participate in key signaling mechanisms involved in diverse physiological and pathological processes (Celi et al. 2022). LOC552291 (encoding MCM4) modulates DNA replication in response to environmental stress (Sheu et al. 2014). While transcriptomic validation strengthens our functional inference, future work should integrate physiological assays (e.g., cold survival tests) to directly link MCM4 expression to adaptive phenotypes. Functional enrichment analysis of introgressed genomic regions revealed significant overrepresentation of genes in pathways critical for survival in cold, high‐altitude habitats (Information 2025). Notably, enrichment of starch and sucrose metabolism pathways suggests enhanced metabolic capacity for energy storage and use, a trait essential for surviving prolonged winters (Keaveny et al. 2023). This observation aligns with documented cold tolerance of *XJ* relative to other C lineage subspecies (Xia 1986; Xu et al. 1986). In addition, introgressed alleles associated with the Hedgehog signaling pathway, a key regulator of development and cellular homeostasis, indicate potential roles in developmental stability and stress resistance (Busson and Pret 2007; Jacob and Lum 2007). Enrichment of olfactory receptor genes further suggests altered olfactory capability (Barish and Volkan 2015; Mika et al. 2021), which could influence foraging efficiency, consistent with reports of *XJ* exhibiting approximately 2.4‐fold higher efficiency than *A. m. ligustica* (Xu et al. 1986). Taken together, these findings support a model in which establishment and expansion of *XJ* (C lineage) in the Tianshan region were facilitated, at least in part, by adaptive alleles introgressed from native *A. m. sinisxinyuan* (M lineage) (von Seth et al. 2021). This case illustrates how human‐mediated introductions may shape novel evolutionary trajectories, enabling introduced species to increase fitness through genetic input from local populations.

### 3.3 Taxonomic Implications and Conservation Considerations

The complex genomic history of *XJ* challenges traditional taxonomic classification and raises significant conservation concerns (Gao et al. 2005). Phylogenomic analyses place *XJ* within C lineage, most closely related to *A. m. carnica*. However, a substantial contribution from M lineage (~10%–15%) distinguishes *XJ* from described *A. mellifera* subspecies, which are generally treated as distinct evolutionary lineages with limited historical gene flow. Thus, classifying *XJ* as a pure C lineage subspecies may not be genetically accurate (Committee 2011; Lin et al. 2023). We propose instead that *XJ* be recognized as a regionally adapted, managed population characterized by significant genomic introgression. This interpretation, however, poses a conservation dilemma. *XJ* is designated a China National Animal Genetic Resource, reflecting its economic value and desirable traits (Committee 2011). While protecting this genetic resource is justified, promoting its expansion may conflict with conservation of the two native subspecies, *A. m. sinisxinyuan* (M lineage) and *A. m. pomonella* (O lineage). Our results demonstrate substantial divergence between *XJ* and these native subspecies (FST = 0.27 and 0.46, respectively), supporting it recognition as distinct Evolutionary Significant Unit. Continued expansion of *XJ* threatens to erode the genetic distinctiveness and adaptive complexes of these native *A. mellifera* subspecies through both resource competition and introgression (Xu et al. 1986; Zecherle et al. 2021), potentially leading to long‐term diversity loss (Kardos et al. 2018; Lobo et al. 2023). Conservation strategies must therefore balance preservation of a valuable managed population with protection of native genetic integrity.

### 3.4 Toward an Integrated Framework for Honeybee Conservation

The case of *XJ* in the Tianshan Mountains exemplifies a broader global challenge facing biodiversity in the Anthropocene (Aglagane et al. 2023; DeLory et al. 2019; Ellis et al. 2018; Groeneveld et al. 2020; Moritz et al. 2005; Requier et al. 2019; Waters and Turner 2022). Conventional frameworks often fail to differentiate between native populations, products of natural evolutionary processes and unique genetic reservoirs, and managed populations, which undergo artificial selection and admixture (Aglagane et al. 2023). A similar situation is seen in the Dongbei bee, another managed *A. mellifera* population formally recognized as a landrace in China. Similar to *XJ*, the Dongbei bee has significant economic value and is the focus of conservation measures, including establishment of a nature reserve in Raohe County. These cases highlight a critical gap in conservation practice: the absence of frameworks that distinguish between conserving native evolutionary lineages and safeguarding managed or admixed stocks (De la Rúa et al. 2009).

To address these issues, we propose an integrated, multi‐faceted strategy:

- *Establish genetic refuges for native subspecies*: Native *A. m. sinisxinyuan* and *A. m. pomonella* should be strictly protected. Protected areas within the Tianshan Mountains must prohibit introduction of managed colonies, serving as genetic refuges that reduce introgression and ecological competition while maintaining native integrity (Meixner et al. 2010).
- *Implement zoned management for managed populations*: In regions adjacent to refuges, *XJ* should be carefully regulated. Best management practices should minimize contact with native populations, including the use of locally adapted, selectively bred queens in controlled apiaries (Meixner et al. 2010; Parejo et al. 2025). This approach enables economic use of *XJ* while reducing incentives to establish colonies near protected habitats.
- *Adopt dynamic genomic monitoring*: Long‐term monitoring of *XJ*, *A. m. sinisxinyuan*, and *A. m. pomonella* is essential. Regular genomic assessments would track gene flow, detect emerging risks, and guide evidence‐based adjustments to conservation actions (Büchler et al. 2013; Cain et al. 2011). In summary, our study provides strong evidence of how human management has influenced the evolutionary trajectory of a key pollinator. *XJ* demonstrates how adaptive introgression can contribute to managed success but also highlights risks to endemic diversity. Meeting this challenge requires moving beyond species‐based conservation to frameworks that explicitly account for evolutionary lineages and genetic processes. By setting differentiated objectives for managed and native honeybees, strategies can be developed that sustain apiculture while preserving the long‐term genetic heritage of native honeybee diversity.

## 4 Materials and Methods

### 4.1 Sampling and Sequencing

We sampled 19 workers from managed apiaries located in the Tianshan Mountains of the Yili River Valley, a concentrated apicultural region. The sampling sites covered five counties—Nilek, Gongliu, Zhaosu, Tekes, and Xinyuan—with the following distribution: 3 colonies from Nilek, 5 from Gongliu, 3 from Zhaosu, 4 from Tekes, and 4 from Xinyuan (Table 1). While Nilka County serves as the core protected area, our sampling also included registered Xinjiang black honeybee apiaries in surrounding counties to capture the overall genetic landscape. *XJ* is a dark bee with black abdomens and gray hair, occasionally with brownish‐red bands on the abdomen, and worker bees have very long abdominal hair (Figure S2) (Xu et al. 1986). Most beekeepers are focused on the breeding of *XJ*. We sampled a minimum of five hives per apiary. Fifty workers were collected from each hive and preserved in 90% ethanol. From each hive, one individual was randomly selected for genomic DNA extraction. We performed whole‐genome sequencing for 19 *XJ* using an Illumina HiSeq 2500 with standard procedures. The 500 bp paired‐end libraries were prepared to generate approximately 11.93× raw coverage. A total of 32 GB of clean sequence data were obtained (Table S1), which were deposited in the NCBI Short Read Archive under the accession number PRJNA1040327.

To gain further insights into *XJ*, we also downloaded publicly available raw sequence data from Sequence Read Archive, SRA, which included 3.2 T of data from 172 individuals representing 20 populations (besides 5 outgroup). We also downloaded data for five *Apis cerana* as outgroup representatives (Table S2).

### 4.2 SNP Calling

All FASTQ data Adapter content was trimmed and 5 bp was removed from both the 5′ and 3′ ends of each read using Trim Galore v0.4.4 with default parameters (http://www.bioinformatics.babraham.ac.uk/projects/trim_galore/). Quality control was performed using FastQC v0.11.9 (http://www.bioinformatics.babraham.ac.uk/projects/fastqc/). Reads were mapped against the *A. mellifera* reference genome (Amel_HAv3.1) (Wallberg et al. 2019) using BWA‐MEM v0.7.17 (Li and Durbin 2009) with default settings. After mapping, the reads were re‐aligned around indels using GATK IndelRealigner (McKenna et al. 2010). Samtools v1.7 (Li et al. 2009) was used for sorting and duplicates were removed using the Picard Mark Duplicates tool (https://sourceforge.net/projects/picard/). Next, SNPs were identified by GATK best practice workflow for the joint genotyping strategy. We called variants for each sample using the Haplotypecaller module with the parameter “‐genotyping‐mode DISCOVERY ‐min‐base‐quality‐score 20 ‐stand‐call‐conf 30 ‐emit‐ref‐confidence GVCF.” Next, we conducted joint genotyping by combining all of the GVCFs using the GenotypeGVCFs module. (1) SNPs with mean depth (for all samples) < 1/3× and > 3× (×, overall mean sequencing depth across all SNPs); (2) quality by depth, QD < 2; (3) phred‐scaled variant quality score, QUAL < 30; (4) strand odds ratio, SOR > 3; (5) Fisher strand, FS > 60; (6) mapping quality, MQ < 40; (7) mapping quality rank sum test, MQRankSum < −12.5; and (8) read position rank sum test, ReadPosRankSum < −8 were filtered, and non‐biallelic SNPs were filtered out using VCFtools v0.1.15 (Danecek et al. 2011).

### 4.3 Population Genetic Structure

To gain insight into the population structure and phylogenetics of *A. mellifera* , evolutionary relationships among the local Xinjiang black honeybee and other samples of *A. mellifera* were reconstructed with a ML tree using SNPs located genome‐wide. To avoid sampling bias, based on a kinship coefficient, we evaluated the relatedness of each sample within each group using KING v2.1.3 (Table S3) (Manichaikul et al. 2010). Pairs with a kinship coefficient > 0.177 (twins and first‐degree relationship) were closely related individuals. Based on admixed, first‐degree kinship, or sample haploid, we excluded 26 samples (Table S4). ADMIXTURE v1.3 (Alexander et al. 2009) was used to perform a population structure analysis based on SNP genotypes from 166 samples with coancestry clusters ranging from *k* = 2 to *k* = 8 and ran the analysis 20 times for each k (Table S5). For ADMIXTURE analysis, we analyzed *A. mellifera* samples only. The optimal number of genetic clusters in the cross‐validation of the ADMIXTURE analyses was six (*K* = 6). To infer the phylogenetic relationships between the Xinjiang black honeybee and other populations, we constructed ML phylogenetic trees using TreeBeST v1.9.2 (http://treesoft.sourceforge.net/treebest.shtml) and RAxML7.0.4 (Stamatakis 2014).

Compared with the STRUCTURE‐like approach, to analyze single mutations individually, fineSTRUCTURE v4 (Leslie et al. 2015) used the relative positions of these mutations in the genome. To use much of the information present in haplotype structure and obtain fine‐scale population structure, we used chromoPainter and fineSTRUCTURE to find patterns of haplotype similarity (Lawson et al. 2012). The samples were phased using SHAPEIT4 (Delaneau et al. 2019) and missing data were eliminated before running fineSTRUCTURE v4 (Leslie et al. 2015). The supplied impute2chromopainter.pl. script was used to reformat the data independently for each chromosome‐level scaffold. Assuming a constant rate of recombination per base, recombination rate maps were generated using the makeuniformrecfile.pl. script. The fineSTRUCTURE GUI that was included with the program was used to visualize the results after running the program using default settings. With VCFtools v0.1.16, the distribution of pairwise fixation statistics (FST) was computed through a sliding window method based on 50‐kb sliding windows with a 25‐kb step size.

### 4.4 Genetic Diversity, Linkage Disequilibrium (LD)

An estimation of heterozygosity was made for each sample using ANGSD v0.93,889 (Korneliussen et al. 2014). For each population LD decay was calculated using Haploview v4.292 (Barrett et al. 2005). Nucleotide diversity (π) was estimated with VCFtools v0.1.15 (Danecek et al. 2011).

### 4.5 ROH and Individual Inbreeding Coefficients

To describe individual inbreeding into the approximate generation via model‐based methods (Druet and Gautier 2017), we also used the R package ZooRoH v0.3 (Bertrand et al. 2019) to characterize individual inbreeding by model‐based methods that utilize Hidden Markov Models (HMM) (Druet and Gautier 2022). ROH (runs of homozygosity segments within a single individual with shared paternal ancestry) analysis was done using PLINK v1.9 (Chang et al. 2015). This package assessed the contributions of various generation levels of inbreeding, and classified HBD segments into age‐based classes.

### 4.6 Divergence Histories and Introgression Analysis in *A. mellifera*

To gain further insight into the source of introgression, we employed various approaches to search for potential gene introgression between the *XJ* and other *A. mellifera* populations (Pickrell and Pritchard 2012). To identify the likely mixing event, we used TreeMix v1.13 (Pickrell and Pritchard 2012), D‐statistics, and f3‐statistics (Patterson et al. 2012). For TreeMix, we constructed an ML tree accounting for LD by grouping sites in blocks of 1000 SNPs (−k 1000) and set *Apis cerana* as the root. After constructing a tree, we added migration events and iterated the process 20 times for “‐m 1–10” to ensure convergence. The inferred trees were visualized with the TreeMix R script plotting function. To determine the optimal number of migration edges, we used the OptM R package (Fitak 2021). The Evano method was used to estimate the proportion of variance explained by different numbers of migration edges. The ad hoc statistic Δm was employed to determine the optimal number of migration edges, with the goal of explaining 98% of the variance between the breeds could be explained. When TreeMix allowed migration edges to be added, the optimal number of edges was selected as 2, which explained ~98% of the variance. D‐statistics and f3‐statistics were implemented in ADMIX tools v5.1 (Figure S9) (Patterson et al. 2012). We estimated D‐statistics based on the following scenarios: D (X, Y; Z, *Apis cerana* ), where X, Y, and Z were different permutations of the *A.mellifera* populations. The output provided BABA and ABBA counts, along with D‐statistics values, standard errors, and Z‐scores. Only positive D‐statistics values and |Z‐scores| ≥ 3 were considered to indicate admixture between population X and Z (Figure 3A,B). Further, we computed D‐statistics for each branch across alternative topologies using the F‐branch module. The resulting statistics were visualized with the dtools.py script, which is included in the Dsuite software package. Subsequently, we estimated the outgroup f*3*‐statisctics for the following: f3 (X; Y, *Apis cerana* ) where X and Y represented different permutations of *A.mellifera* populations. We considered f3‐statistics < 0 and a Z‐score less than −3 as being statistically significant, indicating historical events of an admixture of the X and Y populations (Figure 3C).

### 4.7 qpAdm and qpGraph

Admixtools2 (v2.0.0) (Patterson et al. 2012) is a new and fast version of Admixtools that performs an impartial comparison of any two qpgraph models using out‐of‐sample scores (https://uqrmaie1.github.io/admixtools/index.html). We chose one SNP per 100 bp region from a sample of genome‐wide SNPs, yielding a dataset of 20,639,768 SNPs in total. We first created a global estimation matrix for f2‐statistics. This matrix was then used to fit the model with different admixture edges under the species tree. Based on the score and worst residual, different admixture edges and models were compared. We utilized qpAdm in ADMIXTOOLS2 to model the admixture events in the *XJ* with two and three ancestry sources. QpAdm used a set of source populations (left populations) and a set of reference populations (right populations) to model the ancestry of target population, without the need for an explicit description of phylogenetic relationships. Our base set of reference populations had different relationships with the target populations, excluding the Xinjiang black honeybee, *A. m. ligustica, A. m. sinisxinyuan*, *A. m. pomonella*, and *A. m. carnica* populations. Our source populations included the Xinjiang black honeybee, *A. m.ligustica, A. m. sinisxinyuan*, *A. m. pomonella*, *A. m. carnica*, and three potential sympatric groups. We adopted a general method for modeling admixture.

Graphs using qpGraph in ADMIXTOOLS started with a basic NJ tree, and then we added the extra admixture (Table S7). An optimal tree model was constructed based on the observed f‐statistics f_2_ for all possible pairs of populations. A |Z‐score| < 3 between the observed and expected values (determined by the block jackknife) was required.

### 4.8 Genomic Signatures of Natural Selection and Adaptation in *A. mellifera*

The two‐layer admixture model implemented in efficient local ancestry inference (Van der Auwera et al. 2013) was employed to infer introgressed segments in the *XJ* (the admixed population, −p 1) with *A. m. sinisxinyuan* as the source population 2 (−p 11) and *A. m. carnica* as the source population 1 (−p 10). We conducted 30 iterations with mixture generation values of 100, 200, and 300, and the averaged results for the three independent runs were used for detecting introgression tracts. We summed the proportion of ancestors from source population 2 ancestry in each 10 kb window, which is consistent with *F*_*d*_, and the top 1% windows were defined as putative introgression domains.

We identified regions in *A. mellifera* that are putatively introgressed (Martin et al. 2015). We calculated the *F*_*d*_ value for each 10‐kb window across the entire genome of D (M1, M2; M3, *A. cerana* ). This value was influenced by the number of informative sites within these windows. Populations M1, M2, and M3 corresponded to *A. m. carnica*, Xinjiang black honeybee, and *A. m. sinisxinyuan*, respectively. Divergence (*Dxy*) was computed for each window between populations M2 and M3 for each comparison in the *D* statistic analysis. Because *Dxy* quantified sequence divergence, introgressed windows typically exhibited lower *D*xy values compared to non‐introgressed background windows. We used a cutoff of *p* < 0.05 for the *F*_d_ value to distinguish between introgressed and background windows (Table S9). Hits identified by one of statistics were considered candidate regions with signatures of introgression (Table S10). Genes within 1 kb flanking regions of candidate regions were regarded as candidate genes under introgression. ELAI was run for 30 iterations with 20 EM steps; we used a generation time of 1 year and a recombination rate of 1 cM/Mb; the burn‐in was set to 10 generations.

### 4.9 Gene Ontology (GO) Enrichment and KEGG Analysis

We implemented GO enrichment analysis and KEGG pathways analyses for putative genes on the datasets of 550 genes (Tables S11 and S12) using R package clusterProfiler v3.16100 (Yu et al. 2012).

### 4.10 Transcriptomic Expression Analysis of Introgressed Top Genes

To investigate the functional implications of adaptive introgression, we performed transcriptomic expression analysis on genes harboring top signatures of introgression (defined as those within the top 1% of D‐statistics or f4‐ratio values). Transcriptomic data were obtained from population‐wide RNA‐sequencing of 15 individuals (5*XJ* vs. 10*A. m. sinisxinyuan*) representing distinct provenances as described in Table 2. Raw sequencing reads were quality‐filtered using Trimmomatic v0.39 (Bolger et al. 2014) The cleaned reads were then aligned to the *A. mellifera* reference genome (Amel_HAv3.1) using STAR v2.7.10b (Dobin et al. 2013). Alignment was performed with the following parameters: ‐runThreadN 10 ‐outSAMtype BAM SortedByCoordinate ‐outFilterMultimapNmax 20 ‐alignSJoverhangMin 8 ‐alignSJDBoverhangMin 1 ‐outFilterMismatchNmax 999 ‐outFilterMismatchNoverReadLmax 0.04 ‐alignIntronMin 20 ‐alignIntronMax 1,000,000 ‐alignMatesGapMax 1,000,000. Gene expression quantification was conducted using RSEM v1.3.3 with default parameters to generate expected counts and FPKM values. Differential expression analysis was performed using FPKM values: genes were filtered to retain those with FPKM > 1 in at least 50% of samples in either group. Statistical significance was assessed using *t*‐test, and the false discovery rate (FDR) was controlled using the Benjamini‐Hochberg correction. Genes with FDR < 0.05 and absolute log2 fold change > 1 were considered significantly differentially expressed.

**TABLE 2:** Information on the 15 samples (Xinjiang black honeybee VS *A. m. sinisxinyuan*) for the transcriptomic analysis of honeybees from the Tianshan Mountains region.

	Sample name	Longitude	Latitude	Country	Date
1	*XJ*	83.473	43.732	Nilek	2025.8.20
2	*XJ*	83.393	43.758	Nilek	2025.8.20
3	*XJ*	83.336	43.759	Nilek	2025.8.20
4	*XJ*	83.515	43.752	Nilek	2025.7.2
5	*XJ*	83.693	43.708	Nilek	2025.7.3
6	*A. m. sinisxinyuan*	83.322	43.474	Xinyuan	2025.8.21
7	*A. m. sinisxinyuan*	83.928	43.329	Xinyuan	2025.8.22
8	*A. m. sinisxinyuan*	83.928	43.329	Xinyuan	2025.8.22
9	*A. m. sinisxinyuan*	83.928	43.329	Xinyuan	2025.8.22
10	*A. m. sinisxinyuan*	83.328	43.644	Xinyuan	2025.8.21
11	*A. m. sinisxinyuan*	83.328	43.644	Xinyuan	2025.8.21
12	*A. m. sinisxinyuan*	83.328	43.644	Xinyuan	2025.8.21
13	*A. m. sinisxinyuan*	83.328	43.644	Xinyuan	2025.8.21
14	*A. m. sinisxinyuan*	83.328	43.644	Xinyuan	2025.8.21
15	*A. m. sinisxinyuan*	83.328	43.644	Xinyuan	2025.8.21

## Funding

National Natural Science Foundation of China (32260124); Natural Science Foundation of Xinjiang Uygur Autonomous (2021D01B100); and Third Xinjiang Integrated Scientific Expedition (2021xjkk0503).

## Conflicts of Interest

The authors declare no conflicts of interest.

## Supporting information


**Figure S1:** Geographic distribution of western honeybee ( *Apis mellifera* ) subspecies in the Tianshan Mountains. The map shows locations for three key populations: *A. m. sinisxinyuan* (yellow triangles), *A. m. pomonella* (blue squares), and Xinjiang black honeybee (*XJ*, red circles). Sampling sites are overlaid on a topographic base map with elevation gradients and major geographic features, including national boundaries (red lines), prefecture‐level city boundaries (gray lines), and rivers (light blue lines). The inset map of Xinjiang highlights the Tianshan Mountains region (black box) relative to the broader study area.
**Figure S2:** Morphological characteristics of the three castes of Xinjiang black honeybee (*XJ*). The image displays a queen (♀, top), drone (♂, bottom left), and worker (bottom right), each labeled with their respective caste and sex symbols. The queen is marked with a red identification tag (number 19) on the thorax.
**Figure S3:** Boundary marker for the National Xinjiang Black Honeybee Genetic Resources Protection Area in the Ili River Valley, Xinjiang. The stone marker, inscribed with both Chinese and English, denotes the official designation of this conservation site by the Xinjiang Uygur Autonomous Region People's Government in May 2020.
**Figure S4:** Kinship coefficient estimation for *Apis mellifera* individuals using the KING‐Robust method. The plot displays the relationship between the proportion of zero‐identical‐by‐state (Zero IBS) genotypes and the estimated kinship coefficient. Dashed horizontal lines indicate kinship thresholds for different degrees of relatedness: duplicate or monozygotic (MZ) twins (top), first‐degree relatives (1st‐degree), second‐degree relatives (2nd‐degree), and fourth‐degree relatives (4th‐degree). The color‐coded points represent individuals from each lineage, with *XJ* (pink) and other lineages (e.g., A: teal, U: light blue, L: orange, M: purple, O: gray, C: green) showing distinct clustering patterns. The analysis excluded first‐degree relatives and monozygotic (MZ) duplicates, which are detailed in Table S3. This analysis was performed to identify and exclude closely related individuals (e.g., duplicates, MZ twins) and ensure the independence of samples in downstream population genomic analyses.
**Figure S5:** Cross‐validation (CV) error for different number of K in the ADMIXTURE analysis. Minimum of estimated CV error on *K* = 6 suggests the most suitable number of clusters.
**Figure S6:** Value of θ_Π_, *F*st for Xinjiang black honeybees (XJ) and the *A. mellifera* lineages. Each circle represents a regional lineage, the size of the circle represents the genetic diversity within the population, and the value in the circle is θ_Π_. The values in the circles are quantitative indicators of genetic diversity, the values on the lines indicate the value of *F*st between the two populations.
**Figure S7:** Genomic inbreeding and homozygous‐by‐descent (HBD) segment analysis across *Apis mellifera* lineages. (A) Genomic inbreeding coefficient (F_G‐T) as a function of the threshold (T) used to define HBD segments, showing lineage‐specific trends (e.g., *XJ* in pink, lineage A in teal). (B) Proportion of the genome covered by HBD segments of varying lengths (in centimorgans, cM), with lineages distinguished by color. (C‐D) Proportion of the genome in each HBD class (defined by rate R_k) for different lineages, illustrating variation in inbreeding patterns. XJ exhibits higher inbreeding than lineage C at 1024 generation.
**Figure S8:** Model selection for migration edges in TreeMix analysis of *Apis mellifera* lineages. (Top) Mean log‐likelihood (± standard deviation) and proportion of variance explained across different numbers of migration edges (m). (Bottom) Δm (change in log‐likelihood) as a function of m, showing a sharp increase at m = 2 (indicating a major improvement in model fit) and minor gains at higher m. These plots guide the selection of migration edges to infer historical gene flow events, with m = 2 chosen to balance model fit and biological interpretability.
**Figure S9:** *f*_3_ statistics, *f*_4‐ratio,Dsuit_ for Xinjiang black honeybees (XJ) with *Apis mellifera* population levels. (A) *f*_4‐ratio_, (*f*_4_(*XJ*, *A. mellifera* population; X, Y)) to detect asymmetric introgression, where red indicates significant positive values (potential gene flow between X and XJ). (B) D‐statistics for testing introgression, with points above the significance threshold indicating significant gene flow. These analyses collectively identify lineage‐specific introgression events, including contributions from *A. m. sinisxinyuan* (M lineage) and *A. m. pomonella* (O lineage) to XJ. *f*_3_ statistics calculated in the form (XJ; X; *Apis mellifera* population), a lower *f*3‐statistic means greater gene flow. Introgression from *A. m. ligustica* (C lineage) to XJ was detected.
**Figure S10:** qpGraph depicting genetic relationships among *Apis mellifera* subspecies/populations inferred using ADMIXTOOLS. The analysis was initiated with a basic tree topology determined by a whole‐genomic SNP maximum likelihood (ML) tree. Branch labels indicate divergence nodes (numbers) and admixture proportions (percentages). Different colored branches represent distinct evolutionary lineages, with the dotted line highlighting a 17%/83% admixture event between *A. m. sinisxinyuan*, and the Xinjiang black honeybee population.
**Table S1:** Information on genome data and mapping statistics.
**Table S2:** Summary of published *Apis mellifera* samples used in this study, with color‐coded exclusion criteria for downstream analysis.
**Table S3:** The relationship of each sample in each population inferred by the King program. ID1: The first individual of the pair; ID2: The second individual of the pair; N_SNP: The number of SNPS that do not have missing SNPS in either of the individuals; HetHet: Percentage of SNPs with double heterozygotes; IBS0: Proportion of SNPs with 0‐IBS (identical‐by‐state); Kinship: Kinship coefficient estimated by the program.
**Table S4:** Excluded *Apis mellifera* samples and reasons for exclusion in downstream analysis.
**Table S5:** Cross‐validation (CV) error for varying values of K in the ADMIXTURE analysis.
**Table S6:** Pairwise FST values between Xinjiang black honeybee (XJ) and other *Apis mellifera* subspecies.
**Table S7:** D‐statistics tests D for all pair of *Apis mellifera* populations. D‐statistics were implemented to detect admixture between X and Z (in parentheses) only showing|Z‐scores| ≥ 3 groups.
**Table S8:** qpAdm admixture modeling results for Xinjiang black honeybee (XJ) with different source populations.
**Table S9:** Introgressed genes (NCBI accession numbers) were identified using at least one statistical test (Fd, Dxy, or ELAI) and used for GO enrichment analysis.
**Table S10:** Top enriched GO terms among candidate introgressed regions (Biological Process, Molecular Function, Cellular Component).
**Table S11:** Summary gene annotation of candidate genes under putative introgression.
**Table S12:** Kyoto Encyclopedia of Genes and Genomes (KEGG) pathway enrichment analysis of putatively introgressed genes.

## Data Availability

The raw sequencing reads were deposited in the National Center for Biotechnology Information database under the BioProject accession code PRJNA1040327.
