# Supplementary material for: Population Genomics Reveals Genetic Diversity, Introgression, and Genetic Differentiation in Tianshan Mountains Western Honeybees ( Apis mellifera )
Source: Evol Appl. 2026 May 6;19(5):e70248. doi: 10.1111/eva.70248 (PMC13148138; doi:10.1111/eva.70248)
Supplement: Supplementary file 1 — Figure S1: Geographic distribution of western honeybee ( Apis mellifera ) subspecies in the Tianshan Mountains. The map shows locations for three key populations: A. m. sinisxinyuan (yellow triangles), A. m. pomonella (blue squares), and Xinjiang black honeybee (XJ, red circles). Sampling sites are overlaid on a topographic base map with elevation gradients and major geographic features, including national boundaries (red lines), prefecture‐level city boundaries (gray lines), and rivers (light blue lines). The inset map of Xinjiang highlights the Tianshan Mountains region (black box) relative to the broader study area. Figure S2: Morphological characteristics of the three castes of Xinjiang black honeybee (XJ). The image displays a queen (♀, top), drone (♂, bottom left), and worker (bottom right), each labeled with their respective caste and sex symbols. The queen is marked with a red identification tag (number 19) on the thorax. Figure S3: Boundary marker for the National Xinjiang Black Honeybee Genetic Resources Protection Area in the Ili River Valley, Xinjiang. The stone marker, inscribed with both Chinese and English, denotes the official designation of this conservation site by the Xinjiang Uygur Autonomous Region People's Government in May 2020. Figure S4: Kinship coefficient estimation for Apis mellifera individuals using the KING‐Robust method. The plot displays the relationship between the proportion of zero‐identical‐by‐state (Zero IBS) genotypes and the estimated kinship coefficient. Dashed horizontal lines indicate kinship thresholds for different degrees of relatedness: duplicate or monozygotic (MZ) twins (top), first‐degree relatives (1st‐degree), second‐degree relatives (2nd‐degree), and fourth‐degree relatives (4th‐degree). The color‐coded points represent individuals from each lineage, with XJ (pink) and other lineages (e.g., A: teal, U: light blue, L: orange, M: purple, O: gray, C: green) showing distinct clustering patterns. The analysis excluded [file EVA-19-e70248-s001.doc]

Supplementary materials for

**Population genomics reveals genetic diversity, introgression, and genetic differentiation in Tianshan Mountain western honeybees (*Apis mellifera*)**

**This PDF file includes:**

Text

Figs. S1 to S10

**Other Materials for this manuscript include the following:**

Tables S1 to S12

**Supplementary**

**Fig. S1| Geographic distribution of western honeybee (*Apis mellifera*) subspecies in the Tianshan Mountains.** The map shows locations for three key populations: *A. m. sinisxinyuan* (yellow triangles), *A. m. pomonella* (blue squares), and Xinjiang black honeybee (*XJ*, red circles). Sampling sites are overlaid on a topographic base map with elevation gradients and major geographic features, including national boundaries (red lines), prefecture-level city boundaries (gray lines), and rivers (light blue lines). The inset map of Xinjiang highlights the Tianshan Mountains region (black box) relative to the broader study area.

**Fig. S2| Morphological characteristics of the three castes of Xinjiang black honeybee ( *XJ*).** The image displays a queen (♀, top), drone (♂, bottom left), and worker (bottom right), each labeled with their respective caste and sex symbols. The queen is marked with a red identification tag (number 19) on the thorax**.**

**Fig. S3| Boundary marker for the National Xinjiang Black Honeybee Genetic Resources Protection Area in the Ili River Valley, Xinjiang.** The stone marker, inscribed with both Chinese and English, denotes the official designation of this conservation site by the Xinjiang Uygur Autonomous Region People’s Government in May 2020 .

**Fig. S4**| **Kinship coefficient estimation for *Apis mellifera* individuals using the KING-Robust method.** The plot displays the relationship between the proportion of zero-identical-by-state (Zero IBS) genotypes and the estimated kinship coefficient. Dashed horizontal lines indicate kinship thresholds for different degrees of relatedness: duplicate or monozygotic (MZ) twins (top), first-degree relatives (1st-degree), second-degree relatives (2nd-degree), and fourth-degree relatives (4th-degree). The color-coded points represent individuals from each lineage, with *XJ* (pink) and other lineages (e.g., A: teal, U: light blue, L: orange, M: purple, O: gray, C: green) showing distinct clustering patterns. The analysis excluded first-degree relatives and monozygotic (MZ) duplicates, which are detailed in Table S3. This analysis was performed to identify and exclude closely related individuals (e.g., duplicates, MZ twins) and ensure the independence of samples in downstream population genomic analyses.

**Fig. S5| Cross-validation (CV) error for different number of K in the ADMIXTURE analysis.** Minimum of estimated CV error on K= 6 suggests the most suitable number of clusters**.**

**Fig. S6| Value of θ_Π_, *F*st for Xinjiang black honeybees (XJ) and the *A. mellifera* lineages**. Each circle represents a regional lineage, the size of the circle represents the genetic diversity within the population, and the value in the circle is θ_Π_. The values in the circles are quantitative indicators of genetic diversity, The values on the lines indicate the value of *F*st between the two populations.

**Fig. S7| Genomic inbreeding and homozygous-by-descent (HBD) segment analysis across *Apis mellifera* lineages.** (A) Genomic inbreeding coefficient (F_G-T) as a function of the threshold (T) used to define HBD segments, showing lineage-specific trends (e.g., *XJ* in pink, lineage A in teal). (B) Proportion of the genome covered by HBD segments of varying lengths (in centimorgans, cM), with lineages distinguished by color. (C-D) Proportion of the genome in each HBD class (defined by rate R_k) for different lineages, illustrating variation in inbreeding patterns. XJ exhibits higher inbreeding than lineage C at 1024 generation.

**Fig. S8| Model selection for migration edges in TreeMix analysis of *Apis mellifera* lineages.** (Top) Mean log-likelihood (± standard deviation) and proportion of variance explained across different numbers of migration edges (m). (Bottom) Δm (change in log-likelihood) as a function of m, showing a sharp increase at m=2 (indicating a major improvement in model fit) and minor gains at higher m. These plots guide the selection of migration edges to infer historical gene flow events, with m=2 chosen to balance model fit and biological interpretability.

**Fig. S9| *f*_3_ statistics ,** ***f*_4-ratio, Dsuit_ for** **Xinjiang black honeybees (XJ) with *Apis mellifera* population levels .** (A) ***f*_4-ratio_,** (*f*_4_(*XJ*, *A. mellifera* population; X, Y)) to detect asymmetric introgression, where red indicates significant positive values (potential gene flow between X and XJ). (B) D-statistics for testing introgression, with points above the significance threshold indicating significant gene flow. These analyses collectively identify lineage-specific introgression events, including contributions from *A. m. sinisxinyuan* (M lineage) and *A. m. pomonella* (O lineage) to XJ. *f*_3_ statistics calculated in the form (**XJ; X;** *Apis mellifera* population), a lower *f*3-statistic means greater gene flow. Introgression from *A. m. ligustica* (C lineage) to XJ was detected.

**Fig. S10| qpGraph depicting genetic relationships among *Apis mellifera* subspecies/populations inferred using ADMIXTOOLS**. The analysis was initiated with a basic tree topology determined by a whole-genomic SNP maximum likelihood (ML) tree. Branch labels indicate divergence nodes (numbers) and admixture proportions (percentages). Different colored branches represent distinct evolutionary lineages, with the dotted line highlighting a 17%/83% admixture event between *A. m. sinisxinyuan*, and the Xinjiang black honeybee population.

**Table S1 Information on genome data and mapping statistics.**

**Table S2 Summary of published *Apis mellifera* samples used in this study, with color-coded exclusion criteria for downstream analysis.**

**Table S3 The relationship of each sample in each population inferred by the King program. ID1: The first individual of the pair; ID2: The second individual of the pair; N_SNP: The number of SNPS that do not have missing SNPS in either of the individuals; HetHet: Percentage of SNPs with double heterozygotes; IBS0: Proportion of SNPs with 0-IBS (identical-by-state); Kinship: Kinship coefficient estimated by the program.**

**Table S4 Excluded *Apis mellifera* samples and reasons for exclusion in downstream analysis.**

**Table S5 Cross-validation (CV) error for varying values of K in the ADMIXTURE analysis**

**Table S6 Pairwise FST values between Xinjiang black honeybee (XJ) and other Apis mellifera subspecies.**

**Table S7 D-statistics tests D for all pair of Apis mellifera populations. D-statistics were implemented to detect admixture between X and Z (in parentheses) only showing |Z-scores| ≥ 3 groups.**

**Table S8 qpAdm admixture modeling results for Xinjiang black honeybee (XJ) with different source populations.**

**Table S9 Introgressed genes (NCBI accession numbers) were identified using at least one statistical test (Fd, Dxy, or ELAI) and used for GO enrichment analysis.**

**Table S10 Top enriched GO terms among candidate introgressed regions (Biological Process, Molecular Function, Cellular Component)**

**Table S11 Summary gene annotation of candidate genes under putative introgression**

**Table S12 Kyoto Encyclopedia of Genes and Genomes (KEGG) pathway enrichment analysis of putatively introgressed genes**


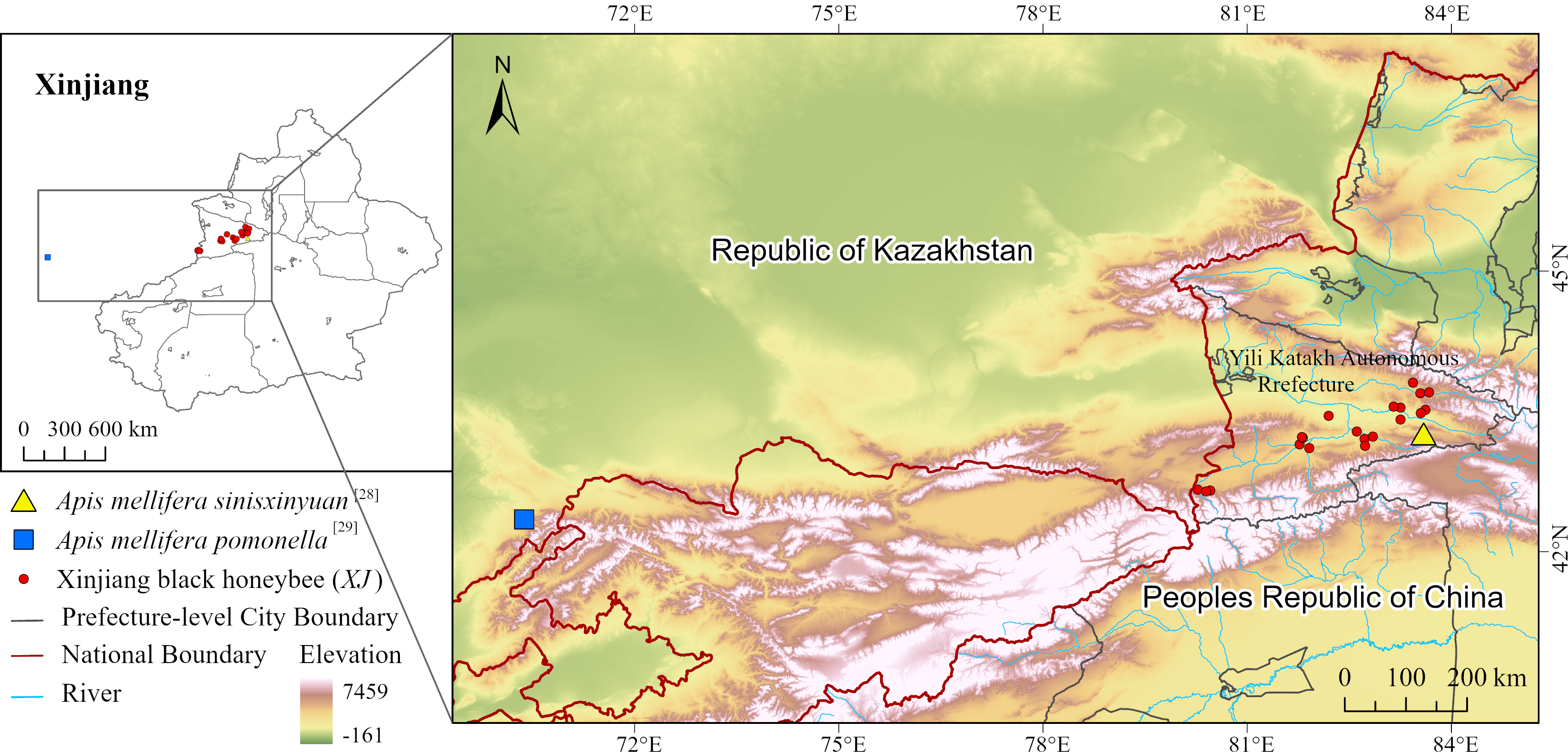


**Fig. S1**


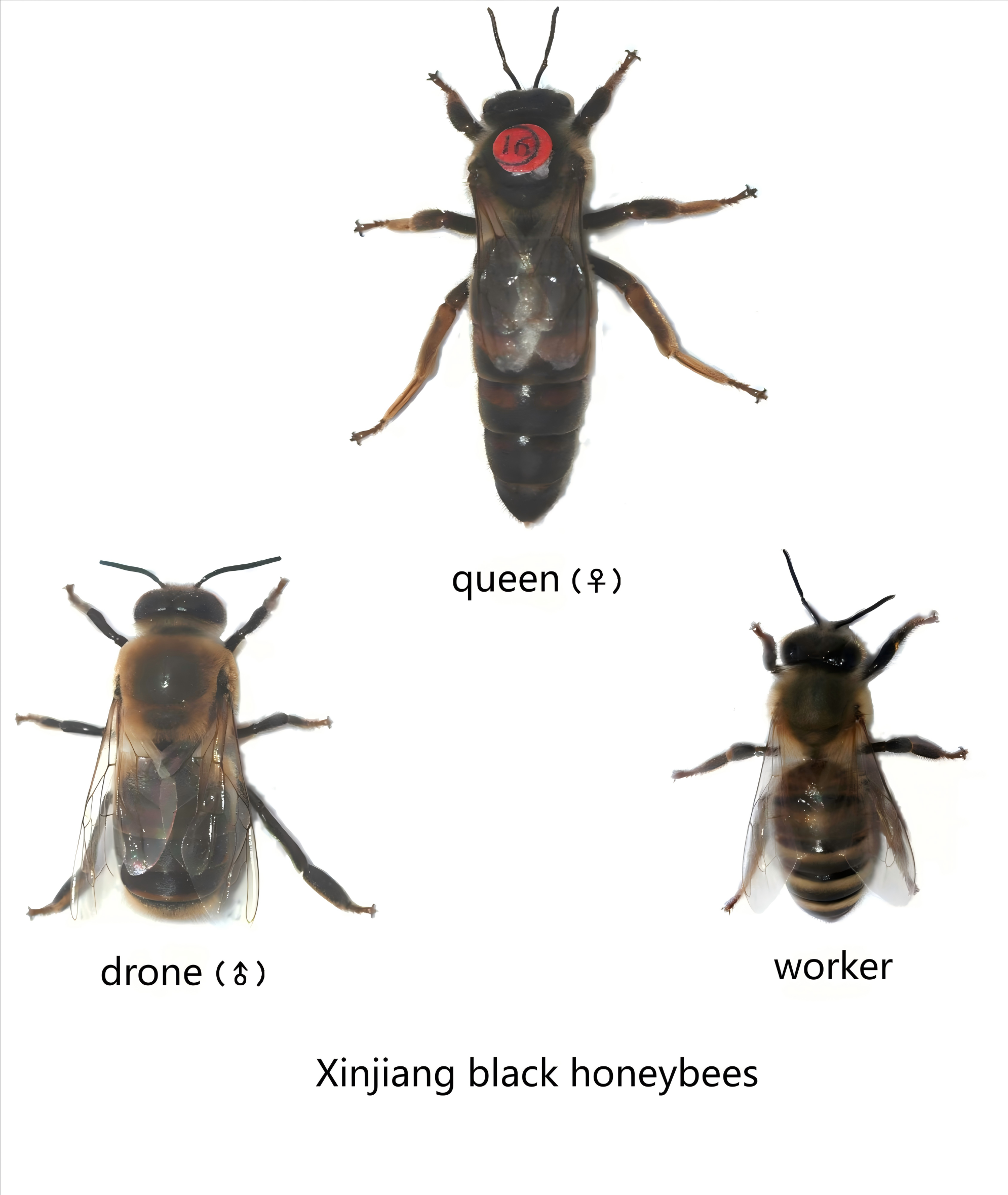


**Fig. S2**


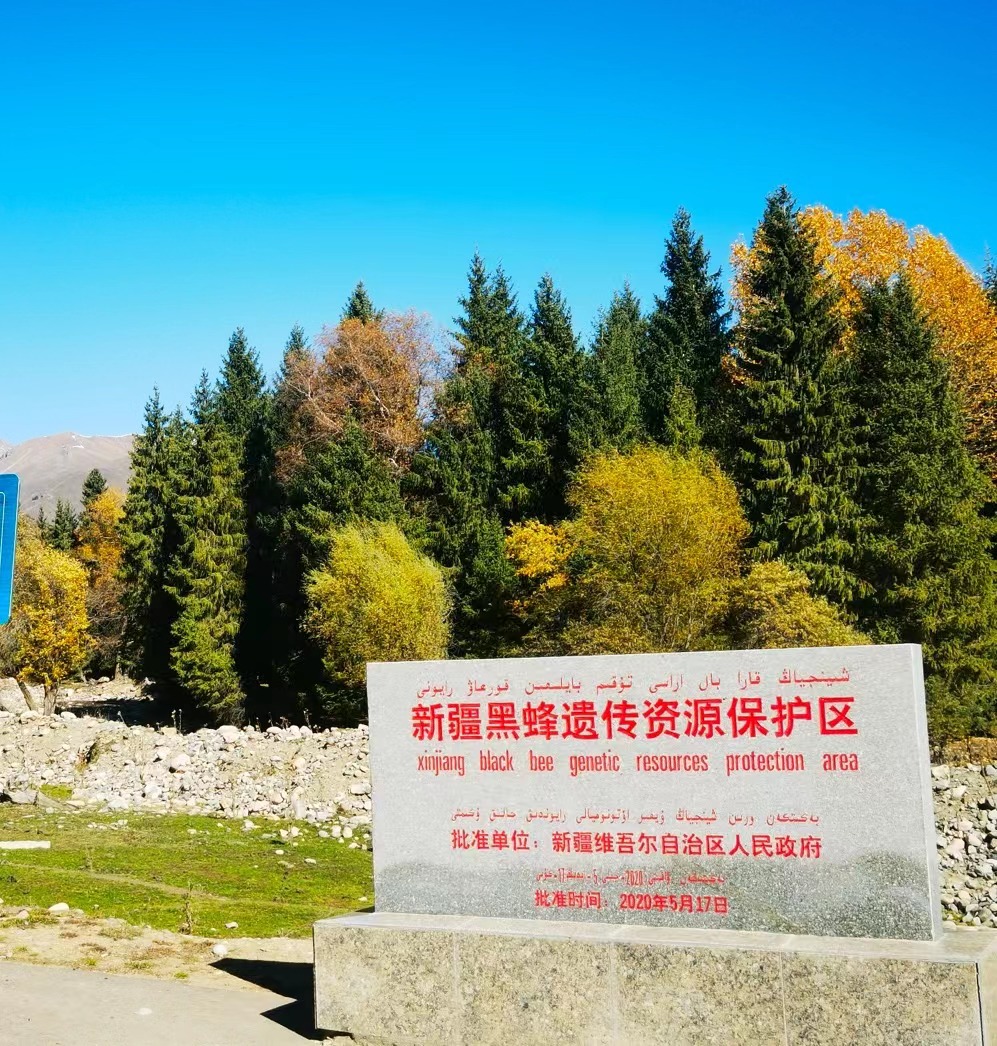


**Fig. S3**


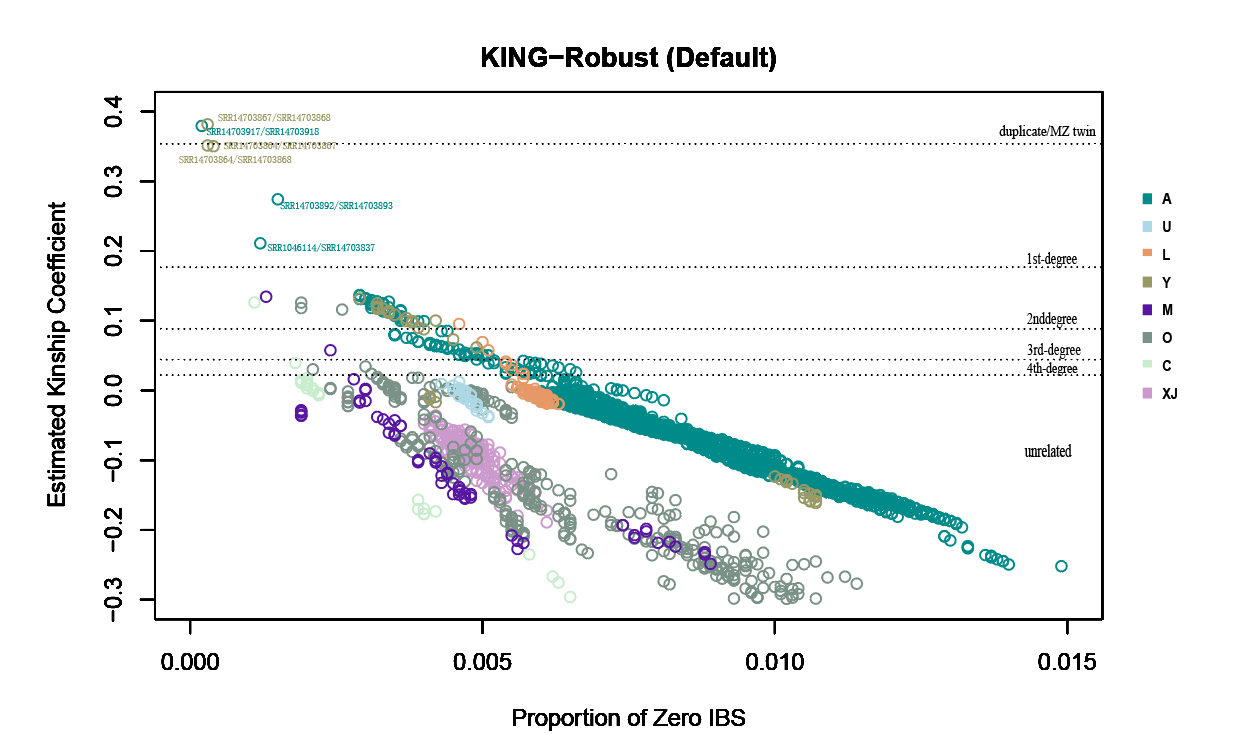


**Fig. S4**


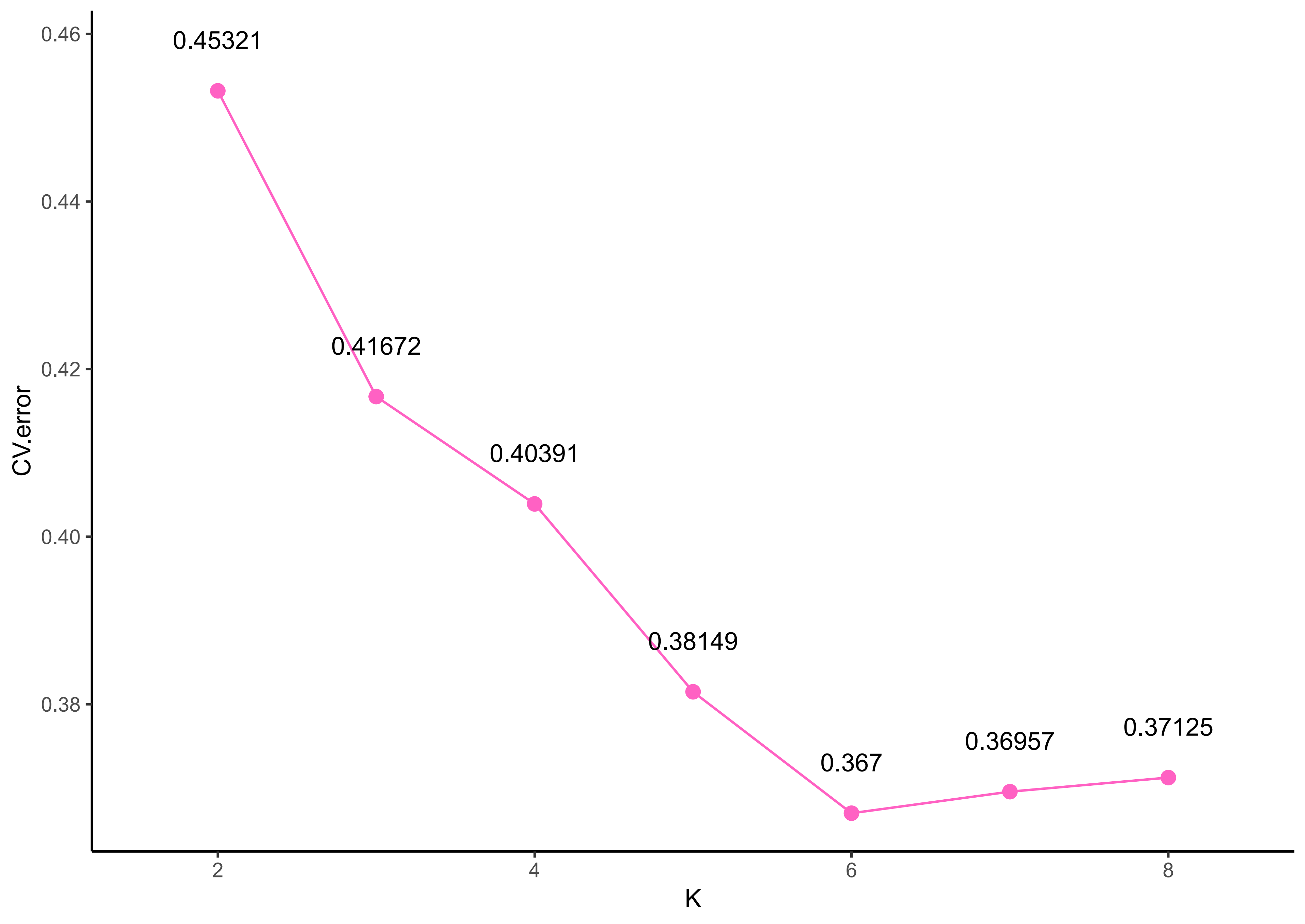


**Fig. S5.**

**
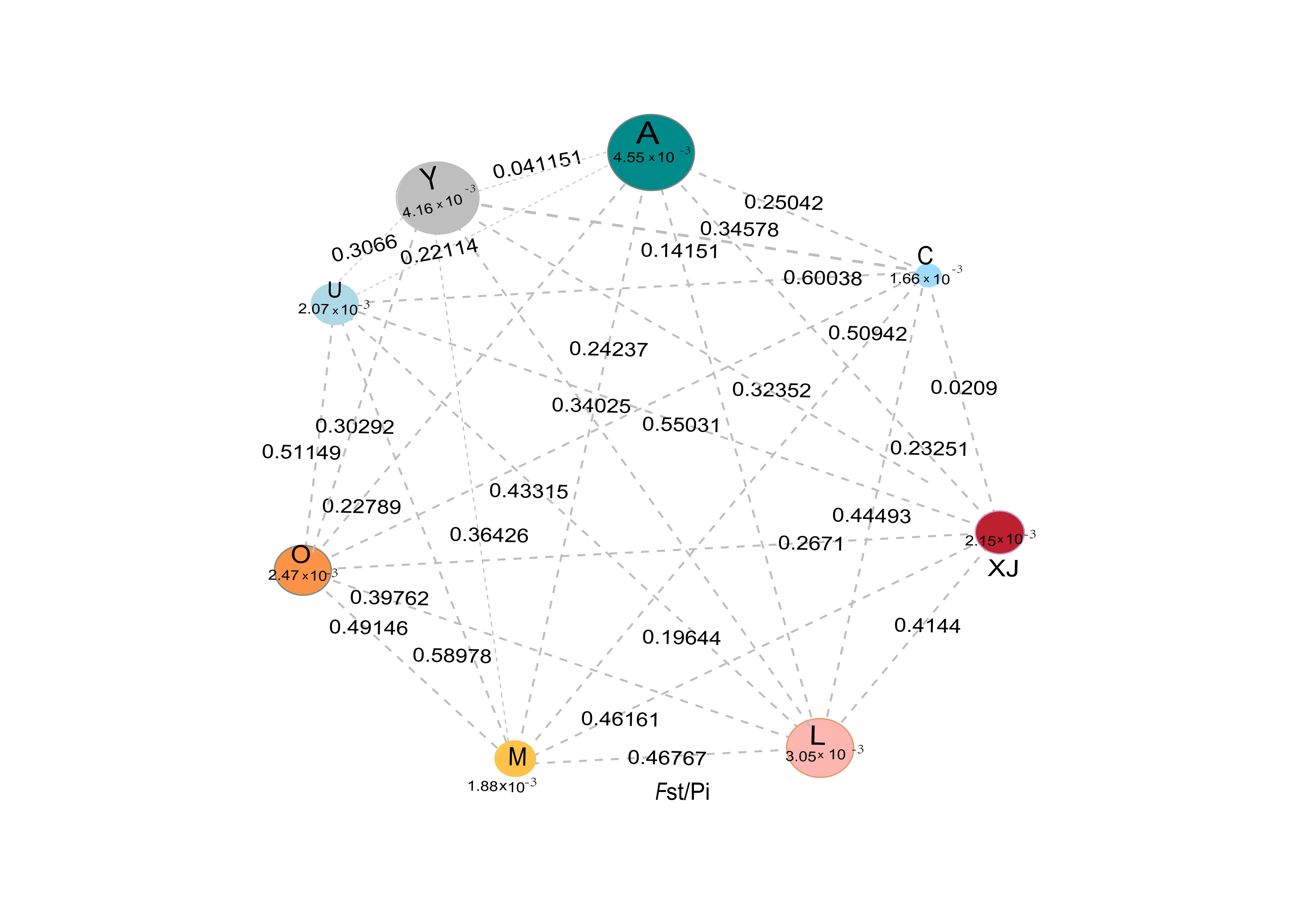
**

**Fig. S6**


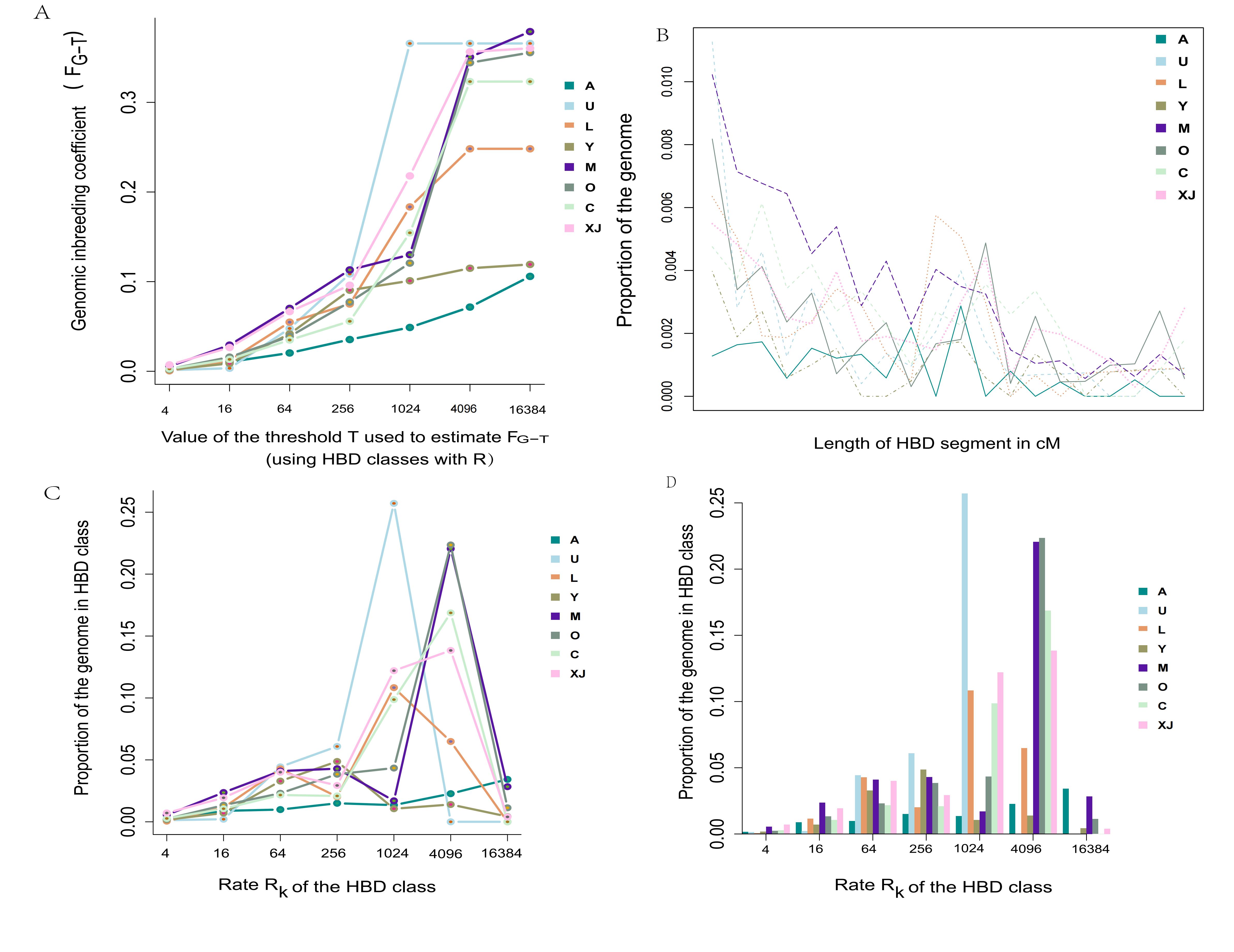


**Fig. S7**

**
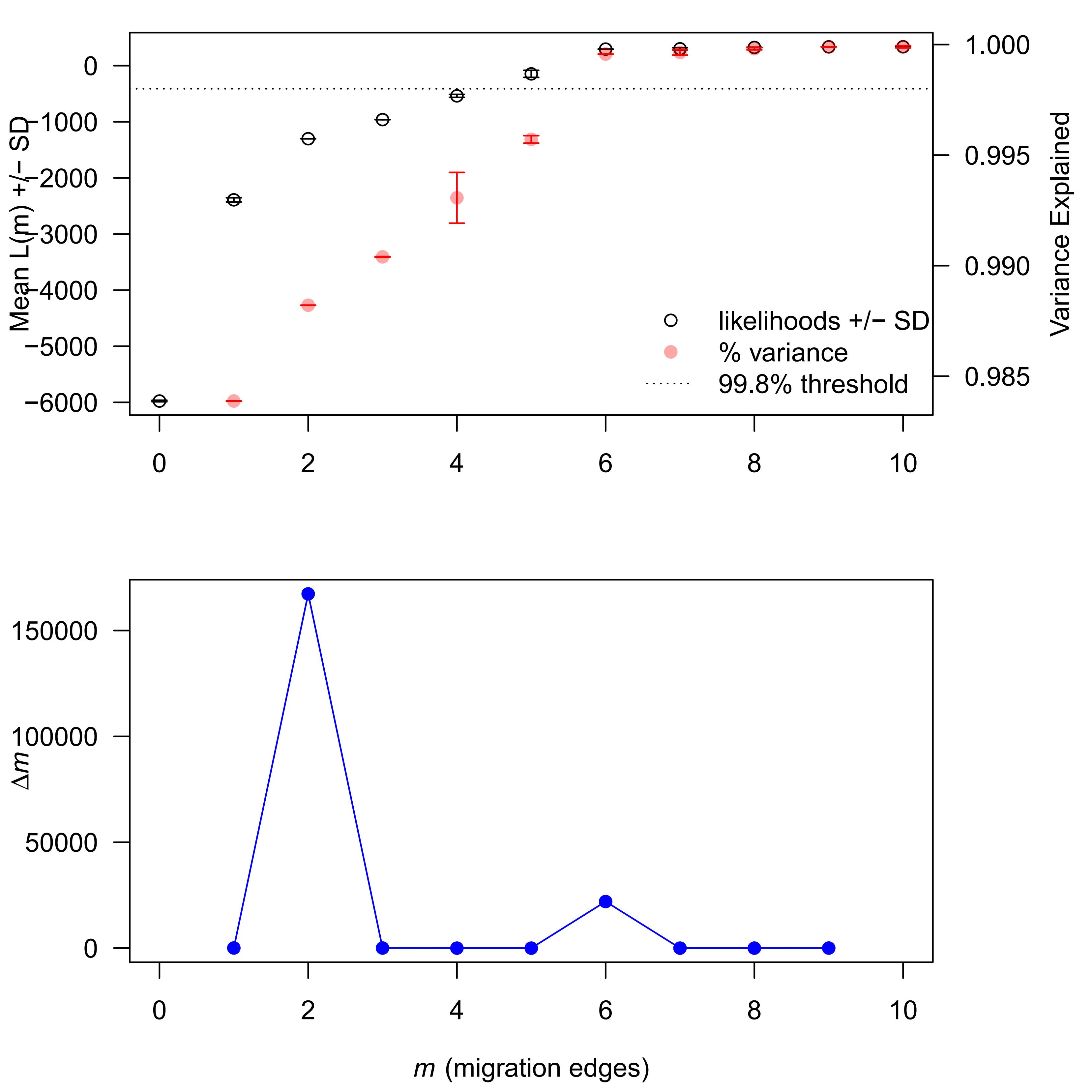
**

**Fig. S8**


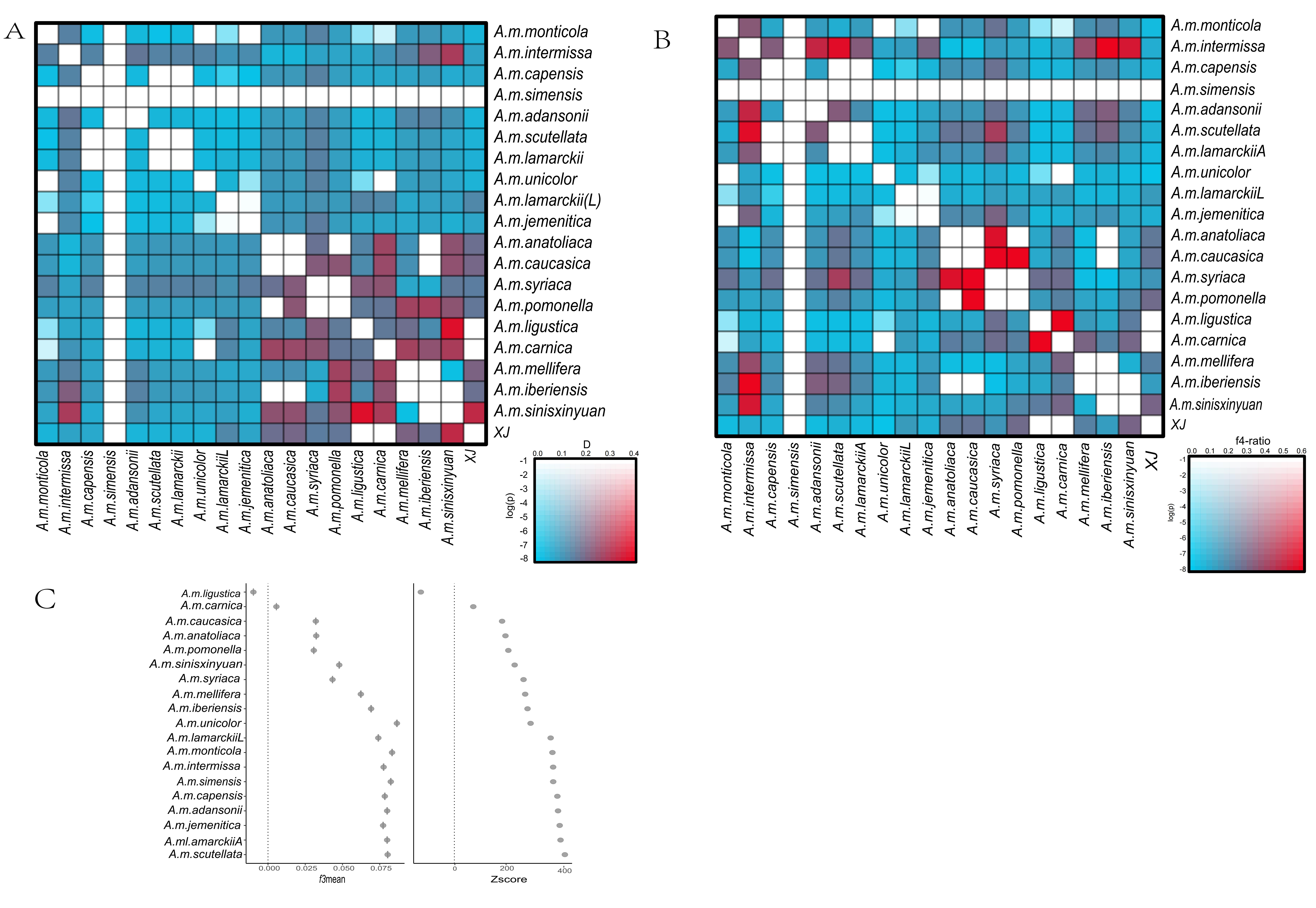


**Fig. S9**

**
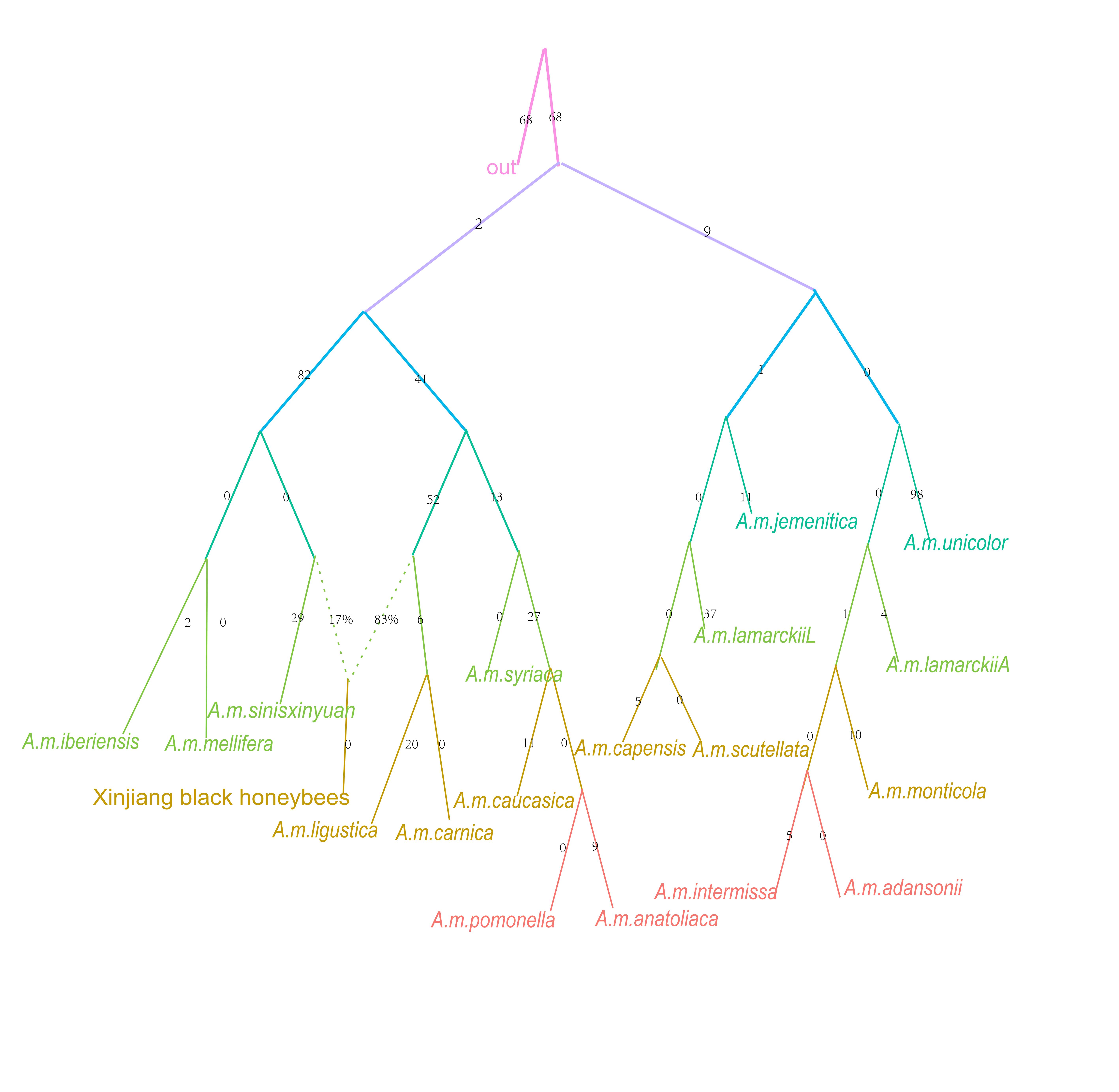
 Fig. S10**

| **Table S1. Information on genome data and mapping statistics.** | | | | | |
| --- | --- | --- | --- | --- | --- |
| **Sample ID** | **Clean data (bp)** | **Mapped data (bp)** | **Mapped (%)** | **Depth (X)** | **Coverage (%)** |
| Xinjiang black bee1 | 23643760 | 23500050 | 99. 39% | 11.47 | 97. 09% |
| Xinjiang black bee2 | 25020274 | 24865300 | 99. 38% | 12.57 | 97. 14% |
| Xinjiang black bee3 | 23912758 | 23768640 | 99. 40% | 12.14 | 96. 08% |
| Xinjiang black bee4 | 24834738 | 24707254 | 99. 49% | 11.96 | 97. 11 |
| Xinjiang black bee5 | 21881110 | 21761764 | 99. 45% | 11.09 | 97. 21% |
| Xinjiang black bee6 | 21801830 | 21608074 | 99. 11% | 9.95 | 97. 03% |
| Xinjiang black bee7 | 25112232 | 24968605 | 99. 43% | 12.27 | 97. 13% |
| Xinjiang black bee8 | 25576380 | 25458172 | 99. 54% | 12.28 | 97. 19% |
| Xinjiang black bee9 | 25178586 | 25040206 | 99. 45% | 11.63 | 97. 08% |
| Xinjiang black bee10 | 23125460 | 23006190 | 99. 48% | 11.7 | 97. 12 |
| Xinjiang black bee11 | 24971410 | 24812330 | 99. 36% | 12.72 | 97. 12 |
| Xinjiang black bee12 | 24431942 | 24249288 | 99. 25% | 12.54 | 97. 17% |
| Xinjiang black bee13 | 25619910 | 25490103 | 99. 49% | 12.73 | 97. 24% |
| Xinjiang black bee14 | 26409870 | 26265202 | 99. 45% | 13.31 | 97. 25 |
| Xinjiang black bee15 | 26376348 | 26256378 | 99. 55% | 10.98 | 97. 08 |
| Xinjiang black bee16 | 25651210 | 25523051 | 99. 50% | 12.71 | 97. 28% |
| Xinjiang black bee17 | 25356826 | 25230990 | 99. 50% | 13.1 | 97. 19% |
| Xinjiang black bee18 | 24801000 | 24517111 | 98. 86% | 9.24 | 96. 88% |
| Xinjiang black bee19 | 24400778 | 24234596 | 99. 32% | 12.28 | 97. 21 |
|  |  |  |  |  |  |
| Coverage (%) = Covered length/Genome size x 100  Mapped (%) = Mapped bases/Clean data x 100  Depth (X) = Mapped bases/Covered length (bp) |  |  |  |  |  |

| **Table S2. Summary of published Apis mellifera samples used in this study, with color-coded exclusion criteria for downstream analysis.** | | | | | | | |  |  |  |  |
| --- | --- | --- | --- | --- | --- | --- | --- | --- | --- | --- | --- |
| **BioProject** | **Run** | ***Subspecies*** | **Lineage** | **geo_loc_name_country** | **geo_loc_name_country_continent** | **geo_loc_name** | **Latitude** | **Longitude** | **bee** | **LibraryLayout** | **Reason for exclusion** |
| PRJNA729035 | SSR1046114 | *A. m. intermissa* | A Lineage | Morocco | Africa | Morocco | -4.52899 | 39.153618 | worker | PAIRED | Pairs with a kinship coefficient > 0.177 (twins and first-degree relationship) were closely related individuals |
| PRJNA729035 | SSR14703777 | *A.m.anatoliaca* | O Lineage | Turkey | Asia | Turkey: Fethiye | 0.9541 | 34.604767 | worker | PAIRED |  |
| PRJNA729035 | SSR14703778 | *A.m.anatoliaca* | O Lineage | Turkey | Asia | Turkey: Fethiye | 0.9541 | 34.604767 | worker | PAIRED |  |
| PRJNA729035 | SSR14703779 | *A.m.anatoliaca* | O Lineage | Turkey | Asia | Turkey: Fethiye | 0.9541 | 34.604767 | worker | PAIRED |  |
| PRJNA729035 | SSR14703780 | *A.m.anatoliaca* | O Lineage | Turkey | Asia | Turkey: Fethiye | 0.9541 | 34.604767 | worker | PAIRED |  |
| PRJNA729035 | SSR14703781 | *A.m.anatoliaca* | O Lineage | Turkey | Asia | Turkey: Fethiye | 0.500832 | 36.54557 | worker | PAIRED |  |
| PRJNA729035 | SSR14703782 | *A.m.anatoliaca* | O Lineage | Turkey | Asia | Turkey: Menemen | 0.500832 | 36.54557 | worker | PAIRED |  |
| PRJNA729035 | SSR14703783 | *A.m.anatoliaca* | O Lineage | Turkey | Asia | Turkey: Menemen | 31.25263 | -6.130111 | worker | PAIRED |  |
| PRJNA729035 | SSR14703784 | *A.m.monticola* | A Lineage | Kenya | Africa | Kenya: Moorland at Mt. Elgon | 31.25263 | -6.130111 | worker | PAIRED |  |
| PRJNA729035 | SSR14703785 | *A.m.capensis* | A Lineage | South Africa | Africa | South Africa: Cape Point | 31.25263 | -6.130111 | worker | PAIRED | Ambiguous Subspecies Assingment/Recent Admixture |
| PRJNA729035 | SSR14703786 | *A.m.monticola* | A Lineage | Kenya | Africa | Kenya: Moorland at Mt. Elgon | 31.25263 | -6.130111 | worker | PAIRED |  |
| PRJNA729035 | SSR14703787 | *A.m.monticola* | A Lineage | Kenya | Africa | Kenya: Moorland at Mt. Elgon | 31.25263 | -6.130111 | worker | PAIRED |  |
| PRJNA729035 | SSR14703788 | *A.m.monticola* | A Lineage | Kenya | Africa | Kenya: Moorland at Mt. Elgon | 31.25263 | -6.130111 | worker | PAIRED |  |
| PRJNA729035 | SSR14703789 | *A.m.monticola* | A Lineage | Kenya | Africa | Kenya: Marchorwe | 31.52559 | -5.5428 | worker | PAIRED |  |
| PRJNA729035 | SSR14703790 | *A.m.monticola* | A Lineage | Kenya | Africa | Kenya: Marchorwe | 35.47001 | -6.029983 | worker | PAIRED |  |
| PRJNA729035 | SSR14703791 | *A.m.mellifera* | M Lineage | France | Europe | France: Landes | 35.47001 | -6.029983 | worker | PAIRED |  |
| PRJNA729035 | SSR14703792 | *A.m.mellifera* | M Lineage | France | Europe | France: Avignon | 35.47001 | -6.029983 | worker | PAIRED |  |
| PRJNA729035 | SSR14703793 | *A.m.mellifera* | M Lineage | France | Europe | France: Avignon | 35.47001 | -6.029983 | worker | PAIRED |  |
| PRJNA729035 | SSR14703794 | *A.m.mellifera* | M Lineage | France | Europe | France: Ille Sur Tet | 35.47001 | -6.029983 | worker | PAIRED |  |
| PRJNA729035 | SSR14703795 | *A.m.mellifera* | M Lineage | France | Europe | France: Ille Sur Tet | 35.47001 | -6.029983 | worker | PAIRED |  |
| PRJNA729035 | SSR14703796 | *A.m.adansonii* | A Lineage | Senegal | Africa | Senegal | 35.47001 | -6.029983 | worker | PAIRED |  |
| PRJNA729035 | SSR14703797 | *A.m.ligustica* | C Lineage | Italy | Europe | Italy: Bolongna | 35.47001 | -6.029983 | worker | PAIRED |  |
| PRJNA729035 | SSR14703798 | *A.m.ligustica* | C Lineage | Italy | Europe | Italy: Bolongna | -33.8 | 18.6 | worker | PAIRED |  |
| PRJNA729035 | SSR14703799 | *A.m.ligustica* | C Lineage | Italy | Europe | Italy: Milano | -33.8 | 18.6 | worker | PAIRED |  |
| PRJNA729035 | SSR14703800 | *A.m.ligustica* | C Lineage | Italy | Europe | Italy: Milano | -33.8 | 18.6 | worker | PAIRED |  |
| PRJNA729035 | SSR14703801 | *A.m.ligustica* | C Lineage | Italy | Europe | Italy: Milano | -33.8 | 18.6 | worker | PAIRED |  |
| PRJNA729035 | SSR14703802 | *A.m.ligustica* | C Lineage | Italy | Europe | Italy: Milano | -33.8 | 18.6 | worker | PAIRED |  |
| PRJNA729035 | SSR14703803 | *A.m.lamarckii* | A Lineage | Egypt | Africa | Egypt: Assiut | -33.8 | 18.6 | worker | PAIRED |  |
| PRJNA729035 | SSR14703804 | *A.m.lamarckii* | A Lineage | Egypt | Africa | Egypt: Assiut | -33.8 | 18.6 | worker | PAIRED |  |
| PRJNA729035 | SSR14703805 | *A.m.lamarckii* | A Lineage | Egypt | Africa | Egypt: Assiut | -33.8 | 18.6 | worker | PAIRED |  |
| PRJNA729035 | SSR14703806 | *A.m.lamarckii* | A Lineage | Egypt | Africa | Egypt: Assiut | -33.8 | 18.6 | worker | PAIRED |  |
| PRJNA729035 | SSR14703807 | *A.m.adansonii* | A Lineage | Burkina Faso | Africa | Burkina Faso: Kurkina | -33.8 | 18.6 | worker | PAIRED |  |
| PRJNA729035 | SSR14703808 | *A.m.lamarckii* | L Lineage | Egypt | Africa | Egypt: Assiut | -33.8 | 18.6 | worker | PAIRED |  |
| PRJNA729035 | SSR14703809 | *A.m.lamarckii* | L Lineage | Egypt | Africa | Egypt: Assiut | -33.8 | 18.6 | worker | PAIRED |  |
| PRJNA729035 | SSR14703810 | *A.m.lamarckii* | L Lineage | Egypt | Africa | Egypt: Assiut | 3.88965 | 40.2674 | worker | PAIRED |  |
| PRJNA729035 | SSR14703811 | *A.m.lamarckii* | L Lineage | Egypt | Africa | Egypt: Assiut | 3.88965 | 40.2674 | worker | PAIRED |  |
| PRJNA729035 | SSR14703812 | *A.m.lamarckii* | L Lineage | Kenya | Africa | Kenya: Mandera | -1.7493509 | 12.8162655 | worker | PAIRED |  |
| PRJNA729035 | SSR14703813 | *A.m.lamarckii* | L Lineage | Kenya | Africa | Kenya: Mandera | -1.7493509 | 12.8162655 | worker | PAIRED |  |
| PRJNA729035 | SSR14703814 | *A.m.lamarckii* | L Lineage | Kenya | Africa | Kenya: Mandera | -1.7493509 | 12.8162655 | worker | PAIRED |  |
| PRJNA729035 | SSR14703815 | *A.m.lamarckii* | L Lineage | Egypt | Africa | Egypt: Dairout | -1.7493509 | 12.8162655 | worker | PAIRED |  |
| PRJNA729035 | SSR14703816 | *A.m.lamarckii* | L Lineage | Egypt | Africa | Egypt: Dairout | -1.7493509 | 12.8162655 | worker | PAIRED |  |
| PRJNA729035 | SSR14703817 | *A.m.lamarckii* | L Lineage | Kenya | Africa | Kenya: Gede Ruins | -1.7493509 | 12.8162655 | worker | PAIRED |  |
| PRJNA729035 | SSR14703818 | *A.m.adansonii* | A Lineage | Burkina Faso | Africa | Burkina Faso: Kurkina | -1.7493509 | 12.8162655 | worker | PAIRED |  |
| PRJNA729035 | SSR14703819 | *A.m.lamarckii* | L Lineage | Egypt | Africa | Egypt: Dairout | -1.7493509 | 12.8162655 | worker | PAIRED |  |
| PRJNA729035 | SSR14703820 | *A.m.lamarckii* | L Lineage | Egypt | Africa | Egypt: Dairout | -29.7 | 31.1 | worker | PAIRED |  |
| PRJNA729035 | SSR14703821 | *A.m.lamarckii* | L Lineage | Kenya | Africa | Kenya: Gede Ruins | -29.7 | 31.1 | worker | PAIRED |  |
| PRJNA729035 | SSR14703822 | *A.m.lamarckii* | L Lineage | Egypt | Africa | Egypt: Dairout | -29.7 | 31.1 | worker | PAIRED |  |
| PRJNA729035 | SSR14703823 | *A.m.lamarckii* | L Lineage | Egypt | Africa | Egypt: Dairout | -29.7 | 31.1 | worker | PAIRED |  |
| PRJNA729035 | SSR14703824 | *A.m.lamarckii* | L Lineage | Kenya | Africa | Kenya: Gede Ruins | -17.7 | 31.1 | worker | PAIRED |  |
| PRJNA729035 | SSR14703825 | *A.m.intermissa* | A Lineage | Morocco | Africa | Morocco | -17.7 | 31.1 | worker | PAIRED |  |
| PRJNA729035 | SSR14703826 | *A.m.intermissa* | A Lineage | Morocco | Africa | Morocco | -17.7 | 31.1 | worker | PAIRED |  |
| PRJNA729035 | SSR14703827 | *A.m.intermissa* | A Lineage | Morocco | Africa | Morocco | 3.93675 | 41.868167 | worker | PAIRED |  |
| PRJNA729035 | SSR14703828 | *A.m.intermissa* | A Lineage | Morocco | Africa | Morocco | 3.93675 | 41.868167 | worker | PAIRED |  |
| PRJNA729035 | SSR14703829 | *A.m.adansonii* | A Lineage | Burkina Faso | Africa | Burkina Faso: Kurkina | 3.93675 | 41.868167 | worker | PAIRED |  |
| PRJNA729035 | SSR14703830 | *A.m.intermissa* | A Lineage | Morocco | Africa | Morocco | -4.52899 | 39.153618 | worker | PAIRED |  |
| PRJNA729035 | SSR14703831 | *A.m.intermissa* | A Lineage | Morocco | Africa | Morocco | -4.52899 | 39.153618 | worker | PAIRED |  |
| PRJNA729035 | SSR14703832 | *A.m.intermissa* | A Lineage | Morocco | Africa | Morocco | -4.52899 | 39.153618 | worker | PAIRED |  |
| PRJNA729035 | SSR14703833 | *A.m.intermissa* | A Lineage | Morocco | Africa | Morocco: Asilah | -4.52899 | 39.153618 | worker | PAIRED |  |
| PRJNA729035 | SSR14703834 | *A.m.intermissa* | A Lineage | Morocco | Africa | Morocco: Asilah | -3.306498 | 40.017988 | worker | PAIRED |  |
| PRJNA729035 | SSR14703835 | *A.m.intermissa* | A Lineage | Morocco | Africa | Morocco: Asilah | 0.4673 | 37.717283 | worker | PAIRED |  |
| PRJNA729035 | SSR14703836 | *A.m.intermissa* | A Lineage | Morocco | Africa | Morocco: Asilah | 0.4673 | 37.717283 | worker | PAIRED |  |
| PRJNA729035 | SSR14703837 | *A.m.intermissa* | A Lineage | Morocco | Africa | Morocco: Asilah | -0.500832 | 36.54557 | worker | PAIRED |  |
| PRJNA729035 | SSR14703838 | *A.m.intermissa* | A Lineage | Morocco | Africa | Morocco: Asilah | 3.88965 | 40.2674 | worker | PAIRED |  |
| PRJNA729035 | SSR14703839 | *A.m.intermissa* | A Lineage | Morocco | Africa | Morocco: Asilah | 3.88965 | 40.2674 | worker | PAIRED |  |
| PRJNA729035 | SSR14703840 | *A.m.adansonii* | A Lineage | Burkina Faso | Africa | Burkina Faso: Kurkina | -3.306498 | 40.017988 | worker | PAIRED |  |
| PRJNA729035 | SSR14703841 | *A.m.intermissa* | A Lineage | Morocco | Africa | Morocco: Asilah | -0.52504 | 36.403282 | worker | PAIRED |  |
| PRJNA729035 | SSR14703842 | *A.m.iberiensis* | M Lineage | Spain | Europe | Spain: Lugo | -0.52504 | 36.403282 | worker | PAIRED |  |
| PRJNA729035 | SSR14703843 | *A.m.iberiensis* | M Lineage | Spain | Europe | Spain: Cordoba | -0.52504 | 36.403282 | worker | PAIRED |  |
| PRJNA729035 | SSR14703844 | *A.m.iberiensis* | M Lineage | Spain | Europe | Spain: Cordoba | -0.500832 | 36.54557 | worker | PAIRED |  |
| PRJNA729035 | SSR14703845 | *A.m.iberiensis* | M Lineage | Spain | Europe | Spain: Cordoba | 0.9541 | 34.604767 | worker | PAIRED |  |
| PRJNA729035 | SSR14703846 | *A.m.caucasica* | O Lineage | Turkey | Asia | Turkey: Posov | 0.9541 | 34.604767 | worker | PAIRED |  |
| PRJNA729035 | SSR14703847 | *A.m.caucasica* | O Lineage | Turkey | Asia | Turkey: Posov | -0.500832 | 36.54557 | worker | PAIRED |  |
| PRJNA729035 | SSR14703848 | *A.m.caucasica* | O Lineage | Turkey | Asia | Turkey: Ardahan | 3.936617 | 41.868033 | worker | PAIRED |  |
| PRJNA729035 | SSR14703849 | *A.m.caucasica* | O Lineage | Turkey | Asia | Turkey: Ardahan | 3.936617 | 41.868033 | worker | PAIRED |  |
| PRJNA729035 | SSR14703850 | *A.m.caucasica* | O Lineage | Turkey | Asia | Turkey: Posov | 3.936617 | 41.868033 | worker | PAIRED |  |
| PRJNA729035 | SSR14703851 | *A.m.adansonii* | A Lineage | Burkina Faso | Africa | Burkina Faso: Kurkina | -3.306498 | 40.017988 | worker | PAIRED |  |
| PRJNA729035 | SSR14703852 | *A.m.capensis* | A Lineage | South Africa | Africa | South Africa | -3.306498 | 40.017988 | male | PAIRED | haploid |
| PRJNA729035 | SSR14703853 | *A.m.capensis* | A Lineage | South Africa | Africa | South Africa | -3.306498 | 40.017988 | male | PAIRED | haploid |
| PRJNA729035 | SSR14703854 | *A.m.capensis* | A Lineage | South Africa | Africa | South Africa | -18.9 | 47.5 | male | PAIRED | haploid |
| PRJNA729035 | SSR14703855 | *A.m.capensis* | A Lineage | South Africa | Africa | South Africa | -18.9 | 47.5 | male | PAIRED | haploid |
| PRJNA729035 | SSR14703856 | *A.m.mellifera* | A Lineage | Poland | Europe | Poland: Zalewo | -18.9 | 47.5 | worker | SINGLE | SINGLE |
| PRJNA729035 | SSR14703857 | *A.m.jemenitica* | Y Lineage | Yemen | Asia | Yemen: Sanaa | -18.9 | 47.5 | worker | PAIRED |  |
| PRJNA729035 | SSR14703858 | *A.m.jemenitica* | Y Lineage | Yemen | Asia | Yemen: Sanaa | -18.9 | 47.5 | worker | PAIRED |  |
| PRJNA729035 | SSR14703859 | *A.m.jemenitica* | Y Lineage | Yemen | Asia | Yemen: Sanaa | -18.9 | 47.5 | worker | PAIRED |  |
| PRJNA729035 | SSR14703860 | *A.m.jemenitica* | Y Lineage | Yemen | Asia | Yemen: Socotra Island | -18.9 | 47.5 | worker | PAIRED |  |
| PRJNA729035 | SSR14703861 | *A.m.jemenitica* | Y Lineage | Yemen | Asia | Yemen: Socotra Island | -18.9 | 47.5 | worker | PAIRED |  |
| PRJNA729035 | SSR14703862 | *A.m.jemenitica* | Y Lineage | Yemen | Asia | Yemen: Socotra Island | 27.18074 | 31.18339 | worker | PAIRED |  |
| PRJNA729035 | SSR14703863 | *A.m.jemenitica* | Y Lineage | Ethiopia | Africa | Ethiopia: Daba | 27.18074 | 31.18339 | worker | PAIRED |  |
| PRJNA729035 | SSR14703864 | *A.m.jemenitica* | Y Lineage | Ethiopia | Africa | Ethiopia: Daba | 27.18074 | 31.18339 | worker | PAIRED | Pairs with a kinship coefficient > 0.177 (twins and first-degree relationship) were closely related individuals |
| PRJNA729035 | SSR14703865 | *A.m.jemenitica* | Y Lineage | Ethiopia | Africa | Ethiopia: Daba | 27.18074 | 31.18339 | worker | PAIRED |  |
| PRJNA729035 | SSR14703866 | *A.m.capensis* | A Lineage | South Africa | Africa | South Africa: West Coast NP | 27.18074 | 31.18339 | worker | PAIRED |  |
| PRJNA729035 | SSR14703867 | *A.m.jemenitica* | Y Lineage | Ethiopia | Africa | Ethiopia: Daba | 27.18074 | 31.18339 | worker | PAIRED |  |
| PRJNA729035 | SSR14703868 | *A.m.jemenitica* | Y Lineage | Ethiopia | Africa | Ethiopia: Daba | 27.18074 | 31.18339 | worker | PAIRED | Pairs with a kinship coefficient > 0.177 (twins and first-degree relationship) were closely related individuals |
| PRJNA729035 | SSR14703869 | *A.m.jemenitica* | Y Lineage | Ethiopia | Africa | Ethiopia: Daba | 27.18074 | 31.18339 | worker | PAIRED |  |
| PRJNA729035 | SSR14703870 | *A.m.jemenitica* | Y Lineage | Ethiopia | Africa | Ethiopia: Daba | 30 | 31.2 | worker | PAIRED |  |
| PRJNA729035 | SSR14703871 | *A.m.unicolor* | U Lineage | Madagascar | Africa | Madagascar | 30 | 31.2 | worker | PAIRED |  |
| PRJNA729035 | SSR14703872 | *A.m.unicolor* | U Lineage | Madagascar | Africa | Madagascar | 30 | 31.2 | worker | PAIRED |  |
| PRJNA729035 | SSR14703873 | *A.m.unicolor* | U Lineage | Madagascar | Africa | Madagascar | 30 | 31.2 | worker | PAIRED |  |
| PRJNA729035 | SSR14703874 | *A.m.unicolor* | U Lineage | Madagascar | Africa | Madagascar | 30 | 31.2 | worker | PAIRED |  |
| PRJNA729035 | SSR14703875 | *A.m.unicolor* | U Lineage | Madagascar | Africa | Madagascar | 30 | 31.2 | worker | PAIRED |  |
| PRJNA729035 | SSR14703876 | *A.m.unicolor* | U Lineage | Madagascar | Africa | Madagascar | 48.483087 | 16.0909313 | worker | PAIRED |  |
| PRJNA729035 | SSR14703877 | *A.m.capensis* | A Lineage | South Africa | Africa | South Africa | 48.483087 | 16.0909313 | worker | PAIRED | Ambiguous Subspecies Assingment/Recent Admixture |
| PRJNA729035 | SSR14703878 | *A.m.unicolor* | U Lineage | Madagascar | Africa | Madagascar | 48.483087 | 16.0909313 | worker | PAIRED |  |
| PRJNA729035 | SSR14703879 | *A.m.unicolor* | U Lineage | Madagascar | Africa | Madagascar | 48.483087 | 16.0909313 | worker | PAIRED |  |
| PRJNA729035 | SSR14703880 | *A.m.syriaca* | O Lineage | Syria | Asia | Syria | 48.483087 | 16.0909313 | worker | PAIRED |  |
| PRJNA729035 | SSR14703881 | *A.m.syriaca* | O Lineage | Syria | Asia | Syria | 48.483087 | 16.0909313 | worker | PAIRED |  |
| PRJNA729035 | SSR14703882 | *A.m.syriaca* | O Lineage | Syria | Asia | Syria | 9.383214 | 41.965539 | worker | PAIRED |  |
| PRJNA729035 | SSR14703883 | *A.m.syriaca* | O Lineage | Syria | Asia | Syria | 9.383214 | 41.965539 | worker | PAIRED |  |
| PRJNA729035 | SSR14703884 | *A.m.syriaca* | O Lineage | Syria | Asia | Syria | 9.383214 | 41.965539 | worker | PAIRED |  |
| PRJNA729035 | SSR14703885 | *A.m.syriaca* | O Lineage | Syria | Asia | Syria | 9.383214 | 41.965539 | worker | PAIRED |  |
| PRJNA729035 | SSR14703886 | *A.m.syriaca* | O Lineage | Syria | Asia | Syria | 9.383214 | 41.965539 | worker | PAIRED |  |
| PRJNA729035 | SSR14703887 | *A.m.syriaca* | O Lineage | Syria | Asia | Syria | 40.29027 | 29.44833 | worker | PAIRED |  |
| PRJNA729035 | SSR14703888 | *A.m.capensis* | A Lineage | South Africa | Africa | South Africa | 40.29027 | 29.44833 | worker | PAIRED | Ambiguous Subspecies Assingment/Recent Admixture |
| PRJNA729035 | SSR14703889 | *A.m.syriaca* | O Lineage | Syria | Asia | Syria | 36.62166 | 29.11638 | worker | PAIRED |  |
| PRJNA729035 | SSR14703890 | *A.m.syriaca* | O Lineage | Turkey | Asia | Turkey: Hatay | 36.62166 | 29.11638 | worker | PAIRED |  |
| PRJNA729035 | SSR14703891 | *A.m.simensis* | ALineage | Kenya | Africa | Kenya: Mandera | 36.62166 | 29.11638 | worker | PAIRED |  |
| PRJNA729035 | SSR14703892 | *A.m.simensis* | A Lineage | Kenya | Africa | Kenya: Mandera | 38.60666 | 27.07 | worker | PAIRED |  |
| PRJNA729035 | SSR14703893 | *A.m.simensis* | A Lineage | Kenya | Africa | Kenya: Mandera | 38.60666 | 27.07 | worker | PAIRED | Pairs with a kinship coefficient > 0.177 (twins and first-degree relationship) were closely related individuals |
| PRJNA729035 | SSR14703894 | *A.m.scutellata* | A Lineage | South Africa | Africa | South Africa | 41.24194 | 42.36444 | male | PAIRED | haploid |
| PRJNA729035 | SSR14703895 | *A.m.scutellata* | A Lineage | South Africa | Africa | South Africa | 41.24194 | 42.36444 | male | PAIRED | haploid |
| PRJNA729035 | SSR14703896 | *A.m.capensis* | A Lineage | South Africa | Africa | South Africa: Heidelburg | 41.24194 | 42.36444 | worker | PAIRED |  |
| PRJNA729035 | SSR14703897 | *A.m.adansonii* | A Lineage | Burkina Faso | Africa | Burkina Faso: Kurkina | 41.24194 | 42.36444 | worker | PAIRED |  |
| PRJNA729035 | SSR14703898 | *A.m.adansonii* | A Lineage | Burkina Faso | Africa | Burkina Faso: Kurkina | 41.24194 | 42.36444 | worker | PAIRED |  |
| PRJNA729035 | SSR14703899 | *A.m.scutellata* | A Lineage | South Africa | Africa | South Africa | 33.5 | 36.3 | male | PAIRED | haploid |
| PRJNA729035 | SSR14703900 | *A.m.scutellata* | A Lineage | South Africa | Africa | South Africa | 33.5 | 36.3 | male | PAIRED | haploid |
| PRJNA729035 | SSR14703901 | *A.m.scutellata* | A Lineage | Zimbabwe | Africa | Zimbabwe: Harare | 33.5 | 36.3 | worker | PAIRED |  |
| PRJNA729035 | SSR14703902 | *A.m.capensis* | A Lineage | South Africa | Africa | South Africa: Cederberg | 33.5 | 36.3 | worker | PAIRED |  |
| PRJNA729035 | SSR14703903 | *A.m.scutellata* | A Lineage | Zimbabwe | Africa | Zimbabwe: Harare | 33.5 | 36.3 | worker | PAIRED | Ambiguous Subspecies Assingment/Recent Admixture |
| PRJNA729035 | SSR14703904 | *A.m.scutellata* | A Lineage | Zimbabwe | Africa | Zimbabwe: Harare | 33.5 | 36.3 | worker | PAIRED |  |
| PRJNA729035 | SSR14703905 | *A.m.scutellata* | A Lineage | Kenya | Africa | Kenya: Mandera | 33.5 | 36.3 | worker | PAIRED |  |
| PRJNA729035 | SSR14703906 | *A.m.scutellata* | A Lineage | Kenya | Africa | Kenya: Mandera | 33.5 | 36.3 | worker | PAIRED |  |
| PRJNA729035 | SSR14703907 | *A.m.scutellata* | A Lineage | Kenya | Africa | Kenya: Mandera | 33.5 | 36.3 | worker | PAIRED |  |
| PRJNA729035 | SSR14703908 | *A.m.scutellata* | A Lineage | Botswana | Africa | Botswana: Gaborone | 33.5 | 36.3 | worker | PAIRED |  |
| PRJNA729035 | SSR14703909 | *A.m.scutellata* | A Lineage | Kenya | Africa | Kenya: Tanzania border | 41.9140772 | 74.4137502 | worker | PAIRED |  |
| PRJNA729035 | SSR14703910 | *A.m.scutellata* | A Lineage | Kenya | Africa | Kenya: Tanzania border | 41.9140772 | 74.4137502 | worker | PAIRED |  |
| PRJNA729035 | SSR14703911 | *A.m.scutellata* | A Lineage | Kenya | Africa | Kenya: Tanzania border | 41.9140772 | 74.4137502 | worker | PAIRED |  |
| PRJNA729035 | SSR14703912 | *A.m.scutellata* | A Lineage | Kenya | Africa | Kenya: Gede Ruins | 41.9140772 | 74.4137502 | worker | PAIRED |  |
| PRJNA729035 | SSR14703913 | *A.m.capensis* | A Lineage | South Africa | Africa | South Africa: Somerset West | 41.9140772 | 74.4137502 | worker | PAIRED |  |
| PRJNA729035 | SSR14703914 | *A.m.scutellata* | A Lineage | Kenya | Africa | Kenya: Ichiara | 41.9140772 | 74.4137502 | worker | PAIRED |  |
| PRJNA729035 | SSR14703915 | *A.m.scutellata* | A Lineage | Kenya | Africa | Kenya: Ichiara | 44.5 | 11.35 | worker | PAIRED |  |
| PRJNA729035 | SSR14703916 | *A.m.scutellata* | A Lineage | Kenya | Africa | Kenya: Marchorwe | 44.5 | 11.35 | worker | PAIRED |  |
| PRJNA729035 | SSR14703917 | *A.m.scutellata* | A Lineage | Kenya | Africa | Kenya: Mandera | 45.46666 | 9.2 | worker | PAIRED | Pairs with a kinship coefficient > 0.177 (twins and first-degree relationship) were closely related individuals |
| PRJNA729035 | SSR14703918 | *A.m.scutellata* | A Lineage | Kenya | Africa | Kenya: Mandera | 45.46666 | 9.2 | worker | PAIRED | Admixed |
| PRJNA729035 | SSR14703919 | *A.m.scutellata* | A Lineage | Kenya | Africa | Kenya: Mandera | 45.46666 | 9.2 | worker | PAIRED |  |
| PRJNA729035 | SSR14703920 | *A.m.scutellata* | A Lineage | Kenya | Africa | Kenya: Gede Ruins | 45.46666 | 9.2 | worker | PAIRED |  |
| PRJNA729035 | SSR14703921 | *A.m.scutellata* | A Lineage | Kenya | Africa | Kenya: Malewa | 46 | 14.2 | worker | PAIRED |  |
| PRJNA729035 | SSR14703922 | *A.m.scutellata* | A Lineage | Kenya | Africa | Kenya: Malewa | 46 | 14.2 | worker | PAIRED |  |
| PRJNA729035 | SSR14703923 | *A.m.scutellata* | A Lineage | Kenya | Africa | Kenya: Malewa | 46 | 14.2 | worker | PAIRED |  |
| PRJNA729035 | SSR14703924 | *A.m.scutellata* | A Lineage | Kenya | Africa | Kenya: Marchorwe | 46 | 14.2 | worker | PAIRED |  |
| PRJNA729035 | SSR14703925 | *A.m.scutellata* | A Lineage | Kenya | Africa | Kenya: Moorland at Mt. Elgon | 46 | 14.2 | worker | PAIRED |  |
| PRJNA729035 | SSR14703926 | *A.m.scutellata* | A Lineage | Kenya | Africa | Kenya: Moorland at Mt. Elgon | 50.5 | 8.8 | worker | PAIRED |  |
| PRJNA729035 | SSR14703927 | *A.m.scutellata* | A Lineage | Kenya | Africa | Kenya: Marchorwe | 44 | 1 | worker | PAIRED |  |
| PRJNA729035 | SSR14703928 | *A.m.pomonella* | O Lineage | Kyrgyzstan | Asia | Kyrgyzstan: Dshuukn Valley | 43.93333 | 4.8 | worker | PAIRED |  |
| PRJNA729035 | SSR14703929 | *A.m.pomonella* | O Lineage | Kyrgyzstan | Asia | Kyrgyzstan: Dshuukn Valley | 43.93333 | 4.8 | worker | PAIRED |  |
| PRJNA729035 | SSR14703930 | *A.m.pomonella* | O Lineage | Kyrgyzstan | Asia | Kyrgyzstan: Dshuukn Valley | 42.67031 | 2.619786 | worker | PAIRED | Admixed |
| PRJNA729035 | SSR14703931 | *A.m.pomonella* | O Lineage | Kyrgyzstan | Asia | Kyrgyzstan: Dshuukn Valley | 42.67031 | 2.619786 | worker | PAIRED | Admixed |
| PRJNA729035 | SSR14703932 | *A.m.pomonella* | O Lineage | Kyrgyzstan | Asia | Kyrgyzstan: Tjurgon Ak-Suu Valley | 45.2 | -0.8 | worker | PAIRED |  |
| PRJNA729035 | SSR14703933 | *A.m.pomonella* | O Lineage | Kyrgyzstan | Asia | Kyrgyzstan: Tjurgon Ak-Suu Valley | 43 | -7.566667 | worker | PAIRED |  |
| PRJNA729035 | SSR14703934 | *A.m.capensis* | A Lineage | South Africa | Africa | South Africa: Robben Island\, | 37.88333 | -4.766667 | worker | PAIRED | Outgroup |
| PRJNA729035 | SSR14703935 | *A.m.pomonella* | O Lineage | Kyrgyzstan | Asia | Kyrgyzstan: Tjurgon Ak-Suu Valley | 37.88333 | -4.766667 | worker | PAIRED | Outgroup |
| PRJNA729035 | SSR14703936 | *A.m.pomonella* | O Lineage | Kyrgyzstan | Asia | Kyrgyzstan: Tjurgon Ak-Suu Valley | 37.88333 | -4.766667 | worker | PAIRED | Outgroup |
| PRJNA418874 | SSR6301296 | *Apis cerana* | Apis cerana | China | Asia | [China: Haikou, Hainan,](https://www.ncbi.nlm.nih.gov/biosample?term=) | 43.43 | 83.25 | worker | PAIRED | Outgroup |
| PRJNA418874 | SSR6301311 | *Apis cerana* | Apis cerana | China | Asia | [China: Haikou, Hainan,](https://www.ncbi.nlm.nih.gov/biosample?term=) | 43.43 | 83.25 | worker | PAIRED | Outgroup |
| PRJNA418874 | SSR6301393 | *Apis cerana* | Apis cerana | China | Asia | [China: Haikou, Hainan,](https://www.ncbi.nlm.nih.gov/biosample?term=) | 43.43 | 83.25 | worker | PAIRED | Outgroup |
| PRJNA418874 | SSR6301425 | *Apis cerana* | Apis cerana | China | Asia | [China: Haikou, Hainan,](https://www.ncbi.nlm.nih.gov/biosample?term=) | 43.43 | 83.25 | worker | PAIRED | Outgroup |
| PRJNA418874 | SSR6301438 | *Apis cerana* | Apis cerana | China | Asia | [China: Haikou, Hainan,](https://www.ncbi.nlm.nih.gov/biosample?term=) | 43.43 | 83.25 | worker | PAIRED | Outgroup |
| [PRJNA216922](https://www.ncbi.nlm.nih.gov/bioproject/PRJNA216922) | SSR957081 | *A. m. carnica* | C Lineage | Germany | Europe | Germany: North Rhine-Westphalia | 43.561 | 83.253 | worker | PAIRED |  |
| [PRJNA216922](https://www.ncbi.nlm.nih.gov/bioproject/PRJNA216922) | SSR957082 | *A. m. carnica* | C Lineage | Germany | Europe | Germany: North Rhine-Westphalia | 43.535 | 83.619 | worker | PAIRED |  |
| PRJNA216922 | SSR957084 | *A. m. carnica* | C Lineage | Germany | Europe | Germany: North Rhine-Westphalia | 43.714 | 83.543 | worker | PAIRED |  |
| [PRJNA216922](https://www.ncbi.nlm.nih.gov/bioproject/PRJNA216922) | SSR957085 | *A. m. carnica* | C Lineage | Germany | Europe | Germany: North Rhine-Westphalia | 43.827 | 83.432 | worker | PAIRED |  |
| PRJNA216922 | SSR957087 | *A. m. carnica* | C Lineage | Germany | Europe | Germany: North Rhine-Westphalia | 43.722 | 83.67 | worker | PAIRED |  |
| [PRJNA216922](https://www.ncbi.nlm.nih.gov/bioproject/PRJNA216922) | SSR957089 | *A. m. mellifera* | M Lineage | Germany | Europe | [Poland: Zalewo](https://www.ncbi.nlm.nih.gov/biosample?term=) | 43.473 | 82.197 | worker | PAIRED | Admixed |
| [PRJNA301648](https://www.ncbi.nlm.nih.gov/bioproject/PRJNA301648) | SRR2912421 | *A.m. sinisxinyuan* | M Lineage | China | Asia | China:Xinjiang -Yili | 43.43 | 83.25 | worker | PAIRED |  |
| [PRJNA301648](https://www.ncbi.nlm.nih.gov/bioproject/PRJNA301648) | SRR2912420 | *A.m. sinisxinyuan* | M Lineage | China | Asia | China:Xinjiang -Yili | 43.43 | 83.25 | worker | PAIRED |  |
| [PRJNA301648](https://www.ncbi.nlm.nih.gov/bioproject/PRJNA301648) | SRR2912414 | *A.m. sinisxinyuan* | M Lineage | China | Asia | China:Xinjiang -Yili | 43.43 | 83.25 | worker | PAIRED |  |
| [PRJNA301648](https://www.ncbi.nlm.nih.gov/bioproject/PRJNA301648) | SRR2912416 | *A.m. sinisxinyuan* | M Lineage | China | Asia | China:Xinjiang -Yili | 43.43 | 83.25 | worker | PAIRED |  |
| [PRJNA301648](https://www.ncbi.nlm.nih.gov/bioproject/PRJNA301648) | SRR2911291 | *A.m. sinisxinyuan* | M Lineage | China | Asia | China:Xinjiang -Yili | 43.43 | 83.25 | worker | PAIRED |  |
| XJ | yl491 | **Xinjiang black honeybee** | **C Lineage** | China | Asia | China:Xinjiang -Yili | 43.561 | 83.253 | worker | PAIRED |  |
| XJ | yl492 | **Xinjiang black honeybee** | **C Lineage** | China | Asia | China:Xinjiang -Yili | 43.535 | 83.619 | worker | PAIRED |  |
| XJ | yl493 | **Xinjiang black honeybee** | **C Lineage** | China | Asia | China:Xinjiang -Yili | 43.714 | 83.543 | worker | PAIRED |  |
| XJ | yl494 | **Xinjiang black honeybee** | **C Lineage** | China | Asia | China:Xinjiang -Yili | 43.827 | 83.432 | worker | PAIRED |  |
| XJ | yl495 | **Xinjiang black honeybee** | **C Lineage** | China | Asia | China:Xinjiang -Yili | 43.722 | 83.67 | worker | PAIRED |  |
| XJ | yl496 | **Xinjiang black honeybee** | **C Lineage** | China | Asia | China:Xinjiang -Yili | 43.473 | 82.197 | worker | PAIRED |  |
| XJ | yl497 | **Xinjiang black honeybee** | **C Lineage** | China | Asia | China:Xinjiang -Yili | 43.304173 | 82.607 | worker | PAIRED |  |
| XJ | yl498 | **Xinjiang black honeybee** | **C Lineage** | China | Asia | China:Xinjiang -Yili | 42.223 | 82.721 | worker | PAIRED |  |
| XJ | yl499 | **Xinjiang black honeybee** | **C Lineage** | China | Asia | China:Xinjiang -Yili | 43.148 | 82.731 | worker | PAIRED |  |
| XJ | yl500 | **Xinjiang black honeybee** | **C Lineage** | China | Asia | China:Xinjiang -Yili | 43.251 | 82.843 | worker | PAIRED |  |
| XJ | yl501 | **Xinjiang black honeybee** | **C Lineage** | China | Asia | China:Xinjiang -Yili | 42.668 | 80.456 | worker | PAIRED |  |
| XJ | yl502 | **Xinjiang black honeybee** | **C Lineage** | China | Asia | China:Xinjiang -Yili | 42.677 | 80.272 | worker | PAIRED |  |
| XJ | yl503 | **Xinjiang black honeybee** | **C Lineage** | China | Asia | China:Xinjiang -Yili | 42.656 | 80.396 | worker | PAIRED |  |
| XJ | yl504 | **Xinjiang black honeybee** | **C Lineage** | China | Asia | China:Xinjiang -Yili | 43.163 | 81.768 | worker | PAIRED |  |
| XJ | yl505 | **Xinjiang black honeybee** | **C Lineage** | China | Asia | China:Xinjiang -Yili | 43.123 | 81.909 | worker | PAIRED |  |
| XJ | yl506 | **Xinjiang black honeybee** | **C Lineage** | China | Asia | China:Xinjiang -Yili | 43.236 | 81.82 | worker | PAIRED |  |
| XJ | yl507 | **Xinjiang black honeybee** | **C Lineage** | China | Asia | China:Xinjiang -Yili | 43.242 | 81.804 | worker | PAIRED |  |
| XJ | yl509 | **Xinjiang black honeybee** | **C Lineage** | China | Asia | China:Xinjiang -Yili | 43.499 | 83.549 | worker | PAIRED |  |
| XJ | yl510 | **Xinjiang black honeybee** | **C Lineage** | China | Asia | China:Xinjiang -Yili | 43.571 | 83.149 | worker | PAIRED |  |

| **Table S3 The relationship of each sample in each population inferred by the King program. ID1: The first individual of the pair; ID2: The second individual of the pair; N_SNP: The number of SNPS that do not have missing SNPS in either of the individuals; HetHet: Percentage of SNPs with double heterozygotes; IBS0: Proportion of SNPs with 0-IBS (identical-by-state); Kinship: Kinship coefficient estimated by the program.** | | | | | | |
| --- | --- | --- | --- | --- | --- | --- |
| **Population** | **ID1** | **ID2** | **N_SNP** | **HetHet** | **IBS0** | **Kinship** |
| A | SRR1046114 | SRR14703837 | 20,653,112 | 0.034 | 0.0012 | 0.211 |
| A | SRR14703892 | SRR14703893 | 20611644 | 0.03 | 0.0015 | 0.2741 |
| A | SRR14703917 | SRR14703918 | 20650287 | 0.038 | 0.0002 | 0.3793 |
| Y | SRR14703864 | SRR14703867 | 20640777 | 0.034 | 0.0003 | 0.3513 |
| Y | SRR14703864 | SRR14703868 | 20564905 | 0.034 | 0.0004 | 0.3501 |
| Y | SRR14703867 | SRR14703868 | 20574006 | 0.037 | 0.0003 | 0.3816 |

| **Table S4. Excluded Apis mellifera samples and reasons for exclusion in downstream analysis.** | | | | |
| --- | --- | --- | --- | --- |
| **Run** | **Subspecies** | **bee** | **LibraryLayout** | **Reason for exclusion** |
| SSR1046114 | *A. m.intermissa* | worker | PAIRED | Pairs with a kinship coefficient > 0.177 |
| SSR14703785 | *A.m.capensis* | worker | PAIRED | Ambiguous Subspecies Assingment/Recent Admixture |
| SSR14703852 | *A.m.capensis* | male | PAIRED | haploid |
| SSR14703853 | *A.m.capensis* | male | PAIRED | haploid |
| SSR14703854 | *A.m.capensis* | male | PAIRED | haploid |
| SSR14703855 | *A.m.capensis* | male | PAIRED | haploid |
| SSR14703856 | *A.m.mellifera* | worker | SINGLE | SINGLE |
| SSR14703864 | *A.m.jemenitica* | worker | PAIRED | Pairs with a kinship coefficient > 0.177 |
| SSR14703868 | *A.m.jemenitica* | worker | PAIRED | Pairs with a kinship coefficient > 0.177 |
| SSR14703877 | *A.m.capensis* | worker | PAIRED | Ambiguous Subspecies Assingment/Recent Admixture |
| SSR14703888 | *A.m.capensis* | worker | PAIRED | Ambiguous Subspecies Assingment/Recent Admixture |
| SSR14703893 | *A.m.simensis* | worker | PAIRED | Pairs with a kinship coefficient > 0.177 |
| SSR14703894 | *A.m.scutellata* | male | PAIRED | haploid |
| SSR14703895 | *A.m.scutellata* | male | PAIRED | haploid |
| SSR14703899 | *A.m.scutellata* | male | PAIRED | haploid |
| SSR14703900 | *A.m.scutellata* | male | PAIRED | haploid |
| SSR14703903 | *A.m.scutellata* | worker | PAIRED | Ambiguous Subspecies Assingment/Recent Admixture |
| SSR14703917 | *A.m.scutellata* | worker | PAIRED | Pairs with a kinship coefficient > 0.177 |
| SSR14703930 | *A.m.pomonella* | worker | PAIRED | Admixed |
| SSR14703931 | *A.m.pomonella* | worker | PAIRED | Admixed |
| SSR6301296 | *Apis cerana* | worker | PAIRED | Outgroup |
| SSR6301311 | *Apis cerana* | worker | PAIRED | Outgroup |
| SSR6301393 | *Apis cerana* | worker | PAIRED | Outgroup |
| SSR6301425 | *Apis cerana* | worker | PAIRED | Outgroup |
| SSR6301438 | *Apis cerana* | worker | PAIRED | Outgroup |
| SSR957089 | *A. m. mellifera* | worker | PAIRED | Admixed |

| **Table S5 .Cross-validation (CV) error for varying values of K in the ADMIXTURE analysis** | |  |
| --- | --- | --- |
| **K** | **CV error** | |
| K=2 | 0.45321 | |
| K=3 | 0.41672 | |
| K=4 | 0.40391 | |
| K=5 | 0.38149 | |
| K=6 | 0.367 | |
| K=7 | 0.36957 | |
| K=8 | 0.37125 | |

| **Table S6. Pairwise FST values between Xinjiang black honeybee (XJ) and other *Apis mellifera* subspecies.** | | |
| --- | --- | --- |
| **population** | **subspecies** | **FST** |
| *XJ* | *A.m.adansonii* | 0.35477 |
| *XJ* | *A.m.anatoliaca* | 0.376 |
| *XJ* | *A.m.capensis* | 0.38106 |
| *XJ* | *A.m.carnica* | 0.05259 |
| *XJ* | *A.m.caucasica* | 0.41154 |
| *XJ* | *A.m.iberiensis* | 0.5344 |
| *XJ* | *A.m.intermissa* | 0.35453 |
| *XJ* | *A.m.jemenitica* | 0.33659 |
| *XJ* | *A.m.lamarckii* | 0.37025 |
| *XJ* | *A.m.lamarckii* | 0.43219 |
| *XJ* | *A.m.ligustica* | 0.05662 |
| *XJ* | *A.m.mellifera* | 0.54215 |
| *XJ* | *A.m.monticola* | 0.36401 |
| *XJ* | *A.m.pomonella* | 0.29818 |
| *XJ* | *A.m.scutellata* | 0.29372 |
| *XJ* | *A.m.sinisxinyuan* | 0.38584 |
| *XJ* | *A.m.syriaca* | 0.32887 |
| *XJ* | *A.m.unicolor* | 0.54663 |

| **Table S7.. D-statistics tests for detecting admixture among *Apis mellifera* populations using *A. cerana* as outgroup. Z (in parentheses) only showing |Z-scores| ≥ 3 groups.** | | | | | | | | | |
| --- | --- | --- | --- | --- | --- | --- | --- | --- | --- |
| *D (P1, P2; P3,Apis cerana) for all pair of Apis mellifera populations. D-statistics were implemented to detect admixture between X and Z (in parentheses) only showing |Z-scores| ≥ 3 groups.* | | | | | | | | |  |
| ***P1*** | ***P2*** | ***P3*** | **Dstatistic** | **Z-score** | **p-value** | **f4-ratio** | **ABBA** | **BABA** |  |
| *A.m.iberiensis* | *A.m.sinisxinyuan* | *A.m.carnica* | 0.322435 | 110.5 | 2.30E-16 | 0.295773 | 86160.1 | 44145.1 |  |
| *A.m.intermissa* | *A.m.mellifera* | *A.m.carnica* | 0.302187 | 102.251 | 2.30E-16 | 0.308807 | 120256 | 64442.5 |  |
| *A.m.iberiensis* | *A.m.sinisxinyuan* | *XJblackbee* | 0.36547 | 97.7631 | 2.30E-16 | 0.304052 | 88171.2 | 40972.8 |  |
| *A.m.iberiensis* | *A.m.sinisxinyuan* | *A.m.ligustica* | 0.423331 | 92.0928 | 2.30E-16 | 0.231015 | 92073.6 | 37304 |  |
| *A.m.anatoliaca* | *A.m.caucasica* | *A.m.pomonella* | 0.263608 | 90.7639 | 2.30E-16 | 1 | 75304.1 | 43884.9 |  |
| *A.m.syriaca* | *XJblackbee* | *A.m.sinisxinyuan* | 0.279859 | 85.1831 | 2.30E-16 | 0.243942 | 116787 | 65713 |  |
| *A.m.anatoliaca* | *A.m.carnica* | *A.m.mellifera* | 0.308972 | 77.6339 | 2.30E-16 | 0.211063 | 116325 | 61409.9 |  |
| *A.m.anatoliaca* | *XJblackbee* | *A.m.sinisxinyuan* | 0.288007 | 77.1792 | 2.30E-16 | 0.231474 | 106598 | 58925.9 |  |
| *A.m.caucasica* | *A.m.carnica* | *A.m.mellifera* | 0.306119 | 72.9819 | 2.30E-16 | 0.210207 | 116533 | 61908.8 |  |
| *A.m.caucasica* | *XJblackbee* | *A.m.sinisxinyuan* | 0.287309 | 71.4033 | 2.30E-16 | 0.230627 | 106278 | 58838.4 |  |
| *A.m.iberiensis* | *A.m.mellifera* | *A.m.carnica* | 0.197502 | 71.3416 | 2.30E-16 | 0.121186 | 52214.9 | 34991.5 |  |
| *A.m.intermissa* | *A.m.iberiensis* | *A.m.carnica* | 0.223498 | 69.0714 | 2.30E-16 | 0.213279 | 105473 | 66939.1 |  |
| *A.m.carnica* | *A.m.sinisxinyuan* | *A.m.intermissa* | 0.301036 | 69.0282 | 2.30E-16 | 0.51295 | 114322 | 61418.1 |  |
| *A.m.ligustica* | *A.m.sinisxinyuan* | *A.m.intermissa* | 0.320765 | 66.9154 | 2.30E-16 | 0.54184 | 122683 | 63092.8 |  |
| *A.m.sinisxinyuan* | *A.m.iberiensis* | *A.m.intermissa* | 0.244512 | 66.357 | 2.30E-16 | 0.607516 | 77769.9 | 47210.7 |  |
| *XJblackbee* | *A.m.sinisxinyuan* | *A.m.intermissa* | 0.292964 | 64.7836 | 2.30E-16 | 0.519262 | 120238 | 65750.3 |  |
| *A.m.carnica* | *XJblackbee* | *A.m.caucasica* | 0.223639 | 63.4793 | 2.30E-16 | 0.130959 | 78175 | 49599.7 |  |
| *A.m.anatoliaca* | *A.m.carnica* | *A.m.iberiensis* | 0.272145 | 63.0311 | 2.30E-16 | 0.149554 | 109555 | 62681.8 |  |
| *A.m.intermissa* | *A.m.mellifera* | *XJblackbee* | 0.216056 | 62.2622 | 2.30E-16 | 0.218575 | 110077 | 70962.2 |  |
| *A.m.intermissa* | *A.m.sinisxinyuan* | *A.m.pomonella* | 0.214694 | 61.1334 | 2.30E-16 | 0.198481 | 118711 | 76747.3 |  |
| *A.m.caucasica* | *A.m.carnica* | *A.m.iberiensis* | 0.272692 | 61.0883 | 2.30E-16 | 0.150154 | 109867 | 62786.1 |  |
| *A.m.caucasica* | *A.m.pomonella* | *A.m.mellifera* | 0.304679 | 59.9667 | 2.30E-16 | 0.106541 | 59578.1 | 31751.8 |  |
| *A.m.anatoliaca* | *A.m.ligustica* | *A.m.sinisxinyuan* | 0.270091 | 59.7453 | 2.30E-16 | 0.225309 | 109067 | 62679.5 |  |
| *A.m.carnica* | *XJblackbee* | *A.m.anatoliaca* | 0.205108 | 59.6616 | 2.30E-16 | 0.146216 | 76595.5 | 50522.6 |  |
| *A.m.anatoliaca* | *XJblackbee* | *A.m.mellifera* | 0.234118 | 59.0553 | 2.30E-16 | 0.141305 | 97388.8 | 60438.5 |  |
| *A.m.iberiensis* | *A.m.sinisxinyuan* | *A.m.anatoliaca* | 0.270583 | 59.0282 | 2.30E-16 | 0.115044 | 70792.4 | 40640.5 |  |
| *A.m.caucasica* | *A.m.pomonella* | *A.m.sinisxinyuan* | 0.26766 | 58.8169 | 2.30E-16 | 0.121444 | 59152 | 34172.7 |  |
| *A.m.mellifera* | *A.m.sinisxinyuan* | *XJblackbee* | 0.241657 | 58.4992 | 2.30E-16 | 0.226622 | 81356.7 | 49688.6 |  |
| *A.m.carnica* | *XJblackbee* | *A.m.syriaca* | 0.172876 | 58.3289 | 2.30E-16 | 0.158111 | 70469.3 | 49695.6 |  |
| *A.m.iberiensis* | *A.m.sinisxinyuan* | *A.m.caucasica* | 0.274666 | 58.1189 | 2.30E-16 | 0.101684 | 71011.5 | 40408.2 |  |
| *A.m.sinisxinyuan* | *A.m.carnica* | *A.m.anatoliaca* | 0.298333 | 57.94 | 2.30E-16 | 0.22958 | 115557 | 62451.5 |  |
| *A.m.carnica* | *XJblackbee* | *A.m.pomonella* | 0.167005 | 57.799 | 2.30E-16 | 0.165561 | 76813.2 | 54828.4 |  |
| *A.m.iberiensis* | *A.m.sinisxinyuan* | *A.m.pomonella* | 0.228903 | 57.603 | 2.30E-16 | 0.13965 | 73780.8 | 46295.1 |  |
| *A.m.mellifera* | *A.m.sinisxinyuan* | *A.m.ligustica* | 0.286331 | 56.8558 | 2.30E-16 | 0.171205 | 84611.8 | 46943.4 |  |
| *A.m.caucasica* | *A.m.ligustica* | *A.m.sinisxinyuan* | 0.266586 | 56.2739 | 2.30E-16 | 0.224428 | 109628 | 63479.9 |  |
| *A.m.caucasica* | *A.m.pomonella* | *A.m.iberiensis* | 0.313802 | 56.1349 | 2.30E-16 | 0.0892708 | 58838.4 | 30731.2 |  |
| *A.m.pomonella* | *A.m.caucasica* | *A.m.syriaca* | 0.251473 | 56.0998 | 2.30E-16 | 0.596975 | 71250.5 | 42616.2 |  |
| *A.m.mellifera* | *A.m.sinisxinyuan* | *A.m.carnica* | 0.187136 | 55.5829 | 2.30E-16 | 0.198491 | 78619.7 | 53833 |  |
| *A.m.caucasica* | *XJblackbee* | *A.m.mellifera* | 0.233142 | 55.3391 | 2.30E-16 | 0.140436 | 97006.7 | 60325.9 |  |
| *A.m.syriaca* | *A.m.carnica* | *A.m.iberiensis* | 0.216117 | 54.9645 | 2.30E-16 | 0.131432 | 113521 | 73173.5 |  |
| *A.m.anatoliaca* | *A.m.pomonella* | *A.m.mellifera* | 0.264324 | 54.8253 | 2.30E-16 | 0.107517 | 67238 | 39124 |  |
| *A.m.iberiensis* | *A.m.mellifera* | *XJblackbee* | 0.182313 | 54.642 | 2.30E-16 | 0.0999775 | 50352.6 | 34823.8 |  |
| *A.m.anatoliaca* | *A.m.pomonella* | *A.m.sinisxinyuan* | 0.231247 | 54.2453 | 2.30E-16 | 0.122492 | 67158.8 | 41931.9 |  |
| *A.m.sinisxinyuan* | *A.m.carnica* | *A.m.caucasica* | 0.290406 | 54.2079 | 2.30E-16 | 0.191247 | 114572 | 63003.2 |  |
| *A.m.intermissa* | *A.m.mellifera* | *A.m.ligustica* | 0.208264 | 54.0909 | 2.30E-16 | 0.143682 | 107165 | 70221.5 |  |
| *A.m.syriaca* | *A.m.pomonella* | *A.m.sinisxinyuan* | 0.201005 | 53.992 | 2.30E-16 | 0.136785 | 85550.4 | 56914.3 |  |
| *A.m.mellifera* | *A.m.carnica* | *A.m.syriaca* | 0.267808 | 53.0468 | 2.30E-16 | 0.281043 | 121468 | 70150.9 |  |
| *A.m.syriaca* | *XJblackbee* | *A.m.mellifera* | 0.191946 | 53.005 | 2.30E-16 | 0.130012 | 104208 | 70645.2 |  |
| *A.m.sinisxinyuan* | *A.m.intermissa* | *A.m.adansonii* | 0.189533 | 51.48 | 2.30E-16 | 0.497218 | 113977 | 77656.3 |  |
| *A.m.iberiensis* | *A.m.mellifera* | *A.m.ligustica* | 0.203739 | 50.4763 | 2.30E-16 | 0.0720888 | 50514.2 | 33414.6 |  |
| *A.m.carnica* | *A.m.syriaca* | *A.m.jemenitica* | 0.172304 | 50.4052 | 2.30E-16 | 0.30882 | 98214.7 | 69343.6 |  |
| *A.m.sinisxinyuan* | *A.m.intermissa* | *A.m.monticola* | 0.163676 | 50.3917 | 2.30E-16 | 0.344901 | 109058 | 78378.8 |  |
| *A.m.anatoliaca* | *A.m.pomonella* | *A.m.iberiensis* | 0.267457 | 50.08 | 2.30E-16 | 0.0886758 | 66124.6 | 38217.5 |  |
| *A.m.syriaca* | *A.m.pomonella* | *A.m.carnica* | 0.190199 | 49.5127 | 2.30E-16 | 0.220346 | 86402.3 | 58787.4 |  |
| *A.m.sinisxinyuan* | *A.m.intermissa* | *A.m.scutellata* | 0.161308 | 49.1558 | 2.30E-16 | 0.573194 | 108945 | 78679.5 |  |
| *A.m.syriaca* | *A.m.anatoliaca* | *XJblackbee* | 0.18437 | 48.9439 | 2.30E-16 | 0.244405 | 79347.2 | 54643.4 |  |
| *A.m.syriaca* | *A.m.caucasica* | *XJblackbee* | 0.194873 | 48.3945 | 2.30E-16 | 0.256637 | 79519.5 | 53581.7 |  |
| *XJblackbee* | *A.m.ligustica* | *A.m.carnica* | 0.183419 | 47.3552 | 2.30E-16 | 0.998976 | 69649 | 48059.2 |  |
| *A.m.sinisxinyuan* | *A.m.intermissa* | *A.m.lamarckiiA* | 0.160015 | 47.3545 | 2.30E-16 | 0.333872 | 108937 | 78883.2 |  |
| *A.m.pomonella* | *A.m.anatoliaca* | *A.m.syriaca* | 0.198534 | 47.0319 | 2.30E-16 | 0.563411 | 81638.9 | 54592.4 |  |
| *A.m.sinisxinyuan* | *A.m.intermissa* | *A.m.capensis* | 0.158495 | 46.7957 | 2.30E-16 | 0.328555 | 108477 | 78795.3 |  |
| *A.m.sinisxinyuan* | *A.m.intermissa* | *A.m.unicolor* | 0.158326 | 46.4792 | 2.30E-16 | 0.103832 | 108477 | 78822.6 |  |
| *A.m.anatoliaca* | *XJblackbee* | *A.m.iberiensis* | 0.199598 | 46.4301 | 2.30E-16 | 0.0972332 | 91960.8 | 61358.6 |  |
| *A.m.adansonii* | *A.m.intermissa* | *A.m.carnica* | 0.077755 | 46.1788 | 2.30E-16 | 0.0699951 | 94312.7 | 80704.2 |  |
| *A.m.syriaca* | *A.m.pomonella* | *XJblackbee* | 0.185478 | 45.572 | 2.30E-16 | 0.285826 | 92352.2 | 63453.6 |  |
| *A.m.sinisxinyuan* | *A.m.intermissa* | *A.m.jemenitica* | 0.139432 | 45.5364 | 2.30E-16 | 0.305094 | 105902 | 79983.8 |  |
| *A.m.sinisxinyuan* | *A.m.ligustica* | *A.m.syriaca* | 0.251456 | 45.5281 | 2.30E-16 | 0.272311 | 114416 | 68436.5 |  |
| *A.m.sinisxinyuan* | *A.m.mellifera* | *A.m.intermissa* | 0.159325 | 45.3401 | 2.30E-16 | 0.381722 | 70044.3 | 50792.1 |  |
| *A.m.caucasica* | *XJblackbee* | *A.m.iberiensis* | 0.202035 | 45.1284 | 2.30E-16 | 0.0979051 | 91707.6 | 60879.6 |  |
| *A.m.unicolor* | *A.m.lamarckii* | *A.m.ligustica* | 0.159057 | 45.0481 | 2.30E-16 | 0.110088 | 109218 | 79242 |  |
| *A.m.carnica* | *A.m.syriaca* | *A.m.monticola* | 0.162173 | 44.8314 | 2.30E-16 | 0.26314 | 95843.6 | 69095.1 |  |
| *A.m.iberiensis* | *A.m.sinisxinyuan* | *A.m.syriaca* | 0.183636 | 44.823 | 2.30E-16 | 0.106856 | 65071.4 | 44880.3 |  |
| *XJblackbee* | *A.m.syriaca* | *A.m.jemenitica* | 0.147372 | 44.8187 | 2.30E-16 | 0.265413 | 91316.8 | 67858.8 |  |
| *A.m.unicolor* | *A.m.lamarckii* | *A.m.carnica* | 0.147613 | 44.555 | 2.30E-16 | 0.133814 | 106051 | 78769.5 |  |
| *A.m.carnica* | *A.m.syriaca* | *A.m.scutellata* | 0.163544 | 44.3086 | 2.30E-16 | 0.419747 | 96149.1 | 69120.3 |  |
| *A.m.carnica* | *A.m.syriaca* | *A.m.lamarckiiA* | 0.165008 | 43.7897 | 2.30E-16 | 0.269746 | 96445.9 | 69125.3 |  |
| *A.m.mellifera* | *A.m.intermissa* | *A.m.adansonii* | 0.164158 | 43.7796 | 2.30E-16 | 0.449792 | 106499 | 76464.4 |  |
| *A.m.ligustica* | *XJblackbee* | *A.m.caucasica* | 0.189938 | 43.6854 | 2.30E-16 | 0.0951884 | 62684.7 | 42673.2 |  |
| *A.m.unicolor* | *A.m.lamarckii* | *XJblackbee* | 0.146792 | 43.6215 | 2.30E-16 | 0.142191 | 108775 | 80928.2 |  |
| *A.m.sinisxinyuan* | *A.m.carnica* | *A.m.syriaca* | 0.215558 | 43.6194 | 2.30E-16 | 0.22072 | 104781 | 67619.1 |  |
| *A.m.syriaca* | *A.m.anatoliaca* | *A.m.carnica* | 0.156787 | 43.3894 | 2.30E-16 | 0.154695 | 71494.9 | 52114.6 |  |
| *A.m.carnica* | *A.m.syriaca* | *A.m.capensis* | 0.163558 | 43.3469 | 2.30E-16 | 0.268592 | 96076.6 | 69066.2 |  |
| *A.m.ligustica* | *A.m.syriaca* | *A.m.jemenitica* | 0.163419 | 43.319 | 2.30E-16 | 0.292545 | 95496.9 | 68669.1 |  |
| *A.m.pomonella* | *A.m.carnica* | *A.m.sinisxinyuan* | 0.179386 | 43.0649 | 2.30E-16 | 0.187178 | 110717 | 77036.9 |  |
| *A.m.mellifera* | *A.m.intermissa* | *A.m.scutellata* | 0.143081 | 42.9042 | 2.30E-16 | 0.532213 | 102520 | 76855.1 |  |
| *A.m.mellifera* | *A.m.intermissa* | *A.m.monticola* | 0.144047 | 42.8555 | 2.30E-16 | 0.306794 | 102456 | 76655.6 |  |
| *A.m.carnica* | *A.m.syriaca* | *A.m.unicolor* | 0.163533 | 42.0283 | 2.30E-16 | 0.0910001 | 95989.1 | 69006.9 |  |
| *A.m.mellifera* | *A.m.iberiensis* | *A.m.intermissa* | 0.124125 | 42.0188 | 2.30E-16 | 0.36485 | 51381.7 | 40034.7 |  |
| *A.m.mellifera* | *A.m.intermissa* | *A.m.lamarckiiA* | 0.141589 | 41.5773 | 2.30E-16 | 0.297582 | 102495 | 77070.3 |  |
| *A.m.mellifera* | *A.m.intermissa* | *A.m.capensis* | 0.140438 | 41.0532 | 2.30E-16 | 0.292883 | 102093 | 76948.8 |  |
| *A.m.syriaca* | *A.m.pomonella* | *A.m.mellifera* | 0.177403 | 40.7618 | 2.30E-16 | 0.0958394 | 82094.1 | 57355.3 |  |
| *A.m.mellifera* | *A.m.intermissa* | *A.m.unicolor* | 0.140154 | 40.441 | 2.30E-16 | 0.0892399 | 102041 | 76954.4 |  |
| *A.m.monticola* | *A.m.lamarckii* | *A.m.carnica* | 0.13985 | 40.4106 | 2.30E-16 | 0.12648 | 104260 | 78676.4 |  |
| *A.m.mellifera* | *A.m.intermissa* | *A.m.jemenitica* | 0.125815 | 40.2326 | 2.30E-16 | 0.274278 | 99908.2 | 77577.7 |  |
| *A.m.monticola* | *A.m.lamarckii* | *A.m.ligustica* | 0.153063 | 40.2212 | 2.30E-16 | 0.105347 | 107529 | 78981.1 |  |
| *A.m.carnica* | *A.m.syriaca* | *A.m.adansonii* | 0.146289 | 39.9719 | 2.30E-16 | 0.261958 | 94549.7 | 70417 |  |
| *A.m.lamarckiiA* | *A.m.lamarckii* | *A.m.ligustica* | 0.115893 | 39.7739 | 2.30E-16 | 0.0789326 | 100011 | 79237.6 |  |
| *A.m.mellifera* | *A.m.sinisxinyuan* | *A.m.anatoliaca* | 0.187003 | 39.0246 | 2.30E-16 | 0.0827747 | 66462 | 45520.8 |  |
| *XJblackbee* | *A.m.syriaca* | *A.m.monticola* | 0.141122 | 39.0127 | 2.30E-16 | 0.226906 | 89365.2 | 67261.7 |  |
| *A.m.scutellata* | *A.m.lamarckii* | *A.m.ligustica* | 0.119793 | 38.9952 | 2.30E-16 | 0.0818565 | 101025 | 79410 |  |
| *XJblackbee* | *A.m.syriaca* | *A.m.lamarckiiA* | 0.142996 | 38.8268 | 2.30E-16 | 0.232227 | 89866.8 | 67380.9 |  |
| *XJblackbee* | *A.m.syriaca* | *A.m.scutellata* | 0.141138 | 38.7374 | 2.30E-16 | 0.370632 | 89535 | 67387.3 |  |
| *A.m.ligustica* | *A.m.syriaca* | *A.m.lamarckiiA* | 0.160036 | 38.7237 | 2.30E-16 | 0.258884 | 94061.6 | 68108.5 |  |
| *A.m.adansonii* | *A.m.lamarckii* | *A.m.carnica* | 0.100066 | 38.7074 | 2.30E-16 | 0.0912043 | 97466.4 | 79734.6 |  |
| *A.m.monticola* | *A.m.lamarckii* | *XJblackbee* | 0.140621 | 38.5346 | 2.30E-16 | 0.135731 | 107061 | 80663.2 |  |
| *A.m.ligustica* | *A.m.carnica* | *A.m.mellifera* | 0.208297 | 38.5017 | 2.30E-16 | 0.111092 | 74438.4 | 48773.7 |  |
| *XJblackbee* | *A.m.syriaca* | *A.m.adansonii* | 0.131612 | 38.4604 | 2.30E-16 | 0.231433 | 88509.2 | 67921.1 |  |
| *A.m.syriaca* | *A.m.pomonella* | *A.m.ligustica* | 0.181121 | 38.3795 | 2.30E-16 | 0.14544 | 88330.8 | 61240.3 |  |
| *A.m.ligustica* | *A.m.syriaca* | *A.m.adansonii* | 0.151207 | 38.3011 | 2.30E-16 | 0.26309 | 92824.6 | 68440.3 |  |
| *A.m.scutellata* | *A.m.lamarckii* | *A.m.carnica* | 0.107023 | 38.2342 | 2.30E-16 | 0.0967353 | 97874.9 | 78950.5 |  |
| *XJblackbee* | *A.m.syriaca* | *A.m.capensis* | 0.140012 | 38.2287 | 2.30E-16 | 0.228984 | 89348.2 | 67401.4 |  |
| *A.m.ligustica* | *A.m.syriaca* | *A.m.scutellata* | 0.157933 | 38.1544 | 2.30E-16 | 0.40472 | 93705.4 | 68144.1 |  |
| *A.m.ligustica* | *A.m.syriaca* | *A.m.monticola* | 0.157971 | 37.9137 | 2.30E-16 | 0.253243 | 93542.4 | 68020.2 |  |
| *A.m.syriaca* | *A.m.caucasica* | *A.m.carnica* | 0.148266 | 37.8255 | 2.30E-16 | 0.144473 | 70080 | 51982.4 |  |
| *A.m.iberiensis* | *A.m.intermissa* | *A.m.adansonii* | 0.143177 | 37.6887 | 2.30E-16 | 0.409203 | 101556 | 76117.1 |  |
| *A.m.ligustica* | *XJblackbee* | *A.m.anatoliaca* | 0.160032 | 37.5969 | 2.30E-16 | 0.0988487 | 60707.5 | 43957.8 |  |
| *A.m.iberiensis* | *A.m.mellifera* | *A.m.pomonella* | 0.114442 | 37.5192 | 2.30E-16 | 0.0477533 | 45777.9 | 36376.1 |  |
| *A.m.lamarckiiA* | *A.m.lamarckii* | *A.m.carnica* | 0.102481 | 37.5136 | 2.30E-16 | 0.0924459 | 96813.1 | 78814.5 |  |
| *A.m.mellifera* | *A.m.sinisxinyuan* | *A.m.pomonella* | 0.149604 | 37.4239 | 2.30E-16 | 0.0964181 | 69454.1 | 51377.3 |  |
| *A.m.lamarckiiA* | *A.m.lamarckii* | *XJblackbee* | 0.102983 | 37.356 | 2.30E-16 | 0.099545 | 99510.1 | 80928.1 |  |
| *A.m.intermissa* | *A.m.mellifera* | *A.m.pomonella* | 0.132907 | 37.2369 | 2.30E-16 | 0.112901 | 101759 | 77883 |  |
| *A.m.ligustica* | *A.m.syriaca* | *A.m.capensis* | 0.15642 | 37.1863 | 2.30E-16 | 0.255058 | 93487.6 | 68196.9 |  |
| *A.m.syriaca* | *A.m.anatoliaca* | *A.m.ligustica* | 0.156505 | 37.1789 | 2.30E-16 | 0.107585 | 74026 | 53990.8 |  |
| *A.m.anatoliaca* | *A.m.ligustica* | *A.m.mellifera* | 0.18301 | 37.1101 | 2.30E-16 | 0.113436 | 95850.4 | 66194.5 |  |
| *XJblackbee* | *A.m.syriaca* | *A.m.unicolor* | 0.140987 | 36.9814 | 2.30E-16 | 0.075551 | 89485.7 | 67370.9 |  |
| *A.m.scutellata* | *A.m.lamarckii* | *XJblackbee* | 0.107232 | 36.9245 | 2.30E-16 | 0.103849 | 100566 | 81087 |  |
| *A.m.mellifera* | *A.m.sinisxinyuan* | *A.m.caucasica* | 0.186148 | 36.7462 | 2.30E-16 | 0.0717153 | 66577.8 | 45681.1 |  |
| *A.m.adansonii* | *A.m.lamarckii* | *A.m.ligustica* | 0.122529 | 36.4611 | 2.30E-16 | 0.0835688 | 101264 | 79156.9 |  |
| *A.m.intermissa* | *A.m.iberiensis* | *XJblackbee* | 0.13902 | 36.2609 | 2.30E-16 | 0.131637 | 96474.7 | 72924.7 |  |
| *A.m.ligustica* | *XJblackbee* | *A.m.pomonella* | 0.128263 | 36.223 | 2.30E-16 | 0.111074 | 61085.8 | 47197.1 |  |
| *A.m.ligustica* | *A.m.syriaca* | *A.m.unicolor* | 0.157639 | 36.2079 | 2.30E-16 | 0.0861923 | 93687.1 | 68171.9 |  |
| *A.m.iberiensis* | *A.m.intermissa* | *A.m.scutellata* | 0.127524 | 35.9667 | 2.30E-16 | 0.496264 | 98239.2 | 76017.2 |  |
| *A.m.syriaca* | *XJblackbee* | *A.m.iberiensis* | 0.140765 | 35.7363 | 2.30E-16 | 0.0778931 | 97283.8 | 73275.1 |  |
| *A.m.iberiensis* | *A.m.intermissa* | *A.m.monticola* | 0.127836 | 35.4817 | 2.30E-16 | 0.276282 | 98116.1 | 75873.8 |  |
| *XJblackbee* | *A.m.carnica* | *A.m.mellifera* | 0.1317 | 35.449 | 2.30E-16 | 0.0819933 | 78812.5 | 60469.1 |  |
| *A.m.iberiensis* | *A.m.intermissa* | *A.m.lamarckiiA* | 0.126321 | 35.3311 | 2.30E-16 | 0.26873 | 98237 | 76201.8 |  |
| *A.m.caucasica* | *A.m.ligustica* | *A.m.mellifera* | 0.180064 | 35.3223 | 2.30E-16 | 0.112522 | 96279.7 | 66897.3 |  |
| *A.m.capensis* | *A.m.lamarckii* | *A.m.carnica* | 0.0828031 | 35.1156 | 2.30E-16 | 0.0751026 | 93824.1 | 79474.4 |  |
| *A.m.adansonii* | *A.m.lamarckii* | *XJblackbee* | 0.107851 | 35.0912 | 2.30E-16 | 0.104452 | 100686 | 81082 |  |
| *A.m.iberiensis* | *A.m.intermissa* | *A.m.capensis* | 0.125757 | 34.9102 | 2.30E-16 | 0.264942 | 97911.6 | 76036.5 |  |
| *A.m.sinisxinyuan* | *A.m.intermissa* | *A.m.lamarckii* | 0.119234 | 34.8319 | 2.30E-16 | 0.13212 | 105683 | 83165.4 |  |
| *A.m.capensis* | *A.m.lamarckii* | *A.m.ligustica* | 0.094802 | 34.6582 | 2.30E-16 | 0.0646919 | 96821.4 | 80053.4 |  |
| *A.m.iberiensis* | *A.m.intermissa* | *A.m.unicolor* | 0.124876 | 34.4276 | 2.30E-16 | 0.0782368 | 97822.7 | 76103.4 |  |
| *A.m.ligustica* | *A.m.carnica* | *A.m.iberiensis* | 0.212199 | 34.1935 | 2.30E-16 | 0.0873166 | 72877.8 | 47362.8 |  |
| *A.m.mellifera* | *A.m.intermissa* | *A.m.lamarckii* | 0.118423 | 34.0581 | 2.30E-16 | 0.125565 | 100353 | 79101.5 |  |
| *A.m.pomonella* | *A.m.syriaca* | *A.m.jemenitica* | 0.108401 | 33.8542 | 2.30E-16 | 0.178964 | 72326.4 | 58179.5 |  |
| *A.m.iberiensis* | *A.m.intermissa* | *A.m.jemenitica* | 0.113126 | 33.7731 | 2.30E-16 | 0.24822 | 95903.4 | 76410.3 |  |
| *A.m.syriaca* | *A.m.ligustica* | *A.m.mellifera* | 0.148415 | 33.353 | 2.30E-16 | 0.101765 | 101610 | 75346.7 |  |
| *A.m.unicolor* | *A.m.intermissa* | *A.m.carnica* | 0.119966 | 33.0628 | 2.30E-16 | 0.113721 | 108227 | 85041.4 |  |
| *A.m.capensis* | *A.m.lamarckii* | *XJblackbee* | 0.0825224 | 33.0542 | 2.30E-16 | 0.080413 | 96408.4 | 81709.7 |  |
| *A.m.unicolor* | *A.m.lamarckii* | *A.m.syriaca* | 0.112271 | 33.0267 | 2.30E-16 | 0.123419 | 112173 | 89527.5 |  |
| *A.m.unicolor* | *A.m.lamarckii* | *A.m.pomonella* | 0.108251 | 32.9779 | 2.30E-16 | 0.0927836 | 107181 | 86242.6 |  |
| *A.m.anatoliaca* | *A.m.syriaca* | *A.m.jemenitica* | 0.0937199 | 32.9477 | 2.30E-16 | 0.143199 | 63284 | 52438.5 |  |
| *A.m.carnica* | *A.m.iberiensis* | *A.m.adansonii* | 0.162676 | 32.5937 | 2.30E-16 | 0.326882 | 107340 | 77302.8 |  |
| *A.m.mellifera* | *A.m.sinisxinyuan* | *A.m.syriaca* | 0.129283 | 32.5544 | 2.30E-16 | 0.0774962 | 61960.8 | 47773.9 |  |
| *A.m.syriaca* | *A.m.pomonella* | *A.m.iberiensis* | 0.155255 | 32.2768 | 2.30E-16 | 0.0692055 | 79353.4 | 58024.8 |  |
| *A.m.carnica* | *A.m.syriaca* | *A.m.lamarckii* | 0.128759 | 32.0821 | 2.30E-16 | 0.129343 | 97802.1 | 75489.3 |  |
| *A.m.carnica* | *A.m.anatoliaca* | *A.m.jemenitica* | 0.1207 | 31.9416 | 2.30E-16 | 0.19324 | 83790.7 | 65742.2 |  |
| *A.m.carnica* | *A.m.pomonella* | *A.m.jemenitica* | 0.101986 | 31.7161 | 2.30E-16 | 0.158181 | 79889 | 65101.9 |  |
| *A.m.caucasica* | *A.m.syriaca* | *A.m.jemenitica* | 0.0978357 | 31.69 | 2.30E-16 | 0.147444 | 62928.3 | 51712.3 |  |
| *A.m.pomonella* | *A.m.carnica* | *A.m.mellifera* | 0.146811 | 31.6666 | 2.30E-16 | 0.11582 | 105065 | 78164.6 |  |
| *A.m.syriaca* | *A.m.caucasica* | *A.m.ligustica* | 0.142692 | 31.651 | 2.30E-16 | 0.096733 | 72119.4 | 54107.8 |  |
| *A.m.jemenitica* | *A.m.lamarckii* | *A.m.ligustica* | 0.0830222 | 31.5907 | 2.30E-16 | 0.057745 | 96904.1 | 82047.2 |  |
| *A.m.iberiensis* | *A.m.mellifera* | *A.m.caucasica* | 0.127628 | 31.5161 | 2.30E-16 | 0.0322139 | 42847.2 | 33148.1 |  |
| *A.m.iberiensis* | *A.m.mellifera* | *A.m.anatoliaca* | 0.121267 | 31.4948 | 2.30E-16 | 0.0351199 | 42571.9 | 33363.5 |  |
| *A.m.monticola* | *A.m.intermissa* | *A.m.carnica* | 0.113049 | 31.4751 | 2.30E-16 | 0.106161 | 105716 | 84241.7 |  |
| *A.m.ligustica* | *XJblackbee* | *A.m.syriaca* | 0.121883 | 31.2599 | 2.30E-16 | 0.0982298 | 55630.1 | 43542.7 |  |
| *A.m.unicolor* | *A.m.lamarckii* | *A.m.anatoliaca* | 0.105637 | 30.7097 | 2.30E-16 | 0.0788304 | 107501 | 86958.6 |  |
| *A.m.jemenitica* | *A.m.lamarckii* | *A.m.carnica* | 0.0727864 | 30.6013 | 2.30E-16 | 0.0672883 | 93960.5 | 81210.4 |  |
| *A.m.jemenitica* | *A.m.syriaca* | *A.m.sinisxinyuan* | 0.0922302 | 30.5592 | 2.30E-16 | 0.0800801 | 107917 | 89691.8 |  |
| *A.m.iberiensis* | *A.m.intermissa* | *A.m.lamarckii* | 0.111724 | 30.4884 | 2.30E-16 | 0.116071 | 96639.1 | 77215.3 |  |
| *A.m.intermissa* | *A.m.sinisxinyuan* | *A.m.anatoliaca* | 0.123916 | 30.4351 | 2.30E-16 | 0.0899573 | 104020 | 81082.6 |  |
| *A.m.carnica* | *A.m.mellifera* | *A.m.adansonii* | 0.149032 | 30.367 | 2.30E-16 | 0.27819 | 98764.5 | 73144.5 |  |
| *A.m.intermissa* | *A.m.sinisxinyuan* | *A.m.caucasica* | 0.127595 | 30.3296 | 2.30E-16 | 0.080247 | 104282 | 80681.4 |  |
| *A.m.unicolor* | *A.m.lamarckii* | *A.m.caucasica* | 0.105683 | 30.2135 | 2.30E-16 | 0.0684414 | 107292 | 86781.6 |  |
| *A.m.adansonii* | *A.m.iberiensis* | *A.m.ligustica* | 0.136986 | 29.6595 | 2.30E-16 | 0.102829 | 112830 | 85642.3 |  |
| *XJblackbee* | *A.m.carnica* | *A.m.iberiensis* | 0.122674 | 29.4145 | 2.30E-16 | 0.058687 | 76091.5 | 59462.6 |  |
| *A.m.carnica* | *A.m.sinisxinyuan* | *A.m.adansonii* | 0.126811 | 29.3977 | 2.30E-16 | 0.209274 | 85489.5 | 66247.5 |  |
| *A.m.jemenitica* | *A.m.lamarckii* | *XJblackbee* | 0.0707907 | 29.3482 | 2.30E-16 | 0.0705509 | 96492.6 | 83734.2 |  |
| *A.m.carnica* | *A.m.pomonella* | *A.m.scutellata* | 0.0971346 | 29.3281 | 2.30E-16 | 0.216483 | 78732.5 | 64791.4 |  |
| *A.m.carnica* | *A.m.caucasica* | *A.m.jemenitica* | 0.117736 | 29.2622 | 2.30E-16 | 0.18906 | 83761.4 | 66115.5 |  |
| *A.m.anatoliaca* | *A.m.syriaca* | *A.m.lamarckii* | 0.106784 | 29.2131 | 2.30E-16 | 0.0773027 | 65427.1 | 52802.1 |  |
| *A.m.carnica* | *A.m.pomonella* | *A.m.lamarckiiA* | 0.0975317 | 29.1985 | 2.30E-16 | 0.138377 | 78855.8 | 64840.8 |  |
| *A.m.pomonella* | *A.m.syriaca* | *A.m.monticola* | 0.101111 | 29.1043 | 2.30E-16 | 0.146801 | 70562.6 | 57603.6 |  |
| *A.m.monticola* | *A.m.lamarckii* | *A.m.pomonella* | 0.101974 | 29.1033 | 2.30E-16 | 0.0869461 | 105392 | 85887 |  |
| *A.m.caucasica* | *A.m.syriaca* | *A.m.lamarckii* | 0.111873 | 29.0084 | 2.30E-16 | 0.0799778 | 65088.6 | 51990.6 |  |
| *A.m.unicolor* | *A.m.lamarckii* | *A.m.sinisxinyuan* | 0.0941965 | 28.9814 | 2.30E-16 | 0.0757097 | 105093 | 86998.6 |  |
| *A.m.carnica* | *A.m.anatoliaca* | *A.m.scutellata* | 0.112031 | 28.8668 | 2.30E-16 | 0.257519 | 82207.1 | 65643.3 |  |
| *A.m.carnica* | *A.m.pomonella* | *A.m.monticola* | 0.0966525 | 28.8521 | 2.30E-16 | 0.136174 | 78527.1 | 64685.3 |  |
| *A.m.pomonella* | *A.m.syriaca* | *A.m.lamarckii* | 0.110063 | 28.7572 | 2.30E-16 | 0.0887746 | 74069 | 59381 |  |
| *A.m.carnica* | *A.m.pomonella* | *A.m.capensis* | 0.0969806 | 28.7151 | 2.30E-16 | 0.138414 | 78725.5 | 64805.7 |  |
| *A.m.pomonella* | *A.m.syriaca* | *A.m.scutellata* | 0.102355 | 28.5954 | 2.30E-16 | 0.25929 | 70821.2 | 57669.6 |  |
| *A.m.carnica* | *A.m.pomonella* | *A.m.unicolor* | 0.0981347 | 28.5638 | 2.30E-16 | 0.0474324 | 78683.6 | 64620.5 |  |
| *A.m.carnica* | *A.m.anatoliaca* | *A.m.monticola* | 0.109421 | 28.5386 | 2.30E-16 | 0.158961 | 81833.7 | 65691.3 |  |
| *A.m.monticola* | *A.m.lamarckii* | *A.m.syriaca* | 0.10629 | 28.5385 | 2.30E-16 | 0.116503 | 110443 | 89220.6 |  |
| *A.m.carnica* | *A.m.anatoliaca* | *A.m.lamarckiiA* | 0.11227 | 28.434 | 2.30E-16 | 0.164234 | 82324.4 | 65705.1 |  |
| *A.m.sinisxinyuan* | *A.m.iberiensis* | *A.m.adansonii* | 0.103425 | 28.2769 | 2.30E-16 | 0.147693 | 57410.8 | 46648.5 |  |
| *A.m.carnica* | *A.m.anatoliaca* | *A.m.capensis* | 0.114487 | 28.2041 | 2.30E-16 | 0.168518 | 82412.7 | 65480.8 |  |
| *A.m.pomonella* | *A.m.syriaca* | *A.m.lamarckiiA* | 0.103825 | 28.2014 | 2.30E-16 | 0.152499 | 71063.3 | 57694.9 |  |
| *A.m.carnica* | *A.m.pomonella* | *A.m.adansonii* | 0.0936939 | 28.0196 | 2.30E-16 | 0.146367 | 78694.1 | 65211 |  |
| *A.m.anatoliaca* | *A.m.lamarckii* | *A.m.iberiensis* | 0.092001 | 28.0127 | 2.30E-16 | 0.0575514 | 107487 | 89375.7 |  |
| *A.m.pomonella* | *A.m.syriaca* | *A.m.capensis* | 0.102411 | 27.9162 | 2.30E-16 | 0.151087 | 70773.1 | 57623.9 |  |
| *A.m.anatoliaca* | *A.m.syriaca* | *A.m.adansonii* | 0.0874211 | 27.8377 | 2.30E-16 | 0.126339 | 61538.2 | 51643.7 |  |
| *A.m.pomonella* | *XJblackbee* | *A.m.sinisxinyuan* | 0.129547 | 27.7795 | 2.30E-16 | 0.124076 | 97776.2 | 75348.5 |  |
| *A.m.intermissa* | *A.m.lamarckii* | *A.m.syriaca* | 0.0927526 | 27.7342 | 2.30E-16 | 0.102422 | 108198 | 89830.8 |  |
| *A.m.carnica* | *A.m.anatoliaca* | *A.m.unicolor* | 0.111661 | 27.4829 | 2.30E-16 | 0.0556606 | 82113.4 | 65617.6 |  |
| *A.m.adansonii* | *A.m.intermissa* | *XJblackbee* | 0.0490412 | 27.4579 | 2.30E-16 | 0.0463747 | 93089.9 | 84386.3 |  |
| *A.m.caucasica* | *A.m.lamarckii* | *A.m.iberiensis* | 0.0929916 | 27.3349 | 2.30E-16 | 0.0581717 | 107636 | 89320.6 |  |
| *A.m.pomonella* | *A.m.syriaca* | *A.m.unicolor* | 0.100965 | 27.2462 | 2.30E-16 | 0.0457114 | 70659.6 | 57699.8 |  |
| *A.m.anatoliaca* | *A.m.syriaca* | *A.m.lamarckiiA* | 0.0941863 | 27.1855 | 2.30E-16 | 0.126345 | 62393.2 | 51651.8 |  |
| *A.m.intermissa* | *A.m.iberiensis* | *A.m.ligustica* | 0.119768 | 27.1748 | 2.30E-16 | 0.0770491 | 92576.3 | 72772.7 |  |
| *A.m.caucasica* | *A.m.syriaca* | *A.m.adansonii* | 0.0919692 | 27.0774 | 2.30E-16 | 0.130965 | 61204.3 | 50894.6 |  |
| *A.m.anatoliaca* | *A.m.syriaca* | *A.m.monticola* | 0.093638 | 27.0452 | 2.30E-16 | 0.123711 | 62068.9 | 51440.1 |  |
| *A.m.caucasica* | *A.m.syriaca* | *A.m.lamarckiiA* | 0.0987414 | 26.8912 | 2.30E-16 | 0.130582 | 62057.6 | 50903.6 |  |
| *A.m.anatoliaca* | *A.m.syriaca* | *A.m.scutellata* | 0.0922637 | 26.8272 | 2.30E-16 | 0.218292 | 62131.8 | 51635.2 |  |
| *XJblackbee* | *A.m.iberiensis* | *A.m.adansonii* | 0.13472 | 26.8029 | 2.30E-16 | 0.300184 | 112168 | 85533.8 |  |
| *A.m.ligustica* | *A.m.mellifera* | *A.m.adansonii* | 0.137705 | 26.7108 | 2.30E-16 | 0.280613 | 107405 | 81405.3 |  |
| *A.m.unicolor* | *A.m.jemenitica* | *A.m.anatoliaca* | 0.0835963 | 26.6509 | 2.30E-16 | 0.0617461 | 104286 | 88194.9 |  |
| *A.m.caucasica* | *A.m.syriaca* | *A.m.monticola* | 0.0978983 | 26.6482 | 2.30E-16 | 0.127564 | 61725.2 | 50717.3 |  |
| *A.m.monticola* | *A.m.lamarckii* | *A.m.anatoliaca* | 0.100104 | 26.6357 | 2.30E-16 | 0.0742158 | 105774 | 86524.2 |  |
| *A.m.scutellata* | *A.m.intermissa* | *A.m.carnica* | 0.0800006 | 26.6155 | 2.30E-16 | 0.0757014 | 99963.7 | 85154.1 |  |
| *XJblackbee* | *A.m.carnica* | *A.m.sinisxinyuan* | 0.0823027 | 26.6098 | 2.30E-16 | 0.0732028 | 75956.4 | 64404.3 |  |
| *A.m.caucasica* | *A.m.syriaca* | *A.m.scutellata* | 0.0968143 | 26.5202 | 2.30E-16 | 0.225088 | 61817.6 | 50904.5 |  |
| *A.m.anatoliaca* | *A.m.syriaca* | *A.m.intermissa* | 0.0817078 | 26.5049 | 2.30E-16 | 0.0879146 | 61034.4 | 51813.8 |  |
| *A.m.monticola* | *A.m.lamarckii* | *A.m.caucasica* | 0.0997223 | 26.3234 | 2.30E-16 | 0.0641021 | 105500 | 86366.7 |  |
| *A.m.ligustica* | *A.m.anatoliaca* | *A.m.jemenitica* | 0.109558 | 26.3094 | 2.30E-16 | 0.17395 | 80721.2 | 64780.4 |  |
| *A.m.unicolor* | *A.m.jemenitica* | *A.m.caucasica* | 0.084132 | 26.2553 | 2.30E-16 | 0.0539357 | 104143 | 87979.7 |  |
| *A.m.carnica* | *A.m.caucasica* | *A.m.scutellata* | 0.108738 | 26.2035 | 2.30E-16 | 0.250663 | 82137.5 | 66026.5 |  |
| *A.m.anatoliaca* | *A.m.syriaca* | *A.m.unicolor* | 0.0923285 | 26.0682 | 2.30E-16 | 0.0373958 | 62160.5 | 51652.3 |  |
| *A.m.unicolor* | *A.m.jemenitica* | *XJblackbee* | 0.0820826 | 26.0039 | 2.30E-16 | 0.0771414 | 99581.8 | 84474 |  |
| *A.m.unicolor* | *A.m.jemenitica* | *A.m.pomonella* | 0.0799032 | 25.9967 | 2.30E-16 | 0.0676083 | 103104 | 87846.5 |  |
| *A.m.carnica* | *A.m.caucasica* | *A.m.monticola* | 0.106395 | 25.9629 | 2.30E-16 | 0.154973 | 81783.9 | 66054.6 |  |
| *XJblackbee* | *A.m.syriaca* | *A.m.lamarckii* | 0.10275 | 25.9236 | 2.30E-16 | 0.100962 | 90871.5 | 73937.4 |  |
| *A.m.unicolor* | *A.m.jemenitica* | *A.m.carnica* | 0.0812029 | 25.883 | 2.30E-16 | 0.0713871 | 96897.6 | 82342.8 |  |
| *A.m.carnica* | *A.m.caucasica* | *A.m.lamarckiiA* | 0.109012 | 25.8005 | 2.30E-16 | 0.159927 | 82270.4 | 66096.6 |  |
| *A.m.carnica* | *A.m.caucasica* | *A.m.capensis* | 0.111334 | 25.7117 | 2.30E-16 | 0.164276 | 82330 | 65834.4 |  |
| *A.m.caucasica* | *A.m.syriaca* | *A.m.intermissa* | 0.0860944 | 25.666 | 2.30E-16 | 0.0914294 | 60690.8 | 51068.9 |  |
| *A.m.anatoliaca* | *A.m.syriaca* | *A.m.capensis* | 0.0889399 | 25.6117 | 2.30E-16 | 0.120397 | 61874.4 | 51767.2 |  |
| *A.m.caucasica* | *A.m.syriaca* | *A.m.unicolor* | 0.0970469 | 25.5278 | 2.30E-16 | 0.0388787 | 61826.6 | 50888 |  |
| *A.m.anatoliaca* | *A.m.ligustica* | *A.m.iberiensis* | 0.138258 | 25.4954 | 2.30E-16 | 0.0691035 | 89508.2 | 67764.1 |  |
| *A.m.unicolor* | *A.m.jemenitica* | *A.m.ligustica* | 0.0832073 | 25.4537 | 2.30E-16 | 0.055592 | 98529.6 | 83392.4 |  |
| *A.m.lamarckiiA* | *A.m.intermissa* | *A.m.carnica* | 0.0752086 | 25.3353 | 2.30E-16 | 0.0713284 | 99271.1 | 85383.5 |  |
| *A.m.carnica* | *XJblackbee* | *A.m.jemenitica* | 0.0553046 | 25.28 | 2.30E-16 | 0.0575363 | 51346.2 | 45964.4 |  |
| *A.m.caucasica* | *A.m.syriaca* | *A.m.capensis* | 0.0933269 | 25.274 | 2.30E-16 | 0.124589 | 61537.5 | 51031.8 |  |
| *A.m.scutellata* | *A.m.lamarckii* | *A.m.pomonella* | 0.0687356 | 25.2502 | 2.30E-16 | 0.0585219 | 98985.4 | 86252.9 |  |
| *XJblackbee* | *A.m.anatoliaca* | *A.m.jemenitica* | 0.0907752 | 25.1685 | 2.30E-16 | 0.142239 | 75466.4 | 62905.6 |  |
| *A.m.caucasica* | *A.m.ligustica* | *A.m.iberiensis* | 0.138921 | 25.0654 | 2.30E-16 | 0.0697692 | 90032.8 | 68069.1 |  |
| *A.m.scutellata* | *A.m.lamarckii* | *A.m.syriaca* | 0.0737007 | 25.0596 | 2.30E-16 | 0.081431 | 103948 | 89678 |  |
| *A.m.carnica* | *A.m.caucasica* | *A.m.unicolor* | 0.108249 | 24.9175 | 2.30E-16 | 0.0540783 | 82008.2 | 65987.8 |  |
| *A.m.unicolor* | *A.m.jemenitica* | *A.m.syriaca* | 0.0823574 | 24.8415 | 2.30E-16 | 0.0894596 | 107865 | 91450.2 |  |
| *A.m.ligustica* | *A.m.sinisxinyuan* | *A.m.adansonii* | 0.121483 | 24.7838 | 2.30E-16 | 0.211475 | 90306.9 | 70742.2 |  |
| *A.m.unicolor* | *A.m.adansonii* | *A.m.mellifera* | 0.094639 | 24.5106 | 2.30E-16 | 0.0700696 | 108994 | 90147.6 |  |
| *A.m.intermissa* | *A.m.adansonii* | *A.m.monticola* | 0.0383431 | 24.418 | 2.30E-16 | 0.131913 | 104193 | 96497.7 |  |
| *A.m.pomonella* | *A.m.syriaca* | *A.m.adansonii* | 0.0836574 | 24.3801 | 2.30E-16 | 0.135263 | 69349.9 | 58642.4 |  |
| *A.m.lamarckiiA* | *A.m.lamarckii* | *A.m.syriaca* | 0.067723 | 24.3751 | 2.30E-16 | 0.0748786 | 102699 | 89671.5 |  |
| *A.m.unicolor* | *A.m.adansonii* | *A.m.sinisxinyuan* | 0.0878542 | 24.3715 | 2.30E-16 | 0.0715089 | 105807 | 88717.2 |  |
| *A.m.ligustica* | *A.m.caucasica* | *A.m.jemenitica* | 0.106267 | 24.3416 | 2.30E-16 | 0.169735 | 80913.9 | 65368.8 |  |
| *A.m.lamarckiiA* | *A.m.lamarckii* | *A.m.pomonella* | 0.0636591 | 24.2705 | 2.30E-16 | 0.0540864 | 97844.6 | 86132.8 |  |
| *A.m.adansonii* | *A.m.lamarckii* | *A.m.syriaca* | 0.0823117 | 24.1851 | 2.30E-16 | 0.0901688 | 104852 | 88903.8 |  |
| *XJblackbee* | *A.m.mellifera* | *A.m.adansonii* | 0.119319 | 24.1473 | 2.30E-16 | 0.24951 | 104071 | 81883.1 |  |
| *A.m.ligustica* | *A.m.anatoliaca* | *A.m.scutellata* | 0.10448 | 24.146 | 2.30E-16 | 0.238058 | 79398.4 | 64376.7 |  |
| *A.m.unicolor* | *A.m.adansonii* | *A.m.iberiensis* | 0.0992298 | 24.1294 | 2.30E-16 | 0.0646971 | 110998 | 90957.8 |  |
| *A.m.ligustica* | *A.m.anatoliaca* | *A.m.lamarckiiA* | 0.105405 | 24.0703 | 2.30E-16 | 0.151483 | 79567.9 | 64393.7 |  |
| *A.m.ligustica* | *A.m.anatoliaca* | *A.m.capensis* | 0.105321 | 24.0069 | 2.30E-16 | 0.152801 | 79447 | 64306.6 |  |
| *A.m.ligustica* | *A.m.syriaca* | *A.m.lamarckii* | 0.106764 | 23.98 | 2.30E-16 | 0.107747 | 94344.1 | 76142.2 |  |
| *A.m.carnica* | *A.m.anatoliaca* | *A.m.adansonii* | 0.096383 | 23.8785 | 2.30E-16 | 0.155104 | 81191.3 | 66916.3 |  |
| *A.m.adansonii* | *A.m.lamarckii* | *A.m.pomonella* | 0.0645762 | 23.8091 | 2.30E-16 | 0.0552553 | 98734.7 | 86756.4 |  |
| *A.m.intermissa* | *A.m.lamarckii* | *A.m.anatoliaca* | 0.0784694 | 23.8091 | 2.30E-16 | 0.0586301 | 102776 | 87820.4 |  |
| *A.m.carnica* | *XJblackbee* | *A.m.lamarckii* | 0.0526758 | 23.6181 | 2.30E-16 | 0.0308182 | 53134.9 | 47817.2 |  |
| *A.m.ligustica* | *A.m.anatoliaca* | *A.m.monticola* | 0.103458 | 23.5874 | 2.30E-16 | 0.147426 | 79166.6 | 64321.6 |  |
| *A.m.carnica* | *XJblackbee* | *A.m.scutellata* | 0.0501014 | 23.3946 | 2.30E-16 | 0.0751223 | 50733.4 | 45892.3 |  |
| *A.m.intermissa* | *A.m.lamarckii* | *A.m.caucasica* | 0.0781685 | 23.3849 | 2.30E-16 | 0.050532 | 102531 | 87663.8 |  |
| *A.m.iberiensis* | *A.m.mellifera* | *A.m.syriaca* | 0.0788187 | 23.3769 | 2.30E-16 | 0.0316982 | 41009.6 | 35017.3 |  |
| *A.m.caucasica* | *A.m.pomonella* | *A.m.carnica* | 0.101363 | 23.3718 | 2.30E-16 | 0.0886504 | 51631.9 | 42128.1 |  |
| *A.m.sinisxinyuan* | *A.m.iberiensis* | *A.m.monticola* | 0.0811787 | 23.3029 | 2.30E-16 | 0.0939411 | 55539 | 47198.9 |  |
| *A.m.ligustica* | *A.m.anatoliaca* | *A.m.unicolor* | 0.103976 | 23.2447 | 2.30E-16 | 0.0505818 | 79459 | 64491.6 |  |
| *A.m.ligustica* | *A.m.anatoliaca* | *A.m.adansonii* | 0.100505 | 23.131 | 2.30E-16 | 0.156008 | 79105.3 | 64656.6 |  |
| *A.m.carnica* | *XJblackbee* | *A.m.monticola* | 0.0478153 | 23.1269 | 2.30E-16 | 0.0453307 | 50512.6 | 45902.5 |  |
| *A.m.intermissa* | *A.m.adansonii* | *A.m.scutellata* | 0.0373637 | 22.9524 | 2.30E-16 | 0.333621 | 104290 | 96777.7 |  |
| *XJblackbee* | *A.m.caucasica* | *A.m.jemenitica* | 0.088426 | 22.8616 | 2.30E-16 | 0.13782 | 74853.5 | 62691 |  |
| *XJblackbee* | *A.m.anatoliaca* | *A.m.scutellata* | 0.0848551 | 22.8017 | 2.30E-16 | 0.194358 | 74166.7 | 62564.4 |  |
| *A.m.carnica* | *A.m.iberiensis* | *A.m.monticola* | 0.116557 | 22.7976 | 2.30E-16 | 0.209342 | 101698 | 80465.4 |  |
| *A.m.ligustica* | *A.m.pomonella* | *A.m.adansonii* | 0.0972057 | 22.719 | 2.30E-16 | 0.147441 | 77121.1 | 63456.2 |  |
| *XJblackbee* | *A.m.anatoliaca* | *A.m.lamarckiiA* | 0.0854409 | 22.7073 | 2.30E-16 | 0.120924 | 74311.3 | 62612.4 |  |
| *XJblackbee* | *A.m.anatoliaca* | *A.m.capensis* | 0.0862731 | 22.6606 | 2.30E-16 | 0.123146 | 74248.2 | 62454.5 |  |
| *XJblackbee* | *A.m.anatoliaca* | *A.m.monticola* | 0.0836921 | 22.5833 | 2.30E-16 | 0.117341 | 73933.1 | 62513.6 |  |
| *A.m.unicolor* | *A.m.capensis* | *A.m.carnica* | 0.0731555 | 22.4909 | 2.30E-16 | 0.0634851 | 94937.6 | 81994.1 |  |
| *A.m.intermissa* | *A.m.iberiensis* | *A.m.pomonella* | 0.0850339 | 22.4124 | 2.30E-16 | 0.0684736 | 92351.4 | 77876.3 |  |
| *A.m.ligustica* | *A.m.caucasica* | *A.m.scutellata* | 0.100863 | 22.3951 | 2.30E-16 | 0.231166 | 79555.9 | 64977.8 |  |
| *A.m.anatoliaca* | *A.m.jemenitica* | *A.m.iberiensis* | 0.0751196 | 22.3596 | 2.30E-16 | 0.0474353 | 106834 | 91904.7 |  |
| *A.m.monticola* | *A.m.jemenitica* | *A.m.pomonella* | 0.0727661 | 22.3434 | 2.30E-16 | 0.0615859 | 101849 | 88032 |  |
| *A.m.ligustica* | *A.m.caucasica* | *A.m.lamarckiiA* | 0.101814 | 22.2941 | 2.30E-16 | 0.147194 | 79739.7 | 65002.8 |  |
| *A.m.scutellata* | *A.m.lamarckii* | *A.m.anatoliaca* | 0.0651761 | 22.233 | 2.30E-16 | 0.048124 | 99210.1 | 87069.1 |  |
| *A.m.ligustica* | *A.m.caucasica* | *A.m.capensis* | 0.101879 | 22.23 | 2.30E-16 | 0.148616 | 79595.1 | 64876.5 |  |
| *A.m.carnica* | *XJblackbee* | *A.m.lamarckiiA* | 0.0496751 | 22.1927 | 2.30E-16 | 0.0473963 | 50744.5 | 45941.6 |  |
| *A.m.syriaca* | *A.m.lamarckii* | *A.m.iberiensis* | 0.059976 | 22.1769 | 2.30E-16 | 0.0373526 | 101727 | 90215.2 |  |
| *A.m.unicolor* | *A.m.capensis* | *XJblackbee* | 0.0724331 | 22.17 | 2.30E-16 | 0.0671575 | 97364.8 | 84212.6 |  |
| *A.m.monticola* | *A.m.jemenitica* | *A.m.anatoliaca* | 0.0773312 | 22.1233 | 2.30E-16 | 0.0570482 | 103075 | 88277.5 |  |
| *A.m.carnica* | *XJblackbee* | *A.m.capensis* | 0.0520592 | 22.1123 | 2.30E-16 | 0.0499777 | 50811.3 | 45782.7 |  |
| *A.m.caucasica* | *A.m.pomonella* | *A.m.intermissa* | 0.0819426 | 22.1037 | 2.30E-16 | 0.0629871 | 43758.7 | 37130.4 |  |
| *A.m.ligustica* | *A.m.carnica* | *A.m.sinisxinyuan* | 0.103971 | 22.0231 | 2.30E-16 | 0.0808504 | 68298.2 | 55433.7 |  |
| *A.m.unicolor* | *A.m.capensis* | *A.m.ligustica* | 0.0735858 | 21.9942 | 2.30E-16 | 0.0485162 | 96366 | 83155.7 |  |
| *A.m.adansonii* | *A.m.intermissa* | *A.m.ligustica* | 0.0422366 | 21.9865 | 2.30E-16 | 0.0278847 | 91010.4 | 83634 |  |
| *A.m.ligustica* | *A.m.caucasica* | *A.m.monticola* | 0.100151 | 21.962 | 2.30E-16 | 0.143519 | 79341.6 | 64896.1 |  |
| *A.m.scutellata* | *A.m.lamarckii* | *A.m.caucasica* | 0.0649783 | 21.9505 | 2.30E-16 | 0.0414477 | 98991.3 | 86911.6 |  |
| *A.m.unicolor* | *A.m.lamarckii* | *A.m.mellifera* | 0.0757379 | 21.9454 | 2.30E-16 | 0.0549051 | 104884 | 90115.3 |  |
| *A.m.carnica* | *XJblackbee* | *A.m.unicolor* | 0.0500202 | 21.9384 | 2.30E-16 | 0.0162518 | 50587.5 | 45767.8 |  |
| *A.m.pomonella* | *A.m.carnica* | *A.m.iberiensis* | 0.107166 | 21.9367 | 2.30E-16 | 0.0667017 | 98426.7 | 79372.6 |  |
| *A.m.monticola* | *A.m.jemenitica* | *A.m.caucasica* | 0.0774565 | 21.9179 | 2.30E-16 | 0.0495493 | 102870 | 88079.7 |  |
| *A.m.caucasica* | *A.m.jemenitica* | *A.m.iberiensis* | 0.0761295 | 21.8337 | 2.30E-16 | 0.0480446 | 106920 | 91792.2 |  |
| *A.m.monticola* | *A.m.lamarckii* | *A.m.sinisxinyuan* | 0.0799182 | 21.7753 | 2.30E-16 | 0.0644365 | 102875 | 87648.7 |  |
| *XJblackbee* | *A.m.anatoliaca* | *A.m.unicolor* | 0.0845064 | 21.7516 | 2.30E-16 | 0.0395116 | 74178.8 | 62618.5 |  |
| *A.m.monticola* | *A.m.jemenitica* | *A.m.carnica* | 0.0719946 | 21.7271 | 2.30E-16 | 0.063457 | 95566.2 | 82729.8 |  |
| *A.m.pomonella* | *A.m.ligustica* | *A.m.sinisxinyuan* | 0.118768 | 21.7262 | 2.30E-16 | 0.117053 | 99616.3 | 78465.8 |  |
| *A.m.lamarckiiA* | *A.m.lamarckii* | *A.m.anatoliaca* | 0.0602132 | 21.7162 | 2.30E-16 | 0.0443397 | 98079.1 | 86938.6 |  |
| *XJblackbee* | *A.m.sinisxinyuan* | *A.m.adansonii* | 0.0974182 | 21.6823 | 2.30E-16 | 0.177585 | 88807 | 73040.2 |  |
| *A.m.monticola* | *A.m.jemenitica* | *A.m.syriaca* | 0.0756036 | 21.6052 | 2.30E-16 | 0.0823306 | 106692 | 91693.2 |  |
| *A.m.unicolor* | *A.m.intermissa* | *XJblackbee* | 0.0868398 | 21.5865 | 2.30E-16 | 0.086754 | 106319 | 89329.1 |  |
| *XJblackbee* | *A.m.pomonella* | *A.m.adansonii* | 0.0729622 | 21.5195 | 2.30E-16 | 0.110901 | 72540.3 | 62674.7 |  |
| *A.m.carnica* | *A.m.caucasica* | *A.m.adansonii* | 0.0931201 | 21.4759 | 2.30E-16 | 0.15029 | 81124.4 | 67302.9 |  |
| *A.m.ligustica* | *A.m.caucasica* | *A.m.unicolor* | 0.100274 | 21.4418 | 2.30E-16 | 0.0490503 | 79601.2 | 65092.3 |  |
| *A.m.sinisxinyuan* | *A.m.iberiensis* | *A.m.scutellata* | 0.077216 | 21.4131 | 2.30E-16 | 0.15078 | 55410.4 | 47466.7 |  |
| *A.m.ligustica* | *A.m.syriaca* | *A.m.intermissa* | 0.0883041 | 21.3843 | 2.30E-16 | 0.132174 | 89740.2 | 75177.3 |  |
| *A.m.monticola* | *A.m.jemenitica* | *XJblackbee* | 0.0745664 | 21.3491 | 2.30E-16 | 0.0701665 | 98333.1 | 84686.1 |  |
| *A.m.anatoliaca* | *A.m.syriaca* | *A.m.iberiensis* | 0.0602114 | 21.27 | 2.30E-16 | 0.0209705 | 58106.3 | 51506.4 |  |
| *A.m.unicolor* | *A.m.capensis* | *A.m.anatoliaca* | 0.0704575 | 21.2666 | 2.30E-16 | 0.0512436 | 101441 | 88087.2 |  |
| *A.m.ligustica* | *A.m.caucasica* | *A.m.adansonii* | 0.0968651 | 21.2545 | 2.30E-16 | 0.151277 | 79275.8 | 65274 |  |
| *A.m.unicolor* | *A.m.capensis* | *A.m.pomonella* | 0.0679349 | 21.2229 | 2.30E-16 | 0.0566388 | 100467 | 87684.6 |  |
| *A.m.lamarckiiA* | *A.m.lamarckii* | *A.m.caucasica* | 0.0599846 | 21.2091 | 2.30E-16 | 0.0381323 | 97845.7 | 86771.5 |  |
| *A.m.ligustica* | *A.m.pomonella* | *A.m.jemenitica* | 0.0893487 | 21.1648 | 2.30E-16 | 0.138297 | 77305.9 | 64624.6 |  |
| *A.m.intermissa* | *A.m.lamarckii* | *A.m.ligustica* | 0.0779652 | 21.1372 | 2.30E-16 | 0.0571809 | 101650 | 86946.3 |  |
| *A.m.unicolor* | *A.m.capensis* | *A.m.caucasica* | 0.0708229 | 21.112 | 2.30E-16 | 0.0447055 | 101281 | 87884.2 |  |
| *A.m.adansonii* | *A.m.lamarckii* | *A.m.anatoliaca* | 0.0709747 | 21.0122 | 2.30E-16 | 0.0521744 | 99725.8 | 86508 |  |
| *A.m.ligustica* | *A.m.pomonella* | *A.m.monticola* | 0.089607 | 20.9805 | 2.30E-16 | 0.124586 | 76333 | 63778.1 |  |
| *A.m.intermissa* | *A.m.adansonii* | *A.m.lamarckiiA* | 0.0361772 | 20.9778 | 2.30E-16 | 0.121459 | 104233 | 96954.3 |  |
| *A.m.monticola* | *A.m.jemenitica* | *A.m.ligustica* | 0.0757098 | 20.8414 | 2.30E-16 | 0.0505368 | 97293.6 | 83598.3 |  |
| *XJblackbee* | *A.m.anatoliaca* | *A.m.adansonii* | 0.0778446 | 20.8311 | 2.30E-16 | 0.119823 | 73734 | 63083.5 |  |
| *A.m.carnica* | *A.m.mellifera* | *A.m.monticola* | 0.105044 | 20.8285 | 2.30E-16 | 0.175463 | 93797.2 | 75964.7 |  |
| *A.m.sinisxinyuan* | *A.m.iberiensis* | *A.m.lamarckiiA* | 0.0769486 | 20.7459 | 2.30E-16 | 0.0881848 | 55452.2 | 47528 |  |
| *A.m.adansonii* | *A.m.lamarckii* | *A.m.caucasica* | 0.0708131 | 20.7394 | 2.30E-16 | 0.0449873 | 99487 | 86328.8 |  |
| *XJblackbee* | *A.m.caucasica* | *A.m.scutellata* | 0.0820743 | 20.6014 | 2.30E-16 | 0.186985 | 73533.5 | 62378.6 |  |
| *A.m.capensis* | *A.m.lamarckii* | *A.m.pomonella* | 0.0448875 | 20.5862 | 2.30E-16 | 0.0383046 | 94951.9 | 86793.8 |  |
| *A.m.intermissa* | *A.m.adansonii* | *A.m.jemenitica* | 0.0320505 | 20.5745 | 2.30E-16 | 0.10782 | 102439 | 96076.5 |  |
| *A.m.ligustica* | *A.m.pomonella* | *A.m.lamarckiiA* | 0.0894677 | 20.5536 | 2.30E-16 | 0.125491 | 76595.3 | 64015.2 |  |
| *A.m.monticola* | *A.m.adansonii* | *A.m.mellifera* | 0.0804408 | 20.5138 | 2.30E-16 | 0.0590133 | 105385 | 89693 |  |
| *XJblackbee* | *A.m.caucasica* | *A.m.lamarckiiA* | 0.0827191 | 20.5004 | 2.30E-16 | 0.11644 | 73684.7 | 62425.7 |  |
| *XJblackbee* | *A.m.caucasica* | *A.m.capensis* | 0.0836594 | 20.4719 | 2.30E-16 | 0.118731 | 73608 | 62242.8 |  |
| *XJblackbee* | *A.m.caucasica* | *A.m.monticola* | 0.0812133 | 20.4719 | 2.30E-16 | 0.113227 | 73317.4 | 62303.2 |  |
| *A.m.ligustica* | *A.m.pomonella* | *A.m.scutellata* | 0.0883602 | 20.4675 | 2.30E-16 | 0.196455 | 76421.1 | 64012.4 |  |
| *A.m.monticola* | *A.m.adansonii* | *A.m.iberiensis* | 0.0844576 | 20.4052 | 2.30E-16 | 0.054502 | 107269 | 90560.5 |  |
| *A.m.intermissa* | *A.m.adansonii* | *A.m.capensis* | 0.0353987 | 20.3227 | 2.30E-16 | 0.116985 | 103838 | 96737.6 |  |
| *A.m.monticola* | *A.m.intermissa* | *XJblackbee* | 0.0806484 | 20.3074 | 2.30E-16 | 0.0797772 | 103954 | 88438 |  |
| *A.m.intermissa* | *A.m.adansonii* | *A.m.unicolor* | 0.0362322 | 20.2616 | 2.30E-16 | 0.0283885 | 103934 | 96666.2 |  |
| *A.m.lamarckii* | *A.m.syriaca* | *A.m.sinisxinyuan* | 0.0598712 | 20.2367 | 2.30E-16 | 0.0529563 | 103616 | 91909.8 |  |
| *A.m.monticola* | *A.m.adansonii* | *A.m.sinisxinyuan* | 0.0747209 | 20.1951 | 2.30E-16 | 0.0602465 | 102379 | 88143 |  |
| *A.m.sinisxinyuan* | *A.m.iberiensis* | *A.m.capensis* | 0.0750606 | 20.1578 | 2.30E-16 | 0.0854962 | 55217.9 | 47507.3 |  |
| *A.m.ligustica* | *A.m.pomonella* | *A.m.unicolor* | 0.0893693 | 20.1283 | 2.30E-16 | 0.042387 | 76471.1 | 63924.1 |  |
| *A.m.mellifera* | *A.m.iberiensis* | *A.m.adansonii* | 0.0599665 | 20.1092 | 2.30E-16 | 0.0674192 | 39693.7 | 35202.4 |  |
| *A.m.carnica* | *A.m.iberiensis* | *A.m.scutellata* | 0.107302 | 20.0688 | 2.30E-16 | 0.304842 | 101016 | 81438 |  |
| *A.m.sinisxinyuan* | *A.m.iberiensis* | *A.m.unicolor* | 0.0762986 | 20.047 | 2.30E-16 | 0.027503 | 55348.5 | 47501.2 |  |
| *A.m.capensis* | *A.m.lamarckii* | *A.m.syriaca* | 0.0509931 | 20.0248 | 2.30E-16 | 0.0568138 | 99915.9 | 90220.3 |  |
| *A.m.sinisxinyuan* | *A.m.mellifera* | *A.m.adansonii* | 0.0620899 | 19.9371 | 2.30E-16 | 0.0864107 | 53957.5 | 47648.8 |  |
| *A.m.unicolor* | *A.m.capensis* | *A.m.sinisxinyuan* | 0.0619178 | 19.9189 | 2.30E-16 | 0.0485848 | 99573.9 | 87962.1 |  |
| *A.m.unicolor* | *A.m.capensis* | *A.m.syriaca* | 0.0663639 | 19.9145 | 2.30E-16 | 0.0705821 | 104050 | 91099.4 |  |
| *A.m.capensis* | *A.m.intermissa* | *A.m.carnica* | 0.0561987 | 19.8831 | 2.30E-16 | 0.0535686 | 96178.8 | 85943.8 |  |
| *XJblackbee* | *A.m.pomonella* | *A.m.jemenitica* | 0.0681356 | 19.7761 | 2.30E-16 | 0.105205 | 72878.2 | 63580.5 |  |
| *A.m.caucasica* | *A.m.syriaca* | *A.m.iberiensis* | 0.062598 | 19.7266 | 2.30E-16 | 0.0215912 | 57701.8 | 50903.3 |  |
| *A.m.unicolor* | *A.m.jemenitica* | *A.m.sinisxinyuan* | 0.061224 | 19.6556 | 2.30E-16 | 0.0485209 | 100507 | 88910.2 |  |
| *A.m.carnica* | *A.m.iberiensis* | *A.m.unicolor* | 0.107396 | 19.6207 | 2.30E-16 | 0.0659445 | 100702 | 81169.7 |  |
| *XJblackbee* | *A.m.caucasica* | *A.m.unicolor* | 0.0816306 | 19.5505 | 2.30E-16 | 0.0379416 | 73518 | 62421.2 |  |
| *A.m.intermissa* | *A.m.jemenitica* | *A.m.syriaca* | 0.0613129 | 19.5069 | 2.30E-16 | 0.0677091 | 105102 | 92958.2 |  |
| *XJblackbee* | *A.m.pomonella* | *A.m.monticola* | 0.067745 | 19.4768 | 2.30E-16 | 0.0936718 | 71906.1 | 62781.7 |  |
| *A.m.carnica* | *A.m.iberiensis* | *A.m.lamarckiiA* | 0.10617 | 19.3944 | 2.30E-16 | 0.191734 | 100967 | 81585.2 |  |
| *A.m.ligustica* | *A.m.pomonella* | *A.m.capensis* | 0.0864413 | 19.3106 | 2.30E-16 | 0.122409 | 76273.4 | 64136.2 |  |
| *A.m.anatoliaca* | *A.m.pomonella* | *A.m.intermissa* | 0.0648184 | 19.283 | 2.30E-16 | 0.0592809 | 51066.3 | 44849.2 |  |
| *A.m.carnica* | *A.m.sinisxinyuan* | *A.m.monticola* | 0.0860751 | 19.2198 | 2.30E-16 | 0.127266 | 81516.9 | 68595.9 |  |
| *A.m.unicolor* | *A.m.capensis* | *A.m.mellifera* | 0.0603525 | 19.1787 | 2.30E-16 | 0.0429761 | 101552 | 89992 |  |
| *XJblackbee* | *A.m.pomonella* | *A.m.lamarckiiA* | 0.0673418 | 19.0873 | 2.30E-16 | 0.0940162 | 72141.5 | 63038.3 |  |
| *A.m.lamarckiiA* | *A.m.lamarckii* | *A.m.sinisxinyuan* | 0.0493763 | 19.0462 | 2.30E-16 | 0.0392519 | 95962.4 | 86931.8 |  |
| *A.m.ligustica* | *A.m.iberiensis* | *A.m.monticola* | 0.101181 | 19.0287 | 2.30E-16 | 0.200262 | 109581 | 89443.7 |  |
| *XJblackbee* | *A.m.pomonella* | *A.m.scutellata* | 0.0665705 | 19.0228 | 2.30E-16 | 0.150383 | 71998.8 | 63011.1 |  |
| *A.m.monticola* | *A.m.capensis* | *A.m.carnica* | 0.0643226 | 18.9883 | 2.30E-16 | 0.0555617 | 92985.5 | 81746.3 |  |
| *A.m.unicolor* | *A.m.intermissa* | *A.m.pomonella* | 0.070856 | 18.8966 | 2.30E-16 | 0.0631509 | 107694 | 93442.3 |  |
| *A.m.unicolor* | *A.m.lamarckii* | *A.m.iberiensis* | 0.0671299 | 18.8478 | 2.30E-16 | 0.042684 | 105093 | 91871 |  |
| *XJblackbee* | *A.m.pomonella* | *A.m.unicolor* | 0.0677601 | 18.7935 | 2.30E-16 | 0.0312238 | 72007.5 | 62868.3 |  |
| *A.m.carnica* | *A.m.ligustica* | *A.m.syriaca* | 0.087926 | 18.6361 | 2.30E-16 | 0.0662601 | 53831.4 | 45130.1 |  |
| *A.m.unicolor* | *A.m.intermissa* | *A.m.ligustica* | 0.0793829 | 18.6229 | 2.30E-16 | 0.0561909 | 104019 | 88719 |  |
| *A.m.unicolor* | *A.m.capensis* | *A.m.iberiensis* | 0.0590183 | 18.5634 | 2.30E-16 | 0.036963 | 102732 | 91281.7 |  |
| *XJblackbee* | *A.m.caucasica* | *A.m.adansonii* | 0.0750119 | 18.5072 | 2.30E-16 | 0.114856 | 73106.1 | 62903.8 |  |
| *A.m.anatoliaca* | *A.m.pomonella* | *A.m.carnica* | 0.0738378 | 18.4919 | 2.30E-16 | 0.0775463 | 59746.4 | 51530 |  |
| *A.m.ligustica* | *A.m.carnica* | *A.m.intermissa* | 0.0708208 | 18.3699 | 2.30E-16 | 0.0600715 | 49913.9 | 43311.6 |  |
| *A.m.scutellata* | *A.m.lamarckii* | *A.m.sinisxinyuan* | 0.0531786 | 18.3047 | 2.30E-16 | 0.0424087 | 96943.4 | 87153.4 |  |
| *A.m.jemenitica* | *A.m.lamarckiiA* | *A.m.monticola* | 0.0332778 | 18.2207 | 2.30E-16 | 0.133635 | 106577 | 99712.4 |  |
| *A.m.carnica* | *A.m.mellifera* | *A.m.scutellata* | 0.0958989 | 18.2126 | 2.30E-16 | 0.253207 | 93149.4 | 76847 |  |
| *A.m.monticola* | *A.m.capensis* | *XJblackbee* | 0.0652981 | 18.1559 | 2.30E-16 | 0.0601727 | 95465.2 | 83762 |  |
| *A.m.intermissa* | *A.m.adansonii* | *A.m.lamarckii* | 0.0317398 | 18.107 | 2.30E-16 | 0.0427918 | 102932 | 96598.7 |  |
| *XJblackbee* | *A.m.pomonella* | *A.m.capensis* | 0.0650612 | 17.9853 | 2.30E-16 | 0.091635 | 71890 | 63106.9 |  |
| *A.m.monticola* | *A.m.capensis* | *A.m.pomonella* | 0.0611391 | 17.9826 | 2.30E-16 | 0.0506062 | 98528 | 87174.3 |  |
| *A.m.monticola* | *A.m.capensis* | *A.m.ligustica* | 0.0665024 | 17.924 | 2.30E-16 | 0.0434795 | 94478.6 | 82696 |  |
| *A.m.ligustica* | *A.m.pomonella* | *A.m.intermissa* | 0.0782531 | 17.893 | 2.30E-16 | 0.104812 | 79560.6 | 68012.6 |  |
| *A.m.ligustica* | *XJblackbee* | *A.m.intermissa* | 0.0583318 | 17.8833 | 2.30E-16 | 0.0464309 | 46423.8 | 41306.4 |  |
| *A.m.mellifera* | *A.m.iberiensis* | *A.m.monticola* | 0.046862 | 17.8544 | 2.30E-16 | 0.0413338 | 38753.5 | 35283.9 |  |
| *A.m.carnica* | *A.m.mellifera* | *A.m.unicolor* | 0.0962883 | 17.8265 | 2.30E-16 | 0.0550361 | 92884.6 | 76568.3 |  |
| *A.m.unicolor* | *A.m.lamarckiiA* | *A.m.carnica* | 0.0543097 | 17.8142 | 2.30E-16 | 0.0457568 | 90548.5 | 81219.8 |  |
| *A.m.pomonella* | *A.m.iberiensis* | *A.m.adansonii* | 0.0832177 | 17.7986 | 2.30E-16 | 0.212606 | 109241 | 92456 |  |
| *A.m.syriaca* | *A.m.ligustica* | *A.m.iberiensis* | 0.0878395 | 17.764 | 2.30E-16 | 0.0491451 | 93774.5 | 78630.5 |  |
| *A.m.scutellata* | *A.m.jemenitica* | *A.m.anatoliaca* | 0.0414576 | 17.7298 | 2.30E-16 | 0.0304521 | 96501.2 | 88818.3 |  |
| *A.m.capensis* | *A.m.lamarckii* | *A.m.sinisxinyuan* | 0.0360243 | 17.6842 | 2.30E-16 | 0.0285779 | 93514.6 | 87011.2 |  |
| *A.m.scutellata* | *A.m.jemenitica* | *A.m.syriaca* | 0.0418032 | 17.6297 | 2.30E-16 | 0.0458672 | 100167 | 92128.1 |  |
| *A.m.unicolor* | *A.m.capensis* | *A.m.intermissa* | 0.0560124 | 17.5935 | 2.30E-16 | 0.135815 | 109176 | 97594.7 |  |
| *A.m.carnica* | *A.m.mellifera* | *A.m.lamarckiiA* | 0.0949171 | 17.5818 | 2.30E-16 | 0.159451 | 93132.2 | 76985.1 |  |
| *A.m.carnica* | *A.m.ligustica* | *A.m.pomonella* | 0.0751697 | 17.5715 | 2.30E-16 | 0.0611834 | 58082.7 | 49961.1 |  |
| *A.m.monticola* | *A.m.capensis* | *A.m.anatoliaca* | 0.0645767 | 17.5391 | 2.30E-16 | 0.0465522 | 99530 | 87455.2 |  |
| *A.m.scutellata* | *A.m.jemenitica* | *A.m.pomonella* | 0.0382788 | 17.5316 | 2.30E-16 | 0.0323442 | 95440.9 | 88403.6 |  |
| *A.m.carnica* | *A.m.iberiensis* | *A.m.capensis* | 0.09893 | 17.5286 | 2.30E-16 | 0.179678 | 100175 | 82139 |  |
| *A.m.sinisxinyuan* | *A.m.syriaca* | *A.m.capensis* | 0.0837423 | 17.4677 | 2.30E-16 | 0.183064 | 106980 | 90446.9 |  |
| *A.m.monticola* | *A.m.capensis* | *A.m.caucasica* | 0.064532 | 17.4658 | 2.30E-16 | 0.0403348 | 99304 | 87264.4 |  |
| *A.m.scutellata* | *A.m.jemenitica* | *A.m.caucasica* | 0.0418092 | 17.4352 | 2.30E-16 | 0.0265295 | 96335.3 | 88603.1 |  |
| *A.m.jemenitica* | *A.m.lamarckii* | *A.m.sinisxinyuan* | 0.0358954 | 17.4165 | 2.30E-16 | 0.0286811 | 94185.3 | 87658 |  |
| *A.m.monticola* | *A.m.intermissa* | *A.m.ligustica* | 0.0730121 | 17.3956 | 2.30E-16 | 0.0510655 | 101688 | 87849.1 |  |
| *A.m.unicolor* | *A.m.lamarckiiA* | *XJblackbee* | 0.0527682 | 17.3442 | 2.30E-16 | 0.0475007 | 92795.5 | 83493.1 |  |
| *A.m.sinisxinyuan* | *A.m.iberiensis* | *A.m.jemenitica* | 0.0615694 | 17.2621 | 2.30E-16 | 0.0745926 | 54530.4 | 48205 |  |
| *A.m.syriaca* | *A.m.capensis* | *A.m.iberiensis* | 0.0494614 | 17.2558 | 2.30E-16 | 0.0315386 | 103123 | 93403 |  |
| *A.m.monticola* | *A.m.intermissa* | *A.m.pomonella* | 0.0648504 | 17.2419 | 2.30E-16 | 0.0571352 | 105239 | 92420.6 |  |
| *A.m.intermissa* | *A.m.lamarckii* | *XJblackbee* | 0.0570548 | 17.1666 | 2.30E-16 | 0.0607674 | 100746 | 89870.1 |  |
| *A.m.sinisxinyuan* | *A.m.syriaca* | *A.m.lamarckiiA* | 0.079465 | 17.1193 | 2.30E-16 | 0.174658 | 106757 | 91039.4 |  |
| *A.m.sinisxinyuan* | *A.m.mellifera* | *A.m.monticola* | 0.0487929 | 17.1115 | 2.30E-16 | 0.0550987 | 52654.1 | 47754.9 |  |
| *A.m.carnica* | *A.m.ligustica* | *A.m.anatoliaca* | 0.0908342 | 17.091 | 2.30E-16 | 0.052552 | 56248.1 | 46880.5 |  |
| *A.m.unicolor* | *A.m.jemenitica* | *A.m.mellifera* | 0.0548411 | 17.0857 | 2.30E-16 | 0.0393934 | 101912 | 91315.1 |  |
| *A.m.unicolor* | *A.m.lamarckiiA* | *A.m.ligustica* | 0.053051 | 17.0393 | 2.30E-16 | 0.0339446 | 91731.5 | 82488.9 |  |
| *A.m.sinisxinyuan* | *A.m.syriaca* | *A.m.scutellata* | 0.0771443 | 17.0348 | 2.30E-16 | 0.288745 | 106396 | 91156.3 |  |
| *A.m.lamarckiiA* | *A.m.jemenitica* | *A.m.anatoliaca* | 0.0363319 | 17.0279 | 2.30E-16 | 0.0266104 | 95358.3 | 88672.1 |  |
| *A.m.ligustica* | *A.m.mellifera* | *A.m.monticola* | 0.0896762 | 16.9704 | 2.30E-16 | 0.165962 | 101589 | 84868.5 |  |
| *A.m.intermissa* | *A.m.jemenitica* | *A.m.anatoliaca* | 0.054946 | 16.857 | 2.30E-16 | 0.0411804 | 100849 | 90343.7 |  |
| *XJblackbee* | *A.m.iberiensis* | *A.m.monticola* | 0.0856849 | 16.8307 | 2.30E-16 | 0.171971 | 105896 | 89180.9 |  |
| *A.m.unicolor* | *A.m.lamarckiiA* | *A.m.sinisxinyuan* | 0.0498625 | 16.8263 | 2.30E-16 | 0.0381003 | 95862.2 | 86756.4 |  |
| *A.m.unicolor* | *A.m.lamarckiiA* | *A.m.jemenitica* | 0.0446017 | 16.8124 | 2.30E-16 | 0.161926 | 107266 | 98106.4 |  |
| *A.m.unicolor* | *A.m.lamarckiiA* | *A.m.pomonella* | 0.0505885 | 16.7603 | 2.30E-16 | 0.0410072 | 96089.9 | 86836 |  |
| *A.m.intermissa* | *A.m.jemenitica* | *A.m.caucasica* | 0.0551692 | 16.7037 | 2.30E-16 | 0.0357699 | 100646 | 90121.3 |  |
| *A.m.scutellata* | *A.m.adansonii* | *A.m.iberiensis* | 0.0604125 | 16.6663 | 2.30E-16 | 0.0387357 | 102517 | 90836.4 |  |
| *A.m.monticola* | *A.m.scutellata* | *A.m.carnica* | 0.038455 | 16.6353 | 2.30E-16 | 0.0329452 | 89984.7 | 83320.3 |  |
| *A.m.unicolor* | *A.m.lamarckiiA* | *A.m.anatoliaca* | 0.0511912 | 16.5738 | 2.30E-16 | 0.03618 | 96800 | 87372 |  |
| *A.m.carnica* | *A.m.sinisxinyuan* | *A.m.scutellata* | 0.0776567 | 16.5428 | 2.30E-16 | 0.181625 | 81018.7 | 69342.2 |  |
| *A.m.lamarckiiA* | *A.m.jemenitica* | *A.m.caucasica* | 0.0366421 | 16.5321 | 2.30E-16 | 0.0231716 | 95193.5 | 88463.9 |  |
| *A.m.ligustica* | *A.m.iberiensis* | *A.m.scutellata* | 0.0915325 | 16.527 | 2.30E-16 | 0.289631 | 108780 | 90536.2 |  |
| *A.m.caucasica* | *A.m.pomonella* | *A.m.ligustica* | 0.0939753 | 16.525 | 2.30E-16 | 0.0539301 | 52789.6 | 43720.1 |  |
| *A.m.scutellata* | *A.m.adansonii* | *A.m.mellifera* | 0.055279 | 16.4702 | 2.30E-16 | 0.0403955 | 100550 | 90016 |  |
| *A.m.unicolor* | *A.m.lamarckiiA* | *A.m.caucasica* | 0.0514418 | 16.454 | 2.30E-16 | 0.0315526 | 96632.4 | 87176.9 |  |
| *A.m.jemenitica* | *A.m.intermissa* | *A.m.carnica* | 0.0467027 | 16.4157 | 2.30E-16 | 0.0455866 | 96795.5 | 88157.7 |  |
| *A.m.sinisxinyuan* | *A.m.syriaca* | *A.m.unicolor* | 0.0769277 | 16.381 | 2.30E-16 | 0.0530502 | 106041 | 90891.3 |  |
| *A.m.unicolor* | *A.m.lamarckiiA* | *A.m.lamarckii* | 0.0509719 | 16.3765 | 2.30E-16 | 0.0707476 | 108272 | 97769.5 |  |
| *A.m.monticola* | *A.m.capensis* | *A.m.syriaca* | 0.0598198 | 16.3212 | 2.30E-16 | 0.0633307 | 102198 | 90660.8 |  |
| *A.m.unicolor* | *A.m.lamarckiiA* | *A.m.mellifera* | 0.0490362 | 16.2782 | 2.30E-16 | 0.0340152 | 97869.3 | 88719.7 |  |
| *XJblackbee* | *A.m.syriaca* | *A.m.intermissa* | 0.0587382 | 16.272 | 2.30E-16 | 0.0898406 | 85129.2 | 75683.3 |  |
| *A.m.unicolor* | *A.m.lamarckiiA* | *A.m.syriaca* | 0.0507979 | 16.2705 | 2.30E-16 | 0.0526001 | 99822.1 | 90170.8 |  |
| *A.m.lamarckiiA* | *A.m.jemenitica* | *A.m.pomonella* | 0.0329819 | 16.2596 | 2.30E-16 | 0.0278055 | 94290.9 | 88269.7 |  |
| *A.m.carnica* | *A.m.sinisxinyuan* | *A.m.unicolor* | 0.0782122 | 16.232 | 2.30E-16 | 0.0395323 | 80743 | 69029 |  |
| *A.m.mellifera* | *A.m.iberiensis* | *A.m.scutellata* | 0.045079 | 16.2288 | 2.30E-16 | 0.0695284 | 38780.9 | 35435.3 |  |
| *A.m.ligustica* | *A.m.iberiensis* | *A.m.unicolor* | 0.0917068 | 16.211 | 2.30E-16 | 0.0615602 | 108352 | 90147.9 |  |
| *A.m.monticola* | *A.m.lamarckiiA* | *A.m.carnica* | 0.0435459 | 16.1346 | 2.30E-16 | 0.0375176 | 90934.3 | 83345.1 |  |
| *A.m.ligustica* | *A.m.iberiensis* | *A.m.lamarckiiA* | 0.0910124 | 16.1217 | 2.30E-16 | 0.181372 | 108771 | 90623.3 |  |
| *A.m.scutellata* | *A.m.jemenitica* | *XJblackbee* | 0.037984 | 16.0564 | 2.30E-16 | 0.0358364 | 91847.9 | 85125.7 |  |
| *A.m.iberiensis* | *A.m.capensis* | *A.m.anatoliaca* | 0.0752244 | 16.0487 | 2.30E-16 | 0.0571599 | 107116 | 92127.6 |  |
| *A.m.iberiensis* | *A.m.intermissa* | *A.m.syriaca* | 0.0596976 | 16.0007 | 2.30E-16 | 0.0520235 | 87292.1 | 77457 |  |
| *A.m.adansonii* | *A.m.intermissa* | *A.m.pomonella* | 0.0288418 | 15.9962 | 2.30E-16 | 0.024303 | 93966.7 | 88698.3 |  |
| *A.m.iberiensis* | *A.m.adansonii* | *A.m.syriaca* | 0.0617879 | 15.9901 | 2.30E-16 | 0.0649262 | 105446 | 93173.9 |  |
| *A.m.lamarckiiA* | *A.m.adansonii* | *A.m.iberiensis* | 0.0566965 | 15.9841 | 2.30E-16 | 0.036413 | 102077 | 91122.9 |  |
| *A.m.monticola* | *A.m.scutellata* | *A.m.pomonella* | 0.0367984 | 15.9815 | 2.30E-16 | 0.0302193 | 95512 | 88732.1 |  |
| *A.m.carnica* | *A.m.anatoliaca* | *A.m.lamarckii* | 0.0634567 | 15.9795 | 2.30E-16 | 0.0564906 | 81604.5 | 71865.8 |  |
| *A.m.monticola* | *A.m.scutellata* | *XJblackbee* | 0.0389616 | 15.9773 | 2.30E-16 | 0.0356105 | 92349.1 | 85422.8 |  |
| *A.m.lamarckiiA* | *A.m.jemenitica* | *A.m.syriaca* | 0.0355793 | 15.9474 | 2.30E-16 | 0.0390636 | 98916.6 | 92119.7 |  |
| *A.m.sinisxinyuan* | *A.m.syriaca* | *A.m.monticola* | 0.0695253 | 15.9366 | 2.30E-16 | 0.154275 | 105517 | 91798.3 |  |
| *A.m.scutellata* | *A.m.jemenitica* | *A.m.carnica* | 0.0358345 | 15.8654 | 2.30E-16 | 0.0315508 | 89212.7 | 83040.1 |  |
| *A.m.intermissa* | *A.m.lamarckii* | *A.m.unicolor* | 0.0496703 | 15.8234 | 2.30E-16 | 0.0404379 | 109405 | 99050.8 |  |
| *A.m.carnica* | *A.m.sinisxinyuan* | *A.m.lamarckiiA* | 0.0764002 | 15.8214 | 2.30E-16 | 0.113643 | 80986.1 | 69489.7 |  |
| *A.m.carnica* | *A.m.mellifera* | *A.m.capensis* | 0.0878364 | 15.8137 | 2.30E-16 | 0.148395 | 92398.6 | 77477.3 |  |
| *A.m.sinisxinyuan* | *A.m.mellifera* | *A.m.scutellata* | 0.0459978 | 15.812 | 2.30E-16 | 0.0876048 | 52568.8 | 47945.3 |  |
| *A.m.unicolor* | *A.m.lamarckiiA* | *A.m.iberiensis* | 0.0484683 | 15.7826 | 2.30E-16 | 0.0295774 | 99100 | 89937.7 |  |
| *A.m.unicolor* | *A.m.jemenitica* | *A.m.iberiensis* | 0.0513694 | 15.7557 | 2.30E-16 | 0.0324262 | 102796 | 92751.3 |  |
| *A.m.iberiensis* | *A.m.capensis* | *A.m.caucasica* | 0.0742901 | 15.7352 | 2.30E-16 | 0.0490557 | 106796 | 92025.2 |  |
| *A.m.lamarckiiA* | *A.m.adansonii* | *A.m.mellifera* | 0.0512793 | 15.6958 | 2.30E-16 | 0.0375613 | 100098 | 90332.9 |  |
| *A.m.jemenitica* | *A.m.adansonii* | *A.m.iberiensis* | 0.0520414 | 15.6696 | 2.30E-16 | 0.0334973 | 101543 | 91496.8 |  |
| *A.m.monticola* | *A.m.scutellata* | *A.m.anatoliaca* | 0.0383634 | 15.6369 | 2.30E-16 | 0.0274344 | 96306.4 | 89190.1 |  |
| *A.m.monticola* | *A.m.scutellata* | *A.m.ligustica* | 0.0394861 | 15.625 | 2.30E-16 | 0.0255993 | 91315.6 | 84378.1 |  |
| *A.m.scutellata* | *A.m.jemenitica* | *A.m.ligustica* | 0.0386662 | 15.5574 | 2.30E-16 | 0.0255997 | 90794 | 84034 |  |
| *A.m.carnica* | *A.m.ligustica* | *A.m.caucasica* | 0.0840256 | 15.5304 | 2.30E-16 | 0.0395052 | 55585.6 | 46968.4 |  |
| *A.m.unicolor* | *A.m.lamarckiiA* | *A.m.adansonii* | 0.0458625 | 15.511 | 2.30E-16 | 0.177616 | 107565 | 98131.3 |  |
| *A.m.adansonii* | *A.m.jemenitica* | *A.m.syriaca* | 0.0501361 | 15.499 | 2.30E-16 | 0.0549501 | 101797 | 92076.7 |  |
| *A.m.monticola* | *A.m.scutellata* | *A.m.caucasica* | 0.0381377 | 15.4839 | 2.30E-16 | 0.0236491 | 96080.1 | 89020.7 |  |
| *A.m.ligustica* | *XJblackbee* | *A.m.adansonii* | 0.0477095 | 15.4832 | 2.30E-16 | 0.0410201 | 41764.9 | 37961.2 |  |
| *A.m.capensis* | *A.m.lamarckii* | *A.m.anatoliaca* | 0.0392759 | 15.4743 | 2.30E-16 | 0.0290355 | 95008.7 | 87827.6 |  |
| *A.m.monticola* | *A.m.lamarckiiA* | *A.m.pomonella* | 0.0420655 | 15.4353 | 2.30E-16 | 0.0347309 | 96509.1 | 88717.4 |  |
| *A.m.unicolor* | *A.m.lamarckiiA* | *A.m.intermissa* | 0.046821 | 15.4281 | 2.30E-16 | 0.110844 | 105668 | 96216 |  |
| *A.m.scutellata* | *A.m.adansonii* | *A.m.sinisxinyuan* | 0.0473048 | 15.4239 | 2.30E-16 | 0.0380998 | 97359.6 | 88564.5 |  |
| *A.m.monticola* | *A.m.lamarckii* | *A.m.mellifera* | 0.0598966 | 15.3755 | 2.30E-16 | 0.0435889 | 102558 | 90966.7 |  |
| *A.m.carnica* | *A.m.iberiensis* | *A.m.jemenitica* | 0.0823384 | 15.3404 | 2.30E-16 | 0.161291 | 98890.4 | 83844.3 |  |
| *A.m.unicolor* | *A.m.scutellata* | *A.m.carnica* | 0.0484495 | 15.3356 | 2.30E-16 | 0.0413199 | 91154.9 | 82730.2 |  |
| *A.m.lamarckiiA* | *A.m.jemenitica* | *XJblackbee* | 0.0331696 | 15.3309 | 2.30E-16 | 0.0312222 | 90771.4 | 84943 |  |
| *A.m.carnica* | *XJblackbee* | *A.m.adansonii* | 0.0361047 | 15.225 | 2.30E-16 | 0.0380123 | 50271.4 | 46767.9 |  |
| *A.m.syriaca* | *A.m.jemenitica* | *A.m.iberiensis* | 0.0427499 | 15.2236 | 2.30E-16 | 0.0270023 | 101503 | 93180.7 |  |
| *A.m.sinisxinyuan* | *A.m.mellifera* | *A.m.lamarckiiA* | 0.046212 | 15.1753 | 2.30E-16 | 0.0516721 | 52629.9 | 47980.5 |  |
| *A.m.mellifera* | *A.m.iberiensis* | *A.m.lamarckiiA* | 0.0443976 | 15.1281 | 2.30E-16 | 0.0386845 | 38807.9 | 35508.4 |  |
| *A.m.carnica* | *A.m.pomonella* | *A.m.lamarckii* | 0.0516438 | 15.1172 | 2.30E-16 | 0.0446108 | 78353.1 | 70657.6 |  |
| *A.m.sinisxinyuan* | *A.m.mellifera* | *A.m.capensis* | 0.0453838 | 14.9688 | 2.30E-16 | 0.0504495 | 52465.6 | 47910.1 |  |
| *A.m.monticola* | *A.m.lamarckiiA* | *XJblackbee* | 0.043742 | 14.9572 | 2.30E-16 | 0.040199 | 93279.4 | 85461 |  |
| *A.m.lamarckiiA* | *A.m.jemenitica* | *A.m.ligustica* | 0.034098 | 14.9151 | 2.30E-16 | 0.0224863 | 89738.3 | 83820.3 |  |
| *A.m.capensis* | *A.m.lamarckii* | *A.m.caucasica* | 0.0389081 | 14.9 | 2.30E-16 | 0.0247765 | 94757.5 | 87660 |  |
| *A.m.lamarckii* | *A.m.lamarckiiA* | *A.m.monticola* | 0.0255398 | 14.8052 | 2.30E-16 | 0.105557 | 105411 | 100161 |  |
| *A.m.monticola* | *A.m.scutellata* | *A.m.syriaca* | 0.036342 | 14.786 | 2.30E-16 | 0.0381967 | 99218.7 | 92260 |  |
| *A.m.mellifera* | *A.m.iberiensis* | *A.m.unicolor* | 0.0443427 | 14.7183 | 2.30E-16 | 0.011706 | 38718.3 | 35430.4 |  |
| *A.m.ligustica* | *XJblackbee* | *A.m.iberiensis* | 0.0809048 | 14.6974 | 2.30E-16 | 0.0301703 | 59068.8 | 50226.3 |  |
| *A.m.ligustica* | *A.m.sinisxinyuan* | *A.m.monticola* | 0.0738439 | 14.6531 | 2.30E-16 | 0.116854 | 85533.6 | 73770 |  |
| *A.m.jemenitica* | *A.m.adansonii* | *A.m.mellifera* | 0.0435588 | 14.6522 | 2.30E-16 | 0.0320698 | 99309.5 | 91019 |  |
| *A.m.unicolor* | *A.m.scutellata* | *XJblackbee* | 0.0472437 | 14.6404 | 2.30E-16 | 0.0430468 | 93440.2 | 85009.6 |  |
| *A.m.monticola* | *A.m.lamarckiiA* | *A.m.anatoliaca* | 0.043492 | 14.6202 | 2.30E-16 | 0.0312622 | 97276.2 | 89167.4 |  |
| *A.m.jemenitica* | *A.m.lamarckii* | *A.m.pomonella* | 0.0309071 | 14.62 | 2.30E-16 | 0.0270439 | 94953.4 | 89259.9 |  |
| *A.m.ligustica* | *XJblackbee* | *A.m.monticola* | 0.0431961 | 14.6121 | 2.30E-16 | 0.0340132 | 41408.1 | 37978.9 |  |
| *A.m.unicolor* | *A.m.adansonii* | *A.m.carnica* | 0.0525468 | 14.5735 | 2.30E-16 | 0.0469132 | 95787.2 | 86223.2 |  |
| *A.m.ligustica* | *A.m.mellifera* | *A.m.scutellata* | 0.0800854 | 14.5522 | 2.30E-16 | 0.236771 | 100817 | 85866.7 |  |
| *A.m.sinisxinyuan* | *A.m.mellifera* | *A.m.unicolor* | 0.0456237 | 14.5363 | 2.30E-16 | 0.0160486 | 52510.1 | 47927.7 |  |
| *A.m.lamarckiiA* | *A.m.adansonii* | *A.m.sinisxinyuan* | 0.0433098 | 14.5271 | 2.30E-16 | 0.0349721 | 96913.8 | 88867.7 |  |
| *A.m.monticola* | *A.m.lamarckiiA* | *A.m.caucasica* | 0.0433073 | 14.5206 | 2.30E-16 | 0.0269947 | 97056.7 | 88999.1 |  |
| *A.m.lamarckiiA* | *A.m.jemenitica* | *A.m.carnica* | 0.030693 | 14.4978 | 2.30E-16 | 0.0269576 | 88125.9 | 82877.3 |  |
| *A.m.monticola* | *A.m.lamarckiiA* | *A.m.ligustica* | 0.0440351 | 14.4841 | 2.30E-16 | 0.0287061 | 92219.2 | 84440 |  |
| *A.m.unicolor* | *A.m.scutellata* | *A.m.ligustica* | 0.0477535 | 14.4619 | 2.30E-16 | 0.0309314 | 92398.1 | 83975.7 |  |
| *XJblackbee* | *A.m.mellifera* | *A.m.monticola* | 0.0723496 | 14.441 | 2.30E-16 | 0.136445 | 98479.1 | 85190.7 |  |
| *A.m.monticola* | *A.m.lamarckiiA* | *A.m.syriaca* | 0.0426207 | 14.3364 | 2.30E-16 | 0.0450396 | 100358 | 92153.3 |  |
| *A.m.mellifera* | *A.m.iberiensis* | *A.m.capensis* | 0.0429051 | 14.3283 | 2.30E-16 | 0.0371059 | 38658.8 | 35478 |  |
| *A.m.ligustica* | *A.m.mellifera* | *A.m.unicolor* | 0.080482 | 14.2864 | 2.30E-16 | 0.0505548 | 100438 | 85475.4 |  |
| *XJblackbee* | *A.m.iberiensis* | *A.m.scutellata* | 0.0758781 | 14.2576 | 2.30E-16 | 0.248791 | 105114 | 90287 |  |
| *A.m.intermissa* | *A.m.sinisxinyuan* | *A.m.syriaca* | 0.055325 | 14.2048 | 2.30E-16 | 0.0576331 | 98537.4 | 88205.8 |  |
| *A.m.carnica* | *A.m.caucasica* | *A.m.lamarckii* | 0.0599619 | 14.2041 | 2.30E-16 | 0.0535159 | 81510.7 | 72288.6 |  |
| *A.m.ligustica* | *A.m.mellifera* | *A.m.lamarckiiA* | 0.0797132 | 14.1702 | 2.30E-16 | 0.148563 | 100841 | 85951.6 |  |
| *A.m.unicolor* | *A.m.scutellata* | *A.m.pomonella* | 0.0446429 | 14.168 | 2.30E-16 | 0.0366054 | 96655 | 88393.9 |  |
| *A.m.ligustica* | *A.m.iberiensis* | *A.m.capensis* | 0.0826062 | 14.1648 | 2.30E-16 | 0.166252 | 107814 | 91360.7 |  |
| *A.m.unicolor* | *A.m.scutellata* | *A.m.sinisxinyuan* | 0.0453192 | 14.088 | 2.30E-16 | 0.0350287 | 96555.7 | 88183.5 |  |
| *A.m.adansonii* | *A.m.jemenitica* | *A.m.anatoliaca* | 0.0468734 | 14.0531 | 2.30E-16 | 0.034581 | 97837.2 | 89076 |  |
| *A.m.adansonii* | *A.m.jemenitica* | *A.m.caucasica* | 0.0472349 | 14.0183 | 2.30E-16 | 0.0301179 | 97656.7 | 88847.2 |  |
| *XJblackbee* | *A.m.iberiensis* | *A.m.unicolor* | 0.076024 | 13.9805 | 2.30E-16 | 0.0506106 | 104726 | 89927.4 |  |
| *A.m.ligustica* | *XJblackbee* | *A.m.scutellata* | 0.0430136 | 13.9601 | 2.30E-16 | 0.0541454 | 41490.6 | 38068.5 |  |
| *A.m.pomonella* | *A.m.mellifera* | *A.m.adansonii* | 0.0635645 | 13.9385 | 2.30E-16 | 0.155741 | 103082 | 90760.1 |  |
| *A.m.monticola* | *A.m.capensis* | *A.m.sinisxinyuan* | 0.0469501 | 13.9089 | 2.30E-16 | 0.0369303 | 97304.7 | 88577.5 |  |
| *A.m.capensis* | *A.m.lamarckiiA* | *A.m.unicolor* | 0.0362835 | 13.8791 | 2.30E-16 | 0.0307569 | 106379 | 98929.7 |  |
| *A.m.unicolor* | *A.m.scutellata* | *A.m.anatoliaca* | 0.0453509 | 13.8703 | 2.30E-16 | 0.0324278 | 97394.8 | 88944.1 |  |
| *A.m.ligustica* | *XJblackbee* | *A.m.lamarckiiA* | 0.0436703 | 13.8611 | 2.30E-16 | 0.0346756 | 41557.6 | 38079.8 |  |
| *XJblackbee* | *A.m.iberiensis* | *A.m.lamarckiiA* | 0.0750463 | 13.8126 | 2.30E-16 | 0.151794 | 105065 | 90396 |  |
| *A.m.ligustica* | *XJblackbee* | *A.m.jemenitica* | 0.0421969 | 13.8117 | 2.30E-16 | 0.0368561 | 41756.8 | 38375.4 |  |
| *A.m.unicolor* | *A.m.scutellata* | *A.m.caucasica* | 0.0455707 | 13.792 | 2.30E-16 | 0.0282794 | 97225.2 | 88750.2 |  |
| *A.m.carnica* | *A.m.sinisxinyuan* | *A.m.capensis* | 0.0689756 | 13.7587 | 2.30E-16 | 0.103203 | 80336.8 | 69969.3 |  |
| *A.m.carnica* | *A.m.mellifera* | *A.m.jemenitica* | 0.0726351 | 13.7427 | 2.30E-16 | 0.132385 | 91369.2 | 78994.8 |  |
| *A.m.monticola* | *A.m.jemenitica* | *A.m.sinisxinyuan* | 0.0460649 | 13.7224 | 2.30E-16 | 0.0368253 | 98809.3 | 90106.9 |  |
| *A.m.capensis* | *A.m.adansonii* | *A.m.iberiensis* | 0.0452903 | 13.7117 | 2.30E-16 | 0.02892 | 99621.3 | 90988.6 |  |
| *A.m.monticola* | *A.m.scutellata* | *A.m.jemenitica* | 0.0320239 | 13.6776 | 2.30E-16 | 0.118183 | 106642 | 100024 |  |
| *A.m.unicolor* | *A.m.scutellata* | *A.m.mellifera* | 0.0444811 | 13.4578 | 2.30E-16 | 0.0312121 | 98576.2 | 90180.1 |  |
| *A.m.ligustica* | *XJblackbee* | *A.m.mellifera* | 0.0651967 | 13.3384 | 2.30E-16 | 0.0313786 | 59439.7 | 52163.6 |  |
| *A.m.unicolor* | *A.m.scutellata* | *A.m.syriaca* | 0.0438598 | 13.3364 | 2.30E-16 | 0.0459175 | 100265 | 91839.3 |  |
| *A.m.jemenitica* | *A.m.capensis* | *A.m.monticola* | 0.0259998 | 13.3267 | 2.30E-16 | 0.102991 | 104411 | 99119 |  |
| *A.m.ligustica* | *XJblackbee* | *A.m.unicolor* | 0.0428684 | 13.3172 | 2.30E-16 | 0.0115136 | 41464.4 | 38055.5 |  |
| *A.m.unicolor* | *A.m.scutellata* | *A.m.adansonii* | 0.04388 | 13.3167 | 2.30E-16 | 0.171848 | 108589 | 99459.9 |  |
| *A.m.scutellata* | *A.m.intermissa* | *XJblackbee* | 0.0458044 | 13.2873 | 2.30E-16 | 0.0457947 | 98058.9 | 89469.2 |  |
| *A.m.capensis* | *A.m.adansonii* | *A.m.mellifera* | 0.0390077 | 13.2767 | 2.30E-16 | 0.0284436 | 97571.3 | 90245 |  |
| *A.m.mellifera* | *A.m.iberiensis* | *A.m.jemenitica* | 0.0371232 | 13.2682 | 2.30E-16 | 0.0338124 | 38391.7 | 35643.3 |  |
| *A.m.ligustica* | *XJblackbee* | *A.m.capensis* | 0.0421727 | 13.0478 | 2.30E-16 | 0.0338006 | 41428.6 | 38075.7 |  |
| *A.m.unicolor* | *A.m.scutellata* | *A.m.iberiensis* | 0.0441685 | 12.9801 | 2.30E-16 | 0.0272681 | 99849.9 | 91402.6 |  |
| *XJblackbee* | *A.m.pomonella* | *A.m.intermissa* | 0.0450621 | 12.9606 | 2.30E-16 | 0.0611879 | 74601 | 68167.5 |  |
| *A.m.unicolor* | *A.m.scutellata* | *A.m.intermissa* | 0.0437165 | 12.938 | 2.30E-16 | 0.104648 | 106539 | 97614.4 |  |
| *A.m.monticola* | *A.m.scutellata* | *A.m.lamarckii* | 0.0315118 | 12.9329 | 2.30E-16 | 0.0446074 | 106966 | 100430 |  |
| *A.m.adansonii* | *A.m.jemenitica* | *A.m.pomonella* | 0.0338508 | 12.9208 | 2.30E-16 | 0.028984 | 95953.4 | 89669.9 |  |
| *A.m.intermissa* | *A.m.capensis* | *A.m.syriaca* | 0.044888 | 12.7283 | 2.30E-16 | 0.0484306 | 101091 | 92405.4 |  |
| *A.m.lamarckii* | *A.m.scutellata* | *A.m.unicolor* | 0.0387004 | 12.6455 | 2.30E-16 | 0.0326368 | 107617 | 99597.5 |  |
| *A.m.monticola* | *A.m.scutellata* | *A.m.sinisxinyuan* | 0.0294848 | 12.5919 | 2.30E-16 | 0.0230184 | 94965.7 | 89526 |  |
| *A.m.monticola* | *A.m.capensis* | *A.m.mellifera* | 0.0440279 | 12.5835 | 2.30E-16 | 0.0314561 | 99182.8 | 90817.5 |  |
| *A.m.unicolor* | *A.m.adansonii* | *A.m.pomonella* | 0.0464181 | 12.5686 | 2.30E-16 | 0.0397125 | 101013 | 92051.1 |  |
| *A.m.intermissa* | *A.m.lamarckii* | *A.m.pomonella* | 0.0346501 | 12.4779 | 2.30E-16 | 0.0316204 | 99852.3 | 93164.3 |  |
| *A.m.adansonii* | *A.m.jemenitica* | *XJblackbee* | 0.0383378 | 12.403 | 2.30E-16 | 0.0365005 | 92773 | 85922.3 |  |
| *A.m.monticola* | *A.m.lamarckii* | *A.m.iberiensis* | 0.0504481 | 12.3982 | 2.30E-16 | 0.0321728 | 102689 | 92825.8 |  |
| *A.m.jemenitica* | *A.m.lamarckii* | *A.m.syriaca* | 0.0321977 | 12.394 | 2.30E-16 | 0.0373062 | 99984.6 | 93746.9 |  |
| *A.m.carnica* | *A.m.syriaca* | *A.m.intermissa* | 0.0473053 | 12.3418 | 2.30E-16 | 0.078376 | 89660.1 | 81560.5 |  |
| *A.m.iberiensis* | *A.m.intermissa* | *A.m.anatoliaca* | 0.0450114 | 12.3379 | 2.30E-16 | 0.0275002 | 83708.5 | 76497.4 |  |
| *A.m.sinisxinyuan* | *A.m.mellifera* | *A.m.jemenitica* | 0.0358745 | 12.3299 | 2.30E-16 | 0.0424527 | 52046.1 | 48441.2 |  |
| *A.m.ligustica* | *A.m.mellifera* | *A.m.capensis* | 0.0714006 | 12.3135 | 2.30E-16 | 0.134372 | 99937.8 | 86617.6 |  |
| *A.m.iberiensis* | *A.m.adansonii* | *A.m.anatoliaca* | 0.0464803 | 12.3088 | 2.30E-16 | 0.0342045 | 100961 | 91992.3 |  |
| *A.m.ligustica* | *A.m.sinisxinyuan* | *A.m.scutellata* | 0.0643951 | 12.2427 | 2.30E-16 | 0.162963 | 84938.9 | 74661.4 |  |
| *A.m.adansonii* | *A.m.jemenitica* | *A.m.ligustica* | 0.0411145 | 12.2312 | 2.30E-16 | 0.0274225 | 91848.8 | 84594.4 |  |
| *A.m.unicolor* | *A.m.scutellata* | *A.m.jemenitica* | 0.0351156 | 12.2282 | 2.30E-16 | 0.12867 | 107295 | 100015 |  |
| *A.m.carnica* | *A.m.sinisxinyuan* | *A.m.jemenitica* | 0.0580687 | 12.2072 | 2.30E-16 | 0.0938427 | 79836 | 71072.9 |  |
| *A.m.carnica* | *A.m.ligustica* | *A.m.lamarckii* | 0.0459383 | 12.1729 | 2.30E-16 | 0.0231746 | 45502.3 | 41505.3 |  |
| *A.m.monticola* | *A.m.adansonii* | *A.m.carnica* | 0.0440177 | 12.1128 | 2.30E-16 | 0.0388167 | 93109 | 85257.7 |  |
| *A.m.lamarckiiA* | *A.m.intermissa* | *XJblackbee* | 0.0411557 | 12.1038 | 2.30E-16 | 0.0412332 | 97360.8 | 89663.6 |  |
| *A.m.ligustica* | *A.m.sinisxinyuan* | *A.m.unicolor* | 0.064989 | 12.0704 | 2.30E-16 | 0.034884 | 84560.3 | 74240 |  |
| *XJblackbee* | *A.m.mellifera* | *A.m.scutellata* | 0.0626491 | 11.9707 | 2.30E-16 | 0.192872 | 97725.7 | 86202.7 |  |
| *A.m.monticola* | *A.m.lamarckiiA* | *A.m.sinisxinyuan* | 0.0333962 | 11.9518 | 2.30E-16 | 0.0262231 | 95875.6 | 89678.8 |  |
| *XJblackbee* | *A.m.iberiensis* | *A.m.capensis* | 0.0670708 | 11.9463 | 2.30E-16 | 0.136924 | 104190 | 91092.6 |  |
| *A.m.iberiensis* | *A.m.adansonii* | *A.m.caucasica* | 0.0453461 | 11.912 | 2.30E-16 | 0.0289983 | 100635 | 91904.5 |  |
| *A.m.anatoliaca* | *A.m.pomonella* | *A.m.ligustica* | 0.061331 | 11.8971 | 2.30E-16 | 0.0423281 | 60868 | 53833.3 |  |
| *A.m.iberiensis* | *A.m.intermissa* | *A.m.caucasica* | 0.0437972 | 11.8894 | 2.30E-16 | 0.0232556 | 83442 | 76439.7 |  |
| *A.m.ligustica* | *A.m.sinisxinyuan* | *A.m.lamarckiiA* | 0.0638158 | 11.8681 | 2.30E-16 | 0.10177 | 84944 | 74752.8 |  |
| *A.m.intermissa* | *A.m.capensis* | *A.m.anatoliaca* | 0.0414587 | 11.8215 | 2.30E-16 | 0.030503 | 97735.8 | 89954.4 |  |
| *A.m.ligustica* | *A.m.iberiensis* | *A.m.jemenitica* | 0.0656989 | 11.8196 | 2.30E-16 | 0.14339 | 106412 | 93291.5 |  |
| *A.m.iberiensis* | *A.m.lamarckiiA* | *A.m.anatoliaca* | 0.0546834 | 11.81 | 2.30E-16 | 0.0420499 | 106327 | 95301.6 |  |
| *XJblackbee* | *A.m.mellifera* | *A.m.unicolor* | 0.0630279 | 11.7536 | 2.30E-16 | 0.0394581 | 97381.9 | 85834.1 |  |
| *A.m.intermissa* | *A.m.capensis* | *A.m.caucasica* | 0.0415091 | 11.6893 | 2.30E-16 | 0.0264228 | 97533.4 | 89759 |  |
| *A.m.monticola* | *A.m.capensis* | *A.m.iberiensis* | 0.0419457 | 11.6594 | 2.30E-16 | 0.0263345 | 100280 | 92205.9 |  |
| *A.m.capensis* | *A.m.adansonii* | *A.m.sinisxinyuan* | 0.0300869 | 11.6348 | 2.30E-16 | 0.0242112 | 94313.2 | 88803.8 |  |
| *XJblackbee* | *A.m.mellifera* | *A.m.lamarckiiA* | 0.0619662 | 11.5724 | 2.30E-16 | 0.117795 | 97709.7 | 86306.9 |  |
| *A.m.iberiensis* | *A.m.lamarckiiA* | *A.m.caucasica* | 0.053644 | 11.54 | 2.30E-16 | 0.0358465 | 105994 | 95201.4 |  |
| *A.m.jemenitica* | *A.m.adansonii* | *A.m.sinisxinyuan* | 0.0297727 | 11.4657 | 2.30E-16 | 0.0243155 | 95700.3 | 90166.5 |  |
| *XJblackbee* | *A.m.sinisxinyuan* | *A.m.monticola* | 0.0520468 | 11.3918 | 2.30E-16 | 0.0856517 | 84230.8 | 75896.6 |  |
| *A.m.unicolor* | *A.m.adansonii* | *XJblackbee* | 0.04435 | 11.2846 | 2.30E-16 | 0.0421816 | 97255.5 | 88995.3 |  |
| *A.m.lamarckii* | *A.m.adansonii* | *A.m.iberiensis* | 0.0356912 | 11.2473 | 2.30E-16 | 0.0230678 | 99299.8 | 92455.9 |  |
| *A.m.monticola* | *A.m.scutellata* | *A.m.mellifera* | 0.0273415 | 11.2163 | 2.30E-16 | 0.0193923 | 96890 | 91732.8 |  |
| *A.m.anatoliaca* | *A.m.lamarckii* | *A.m.mellifera* | 0.0361348 | 11.1153 | 2.30E-16 | 0.0274486 | 102894 | 95717.1 |  |
| *A.m.anatoliaca* | *A.m.syriaca* | *A.m.mellifera* | 0.030558 | 11.0629 | 2.30E-16 | 0.0129934 | 57291.1 | 53893.5 |  |
| *A.m.unicolor* | *A.m.jemenitica* | *A.m.intermissa* | 0.0374413 | 11.061 | 2.30E-16 | 0.0914555 | 108057 | 100258 |  |
| *A.m.scutellata* | *A.m.lamarckii* | *A.m.mellifera* | 0.0344034 | 11.0152 | 2.30E-16 | 0.0246737 | 96737.4 | 90302.6 |  |
| *A.m.lamarckiiA* | *A.m.lamarckii* | *A.m.mellifera* | 0.0304265 | 10.9953 | 2.30E-16 | 0.0217454 | 95730.7 | 90077.2 |  |
| *A.m.adansonii* | *A.m.jemenitica* | *A.m.carnica* | 0.0286192 | 10.9197 | 2.30E-16 | 0.0256376 | 89578.5 | 84593.8 |  |
| *A.m.syriaca* | *A.m.lamarckiiA* | *A.m.iberiensis* | 0.0370486 | 10.8981 | 2.30E-16 | 0.0240672 | 103817 | 96399.6 |  |
| *A.m.pomonella* | *A.m.anatoliaca* | *A.m.jemenitica* | 0.0349965 | 10.818 | 2.30E-16 | 0.0414768 | 48455.5 | 45178.7 |  |
| *A.m.monticola* | *A.m.jemenitica* | *A.m.mellifera* | 0.0383708 | 10.8124 | 2.30E-16 | 0.0278245 | 100122 | 92722.1 |  |
| *A.m.monticola* | *A.m.adansonii* | *A.m.pomonella* | 0.0397893 | 10.702 | 2.30E-16 | 0.0335722 | 98407.7 | 90876.2 |  |
| *A.m.monticola* | *A.m.lamarckii* | *A.m.mellifera* | 0.0312829 | 10.6057 | 2.30E-16 | 0.0223098 | 97793 | 91860.1 |  |
| *A.m.pomonella* | *A.m.caucasica* | *A.m.jemenitica* | 0.0369396 | 10.5894 | 2.30E-16 | 0.0366795 | 40656.6 | 37760 |  |
| *A.m.pomonella* | *XJblackbee* | *A.m.mellifera* | 0.0527645 | 10.5719 | 2.30E-16 | 0.0377703 | 87964.3 | 79146.8 |  |
| *A.m.iberiensis* | *A.m.scutellata* | *A.m.anatoliaca* | 0.0496324 | 10.559 | 2.30E-16 | 0.0382881 | 106164 | 96124 |  |
| *A.m.monticola* | *A.m.scutellata* | *A.m.iberiensis* | 0.0263026 | 10.5396 | 2.30E-16 | 0.0163962 | 98075.2 | 93048.1 |  |
| *A.m.monticola* | *A.m.capensis* | *A.m.intermissa* | 0.037499 | 10.4318 | 2.30E-16 | 0.0945019 | 106570 | 98866.8 |  |
| *A.m.jemenitica* | *A.m.lamarckii* | *A.m.mellifera* | 0.022696 | 10.4311 | 2.30E-16 | 0.0162016 | 94369.5 | 90181 |  |
| *A.m.unicolor* | *A.m.adansonii* | *A.m.ligustica* | 0.0428651 | 10.3918 | 2.30E-16 | 0.0289739 | 95961.6 | 88072.9 |  |
| *A.m.jemenitica* | *A.m.lamarckii* | *A.m.anatoliaca* | 0.0240503 | 10.3395 | 2.30E-16 | 0.0182371 | 94973.4 | 90512.4 |  |
| *A.m.sinisxinyuan* | *A.m.anatoliaca* | *A.m.jemenitica* | 0.0474694 | 10.3265 | 2.30E-16 | 0.107621 | 100750 | 91618.7 |  |
| *A.m.iberiensis* | *A.m.scutellata* | *A.m.caucasica* | 0.0485647 | 10.2983 | 2.30E-16 | 0.0325615 | 105846 | 96041.3 |  |
| *A.m.scutellata* | *A.m.capensis* | *A.m.carnica* | 0.0271981 | 10.2272 | 2.30E-16 | 0.0233834 | 86385.5 | 81810.9 |  |
| *A.m.mellifera* | *A.m.syriaca* | *A.m.unicolor* | 0.0505213 | 10.2245 | 2.30E-16 | 0.0375836 | 109835 | 99270.7 |  |
| *A.m.caucasica* | *A.m.lamarckii* | *A.m.mellifera* | 0.0346379 | 10.0988 | 2.30E-16 | 0.0263871 | 102921 | 96029.5 |  |
| *A.m.scutellata* | *A.m.intermissa* | *A.m.ligustica* | 0.0373624 | 10.098 | 2.30E-16 | 0.0261257 | 95769 | 88870.4 |  |
| *A.m.ligustica* | *A.m.mellifera* | *A.m.jemenitica* | 0.055748 | 10.0847 | 2.30E-16 | 0.113789 | 98781.3 | 88349.1 |  |
| *A.m.capensis* | *A.m.scutellata* | *A.m.monticola* | 0.0185066 | 10.058 | 2.30E-16 | 0.0827466 | 104981 | 101166 |  |
| *A.m.monticola* | *A.m.scutellata* | *A.m.intermissa* | 0.0244913 | 9.97757 | 2.30E-16 | 0.0613334 | 104585 | 99584.4 |  |
| *A.m.carnica* | *A.m.pomonella* | *A.m.intermissa* | 0.0335342 | 9.92805 | 2.30E-16 | 0.0496708 | 79105.4 | 73972.1 |  |
| *A.m.scutellata* | *A.m.intermissa* | *A.m.pomonella* | 0.0313097 | 9.92736 | 2.30E-16 | 0.0277444 | 99413.7 | 93377.5 |  |
| *A.m.scutellata* | *A.m.capensis* | *A.m.ligustica* | 0.028391 | 9.90186 | 2.30E-16 | 0.0183384 | 87699.5 | 82857.2 |  |
| *A.m.scutellata* | *A.m.capensis* | *XJblackbee* | 0.0276574 | 9.89798 | 2.30E-16 | 0.0254596 | 88719.4 | 83944 |  |
| *A.m.jemenitica* | *A.m.lamarckii* | *A.m.caucasica* | 0.0235004 | 9.86478 | 2.30E-16 | 0.0153326 | 94725.5 | 90375.5 |  |
| *A.m.ligustica* | *A.m.sinisxinyuan* | *A.m.capensis* | 0.0546622 | 9.83918 | 2.30E-16 | 0.0880443 | 84133.1 | 75412 |  |
| *A.m.monticola* | *A.m.lamarckii* | *A.m.iberiensis* | 0.0299862 | 9.81049 | 2.30E-16 | 0.0187913 | 98944.6 | 93183.4 |  |
| *XJblackbee* | *A.m.mellifera* | *A.m.capensis* | 0.0541562 | 9.80641 | 2.30E-16 | 0.103903 | 96897.5 | 86941.5 |  |
| *A.m.syriaca* | *A.m.anatoliaca* | *A.m.sinisxinyuan* | 0.0293592 | 9.68609 | 2.30E-16 | 0.0161353 | 59203.7 | 55826.5 |  |
| *A.m.scutellata* | *A.m.capensis* | *A.m.anatoliaca* | 0.0274606 | 9.66146 | 2.30E-16 | 0.019644 | 92715 | 87759.1 |  |
| *A.m.scutellata* | *A.m.capensis* | *A.m.caucasica* | 0.027621 | 9.64112 | 2.30E-16 | 0.0170672 | 92529.3 | 87555.1 |  |
| *A.m.jemenitica* | *A.m.capensis* | *A.m.adansonii* | 0.0224879 | 9.59079 | 2.30E-16 | 0.0976698 | 103069 | 98535.5 |  |
| *A.m.sinisxinyuan* | *A.m.caucasica* | *A.m.jemenitica* | 0.0455087 | 9.58584 | 2.30E-16 | 0.103026 | 100354 | 91617.7 |  |
| *A.m.scutellata* | *A.m.capensis* | *A.m.pomonella* | 0.0255058 | 9.56028 | 2.30E-16 | 0.0210086 | 91890.5 | 87319.6 |  |
| *A.m.monticola* | *A.m.adansonii* | *XJblackbee* | 0.0371943 | 9.46181 | 2.30E-16 | 0.0349418 | 94746.5 | 87951.2 |  |
| *A.m.adansonii* | *A.m.capensis* | *A.m.unicolor* | 0.0318783 | 9.36804 | 2.30E-16 | 0.0263721 | 106170 | 99609.9 |  |
| *A.m.adansonii* | *A.m.capensis* | *A.m.syriaca* | 0.0331257 | 9.36256 | 2.30E-16 | 0.0353526 | 97511.6 | 91258.4 |  |
| *A.m.pomonella* | *A.m.anatoliaca* | *A.m.capensis* | 0.0326293 | 9.35731 | 2.30E-16 | 0.0347142 | 47781.6 | 44761.9 |  |
| *A.m.monticola* | *A.m.scutellata* | *A.m.adansonii* | 0.0230975 | 9.34536 | 2.30E-16 | 0.098159 | 106430 | 101625 |  |
| *A.m.adansonii* | *A.m.capensis* | *A.m.anatoliaca* | 0.0330248 | 9.34009 | 2.30E-16 | 0.0238381 | 94457.3 | 88417.9 |  |
| *XJblackbee* | *A.m.iberiensis* | *A.m.jemenitica* | 0.049744 | 9.33812 | 2.30E-16 | 0.110463 | 102784 | 93043 |  |
| *A.m.adansonii* | *A.m.capensis* | *A.m.caucasica* | 0.0332041 | 9.3098 | 2.30E-16 | 0.0207134 | 94261.5 | 88203 |  |
| *A.m.monticola* | *A.m.jemenitica* | *A.m.iberiensis* | 0.0341266 | 9.29541 | 2.30E-16 | 0.0217327 | 100955 | 94291.7 |  |
| *A.m.syriaca* | *A.m.caucasica* | *A.m.sinisxinyuan* | 0.0317352 | 9.18748 | 2.30E-16 | 0.0173032 | 58862.4 | 55241.3 |  |
| *A.m.lamarckiiA* | *A.m.intermissa* | *A.m.ligustica* | 0.03292 | 9.04194 | 2.30E-16 | 0.0230327 | 95100.5 | 89038.6 |  |
| *A.m.unicolor* | *A.m.adansonii* | *A.m.anatoliaca* | 0.0377818 | 9.02113 | 2.30E-16 | 0.028127 | 100655 | 93326.5 |  |
| *A.m.monticola* | *A.m.lamarckii* | *A.m.intermissa* | 0.0270846 | 8.99495 | 2.30E-16 | 0.0681448 | 105328 | 99772.6 |  |
| *A.m.unicolor* | *A.m.adansonii* | *A.m.caucasica* | 0.037986 | 8.98352 | 2.30E-16 | 0.0245408 | 100474 | 93120.5 |  |
| *A.m.adansonii* | *A.m.capensis* | *A.m.ligustica* | 0.0308633 | 8.93853 | 2.30E-16 | 0.0201835 | 89165.6 | 83826.5 |  |
| *A.m.syriaca* | *A.m.scutellata* | *A.m.iberiensis* | 0.0332152 | 8.93681 | 2.30E-16 | 0.0216878 | 103971 | 97286.4 |  |
| *XJblackbee* | *A.m.sinisxinyuan* | *A.m.scutellata* | 0.042757 | 8.92392 | 2.30E-16 | 0.114962 | 83643.7 | 76784.3 |  |
| *A.m.lamarckiiA* | *A.m.capensis* | *A.m.ligustica* | 0.0235002 | 8.9164 | 2.30E-16 | 0.0152012 | 87117.8 | 83117.2 |  |
| *A.m.pomonella* | *A.m.caucasica* | *A.m.capensis* | 0.0336607 | 8.87841 | 2.30E-16 | 0.0300045 | 40061.9 | 37452.7 |  |
| *A.m.caucasica* | *A.m.syriaca* | *A.m.mellifera* | 0.0281659 | 8.87368 | 2.30E-16 | 0.0118968 | 56713.2 | 53605.9 |  |
| *A.m.pomonella* | *A.m.anatoliaca* | *A.m.scutellata* | 0.0284735 | 8.86352 | 2.30E-16 | 0.0519354 | 47544.3 | 44911.8 |  |
| *A.m.scutellata* | *A.m.capensis* | *A.m.syriaca* | 0.0245544 | 8.8418 | 2.30E-16 | 0.0261017 | 95429.3 | 90855.2 |  |
| *XJblackbee* | *A.m.sinisxinyuan* | *A.m.unicolor* | 0.0433465 | 8.83324 | 2.30E-16 | 0.0236642 | 83313.1 | 76390.5 |  |
| *A.m.adansonii* | *A.m.capensis* | *XJblackbee* | 0.0280121 | 8.81325 | 2.30E-16 | 0.0261462 | 90042.4 | 85135.3 |  |
| *A.m.lamarckiiA* | *A.m.capensis* | *XJblackbee* | 0.0225078 | 8.76309 | 2.30E-16 | 0.0207806 | 88109.8 | 84230.8 |  |
| *A.m.monticola* | *A.m.adansonii* | *A.m.ligustica* | 0.0356797 | 8.70272 | 2.30E-16 | 0.0237718 | 93488.8 | 87047.3 |  |
| *A.m.lamarckiiA* | *A.m.capensis* | *A.m.carnica* | 0.0217139 | 8.64891 | 2.30E-16 | 0.0187224 | 85757.5 | 82112.3 |  |
| *A.m.monticola* | *A.m.syriaca* | *A.m.mellifera* | 0.0374111 | 8.64458 | 2.30E-16 | 0.0293405 | 108179 | 100377 |  |
| *A.m.lamarckii* | *A.m.capensis* | *A.m.monticola* | 0.0181221 | 8.60981 | 2.30E-16 | 0.0738441 | 103212 | 99537.5 |  |
| *A.m.capensis* | *A.m.jemenitica* | *A.m.syriaca* | 0.0183236 | 8.57749 | 2.30E-16 | 0.0202671 | 96111.4 | 92652.5 |  |
| *XJblackbee* | *A.m.sinisxinyuan* | *A.m.lamarckiiA* | 0.0418474 | 8.5182 | 2.30E-16 | 0.0694423 | 83607.3 | 76890.9 |  |
| *A.m.lamarckiiA* | *A.m.intermissa* | *A.m.pomonella* | 0.0261175 | 8.46895 | 2.30E-16 | 0.0231918 | 98656 | 93633.8 |  |
| *A.m.pomonella* | *A.m.anatoliaca* | *A.m.lamarckiiA* | 0.0281342 | 8.45937 | 2.30E-16 | 0.0297341 | 47597.5 | 44992.5 |  |
| *A.m.adansonii* | *A.m.capensis* | *A.m.monticola* | 0.0217188 | 8.44104 | 2.30E-16 | 0.0887324 | 105585 | 101096 |  |
| *A.m.lamarckiiA* | *A.m.capensis* | *A.m.anatoliaca* | 0.0219833 | 8.40453 | 2.30E-16 | 0.0157636 | 92065.6 | 88104.9 |  |
| *A.m.lamarckiiA* | *A.m.capensis* | *A.m.caucasica* | 0.0221003 | 8.39889 | 2.30E-16 | 0.0136822 | 91885 | 87911.4 |  |
| *A.m.jemenitica* | *A.m.capensis* | *A.m.intermissa* | 0.0191109 | 8.3811 | 2.30E-16 | 0.0487668 | 100902 | 97117.9 |  |
| *A.m.pomonella* | *A.m.iberiensis* | *A.m.monticola* | 0.0382202 | 8.30773 | 2.30E-16 | 0.086432 | 103399 | 95785.7 |  |
| *A.m.pomonella* | *A.m.anatoliaca* | *A.m.monticola* | 0.0250006 | 8.28638 | 2.30E-16 | 0.026124 | 47239.7 | 44935.2 |  |
| *A.m.monticola* | *A.m.lamarckii* | *A.m.intermissa* | 0.0338468 | 8.20992 | 2.30E-16 | 0.0862237 | 107350 | 100321 |  |
| *A.m.ligustica* | *A.m.anatoliaca* | *A.m.lamarckii* | 0.037005 | 8.18152 | 2.80E-16 | 0.0328506 | 77716.4 | 72169.8 |  |
| *A.m.anatoliaca* | *A.m.pomonella* | *XJblackbee* | 0.0348494 | 8.15593 | 3.47E-16 | 0.0546565 | 61991.1 | 57815.9 |  |
| *A.m.mellifera* | *A.m.iberiensis* | *A.m.lamarckii* | 0.0233359 | 8.12747 | 4.38E-16 | 0.010357 | 38398.4 | 36647.1 |  |
| *A.m.ligustica* | *A.m.anatoliaca* | *A.m.intermissa* | 0.0359263 | 8.12496 | 4.47E-16 | 0.0482418 | 76584.1 | 71272.1 |  |
| *A.m.mellifera* | *A.m.adansonii* | *A.m.syriaca* | 0.030917 | 8.10557 | 5.25E-16 | 0.03433 | 104812 | 98525.2 |  |
| *A.m.pomonella* | *A.m.caucasica* | *A.m.scutellata* | 0.0284532 | 8.10188 | 5.41E-16 | 0.043483 | 39820.2 | 37616.9 |  |
| *A.m.scutellata* | *A.m.lamarckii* | *A.m.unicolor* | 0.0140449 | 8.09137 | 5.90E-16 | 0.0122575 | 105164 | 102251 |  |
| *A.m.monticola* | *A.m.lamarckii* | *A.m.adansonii* | 0.0245476 | 7.99842 | 1.26E-15 | 0.104826 | 107063 | 101933 |  |
| *A.m.ligustica* | *A.m.sinisxinyuan* | *A.m.jemenitica* | 0.0423368 | 7.99775 | 1.27E-15 | 0.074035 | 83486 | 76704 |  |
| *A.m.capensis* | *A.m.scutellata* | *A.m.unicolor* | 0.0221209 | 7.99258 | 1.32E-15 | 0.0187376 | 104861 | 100322 |  |
| *A.m.lamarckiiA* | *A.m.capensis* | *A.m.pomonella* | 0.0198686 | 7.99083 | 1.34E-15 | 0.016414 | 91225.3 | 87670.9 |  |
| *XJblackbee* | *A.m.anatoliaca* | *A.m.lamarckii* | 0.0300625 | 7.9344 | 2.12E-15 | 0.0254872 | 73195.5 | 68923 |  |
| *A.m.scutellata* | *A.m.jemenitica* | *A.m.sinisxinyuan* | 0.0178684 | 7.84917 | 4.19E-15 | 0.0141341 | 92941 | 89677.9 |  |
| *A.m.unicolor* | *A.m.adansonii* | *A.m.syriaca* | 0.0335284 | 7.84749 | 4.24E-15 | 0.0365458 | 103340 | 96634.8 |  |
| *A.m.intermissa* | *A.m.lamarckii* | *A.m.carnica* | 0.0218528 | 7.80061 | 6.16E-15 | 0.0227154 | 96002.1 | 91896 |  |
| *A.m.pomonella* | *A.m.sinisxinyuan* | *A.m.adansonii* | 0.033244 | 7.7931 | 6.54E-15 | 0.0749989 | 92080.4 | 86155.2 |  |
| *A.m.sinisxinyuan* | *A.m.anatoliaca* | *A.m.lamarckii* | 0.0362584 | 7.78113 | 7.19E-15 | 0.04156 | 101140 | 94062.5 |  |
| *A.m.adansonii* | *A.m.capensis* | *A.m.pomonella* | 0.0210308 | 7.76844 | 7.95E-15 | 0.0176315 | 92785.5 | 88963.2 |  |
| *A.m.pomonella* | *A.m.anatoliaca* | *A.m.unicolor* | 0.0263282 | 7.75962 | 8.52E-15 | 0.00857497 | 47368.3 | 44938.1 |  |
| *A.m.pomonella* | *A.m.caucasica* | *A.m.lamarckiiA* | 0.0281346 | 7.7536 | 8.93E-15 | 0.0248997 | 39845.6 | 37664.9 |  |
| *A.m.sinisxinyuan* | *A.m.iberiensis* | *A.m.lamarckii* | 0.0286214 | 7.72557 | 1.11E-14 | 0.0176493 | 53994.2 | 50989.4 |  |
| *A.m.scutellata* | *A.m.lamarckii* | *A.m.iberiensis* | 0.0255886 | 7.7255 | 1.11E-14 | 0.0160374 | 96920.9 | 92084.5 |  |
| *A.m.caucasica* | *A.m.anatoliaca* | *A.m.ligustica* | 0.0242699 | 7.71235 | 1.24E-14 | 0.0120872 | 42893 | 40860.3 |  |
| *A.m.monticola* | *A.m.adansonii* | *A.m.anatoliaca* | 0.0316889 | 7.70487 | 1.31E-14 | 0.023257 | 98190 | 92158.1 |  |
| *A.m.capensis* | *A.m.lamarckii* | *A.m.mellifera* | 0.0175999 | 7.67782 | 1.62E-14 | 0.0125374 | 93361.7 | 90132.2 |  |
| *A.m.adansonii* | *A.m.capensis* | *A.m.carnica* | 0.019835 | 7.65017 | 2.01E-14 | 0.0174351 | 87145.5 | 83755.7 |  |
| *A.m.monticola* | *A.m.adansonii* | *A.m.caucasica* | 0.0314693 | 7.62381 | 2.46E-14 | 0.0200239 | 97946.9 | 91970.3 |  |
| *A.m.intermissa* | *A.m.lamarckii* | *A.m.syriaca* | 0.0272946 | 7.60967 | 2.75E-14 | 0.0298469 | 100734 | 95381.5 |  |
| *A.m.sinisxinyuan* | *A.m.pomonella* | *A.m.jemenitica* | 0.0330639 | 7.59244 | 3.14E-14 | 0.0692088 | 91823.3 | 85945.6 |  |
| *A.m.intermissa* | *A.m.adansonii* | *A.m.syriaca* | 0.0131155 | 7.55247 | 4.27E-14 | 0.0135865 | 94105.1 | 91668.6 |  |
| *A.m.lamarckii* | *A.m.adansonii* | *A.m.mellifera* | 0.0216421 | 7.54013 | 4.70E-14 | 0.0161258 | 96793 | 92692.1 |  |
| *A.m.lamarckiiA* | *A.m.lamarckii* | *A.m.iberiensis* | 0.0218263 | 7.49829 | 6.47E-14 | 0.0136186 | 95897.5 | 91800.8 |  |
| *A.m.pomonella* | *A.m.caucasica* | *A.m.monticola* | 0.024875 | 7.4974 | 6.51E-14 | 0.021779 | 39568.3 | 37647.5 |  |
| *A.m.syriaca* | *A.m.lamarckii* | *A.m.mellifera* | 0.0195882 | 7.48656 | 7.07E-14 | 0.0146336 | 98301.6 | 94524.5 |  |
| *A.m.lamarckii* | *A.m.capensis* | *A.m.unicolor* | 0.0171753 | 7.47021 | 8.01E-14 | 0.0141518 | 102961 | 99484.3 |  |
| *A.m.jemenitica* | *A.m.lamarckii* | *A.m.iberiensis* | 0.0171549 | 7.35026 | 1.98E-13 | 0.010662 | 94800 | 91602.3 |  |
| *XJblackbee* | *A.m.mellifera* | *A.m.jemenitica* | 0.0381748 | 7.28287 | 3.27E-13 | 0.0796877 | 95744.9 | 88703.6 |  |
| *A.m.capensis* | *A.m.jemenitica* | *A.m.anatoliaca* | 0.014968 | 7.18081 | 6.93E-13 | 0.0110077 | 92305.9 | 89583.3 |  |
| *A.m.ligustica* | *A.m.caucasica* | *A.m.lamarckii* | 0.0334528 | 7.15769 | 8.20E-13 | 0.0298681 | 77864.5 | 72823.6 |  |
| *A.m.ligustica* | *A.m.caucasica* | *A.m.intermissa* | 0.03286 | 7.10552 | 1.20E-12 | 0.0443792 | 76761.7 | 71877.4 |  |
| *A.m.lamarckiiA* | *A.m.jemenitica* | *A.m.sinisxinyuan* | 0.0138176 | 7.08528 | 1.39E-12 | 0.0108888 | 91910.7 | 89405.3 |  |
| *A.m.pomonella* | *A.m.caucasica* | *A.m.unicolor* | 0.0257881 | 7.01961 | 2.22E-12 | 0.00702293 | 39576.6 | 37586.7 |  |
| *A.m.capensis* | *A.m.jemenitica* | *A.m.caucasica* | 0.0151524 | 7.01806 | 2.25E-12 | 0.00959957 | 92119.1 | 89369.1 |  |
| *A.m.sinisxinyuan* | *A.m.caucasica* | *A.m.lamarckii* | 0.0337036 | 6.98745 | 2.80E-12 | 0.038566 | 100682 | 94117 |  |
| *A.m.capensis* | *A.m.jemenitica* | *A.m.pomonella* | 0.013676 | 6.97411 | 3.08E-12 | 0.0115832 | 91429.8 | 88962.8 |  |
| *A.m.scutellata* | *A.m.capensis* | *A.m.sinisxinyuan* | 0.0183368 | 6.93432 | 4.08E-12 | 0.014231 | 91227.3 | 87941.9 |  |
| *A.m.lamarckiiA* | *A.m.capensis* | *A.m.syriaca* | 0.0179005 | 6.88968 | 5.59E-12 | 0.0191398 | 94679.7 | 91349.7 |  |
| *A.m.jemenitica* | *A.m.capensis* | *A.m.unicolor* | 0.0181688 | 6.79252 | 1.10E-11 | 0.0150448 | 103661 | 99961.6 |  |
| *A.m.unicolor* | *A.m.intermissa* | *A.m.caucasica* | 0.0285247 | 6.7776 | 1.22E-11 | 0.0188898 | 102060 | 96398.9 |  |
| *A.m.unicolor* | *A.m.intermissa* | *A.m.anatoliaca* | 0.028201 | 6.77095 | 1.28E-11 | 0.0215182 | 102225 | 96617.7 |  |
| *A.m.sinisxinyuan* | *A.m.anatoliaca* | *A.m.capensis* | 0.0334845 | 6.75541 | 1.42E-11 | 0.0709297 | 98766.5 | 92366.5 |  |
| *A.m.jemenitica* | *A.m.lamarckii* | *A.m.intermissa* | 0.0156895 | 6.62278 | 3.53E-11 | 0.0400497 | 100607 | 97498.5 |  |
| *A.m.pomonella* | *A.m.anatoliaca* | *A.m.lamarckii* | 0.0216246 | 6.61364 | 3.75E-11 | 0.0123974 | 48432.1 | 46381.8 |  |
| *XJblackbee* | *A.m.caucasica* | *A.m.lamarckii* | 0.0266476 | 6.6108 | 3.82E-11 | 0.0224669 | 72520 | 68755.3 |  |
| *XJblackbee* | *A.m.sinisxinyuan* | *A.m.capensis* | 0.0334481 | 6.56638 | 5.16E-11 | 0.0560372 | 82883.7 | 77518.5 |  |
| *A.m.scutellata* | *A.m.capensis* | *A.m.mellifera* | 0.0174918 | 6.41574 | 1.40E-10 | 0.0122886 | 93213.4 | 90008.6 |  |
| *A.m.monticola* | *A.m.adansonii* | *A.m.syriaca* | 0.0268845 | 6.39546 | 1.60E-10 | 0.0289888 | 100843 | 95562.5 |  |
| *A.m.jemenitica* | *A.m.lamarckii* | *A.m.adansonii* | 0.0153436 | 6.36861 | 1.91E-10 | 0.0665757 | 102229 | 99139.1 |  |
| *A.m.iberiensis* | *A.m.mellifera* | *A.m.sinisxinyuan* | 0.0207496 | 6.34011 | 2.30E-10 | 0.0752206 | 60376.6 | 57922 |  |
| *A.m.sinisxinyuan* | *A.m.pomonella* | *A.m.lamarckii* | 0.0278906 | 6.32903 | 2.47E-10 | 0.0296146 | 92988.4 | 87942.1 |  |
| *A.m.carnica* | *A.m.ligustica* | *A.m.jemenitica* | 0.0235791 | 6.32135 | 2.59E-10 | 0.0210743 | 42759.9 | 40789.8 |  |
| *A.m.pomonella* | *A.m.syriaca* | *A.m.intermissa* | 0.0229684 | 6.23976 | 4.38E-10 | 0.0304761 | 66981 | 63973.2 |  |
| *A.m.lamarckiiA* | *A.m.scutellata* | *A.m.monticola* | 0.0107654 | 6.19851 | 5.70E-10 | 0.0501904 | 105013 | 102776 |  |
| *A.m.caucasica* | *A.m.pomonella* | *XJblackbee* | 0.0291049 | 6.1976 | 5.73E-10 | 0.0391338 | 51982.8 | 49042.5 |  |
| *A.m.mellifera* | *A.m.intermissa* | *A.m.syriaca* | 0.0223887 | 6.16699 | 6.96E-10 | 0.0211048 | 88261.9 | 84396.3 |  |
| *A.m.caucasica* | *A.m.sinisxinyuan* | *A.m.adansonii* | 0.0290519 | 6.16037 | 7.26E-10 | 0.0710408 | 98826.9 | 93246.8 |  |
| *A.m.sinisxinyuan* | *A.m.caucasica* | *A.m.capensis* | 0.0312996 | 6.11495 | 9.66E-10 | 0.0661958 | 98350.1 | 92380.4 |  |
| *A.m.pomonella* | *A.m.iberiensis* | *A.m.scutellata* | 0.0294142 | 6.09375 | 1.10E-09 | 0.115958 | 102636 | 96770.8 |  |
| *A.m.scutellata* | *A.m.capensis* | *A.m.jemenitica* | 0.0146456 | 6.07093 | 1.27E-09 | 0.0596581 | 102082 | 99135.1 |  |
| *A.m.capensis* | *A.m.intermissa* | *XJblackbee* | 0.020667 | 6.03302 | 1.61E-09 | 0.0208648 | 94175.7 | 90361.9 |  |
| *A.m.capensis* | *A.m.lamarckii* | *A.m.monticola* | 0.00770507 | 6.03053 | 1.63E-09 | 0.0341879 | 103007 | 101432 |  |
| *A.m.ligustica* | *A.m.pomonella* | *A.m.iberiensis* | 0.037261 | 5.90969 | 3.43E-09 | 0.0210594 | 85897.5 | 79726.2 |  |
| *A.m.anatoliaca* | *A.m.sinisxinyuan* | *A.m.adansonii* | 0.0266698 | 5.85333 | 4.82E-09 | 0.0656931 | 98796.9 | 93664 |  |
| *A.m.caucasica* | *A.m.anatoliaca* | *A.m.carnica* | 0.0159904 | 5.84348 | 5.11E-09 | 0.0120167 | 40925 | 39636.8 |  |
| *A.m.scutellata* | *A.m.capensis* | *A.m.iberiensis* | 0.0163963 | 5.82382 | 5.75E-09 | 0.0100962 | 94374.7 | 91329.8 |  |
| *A.m.scutellata* | *A.m.lamarckii* | *A.m.jemenitica* | 0.00933307 | 5.77728 | 7.59E-09 | 0.0383618 | 102451 | 100556 |  |
| *A.m.intermissa* | *A.m.scutellata* | *A.m.syriaca* | 0.0209185 | 5.76711 | 8.06E-09 | 0.0229242 | 100337 | 96224.8 |  |
| *A.m.intermissa* | *A.m.lamarckii* | *A.m.anatoliaca* | 0.0200862 | 5.76612 | 8.11E-09 | 0.0149673 | 96953.5 | 93135.3 |  |
| *A.m.lamarckiiA* | *A.m.capensis* | *A.m.sinisxinyuan* | 0.0141346 | 5.75716 | 8.55E-09 | 0.0109813 | 90639.4 | 88112.8 |  |
| *A.m.pomonella* | *A.m.iberiensis* | *A.m.unicolor* | 0.0285524 | 5.71429 | 1.10E-08 | 0.0200643 | 102354 | 96671 |  |
| *A.m.anatoliaca* | *A.m.capensis* | *A.m.mellifera* | 0.0195365 | 5.70346 | 1.17E-08 | 0.0151006 | 103021 | 99073.2 |  |
| *A.m.intermissa* | *A.m.lamarckii* | *A.m.caucasica* | 0.0200122 | 5.6984 | 1.21E-08 | 0.012902 | 96741.4 | 92945.4 |  |
| *A.m.pomonella* | *A.m.iberiensis* | *A.m.lamarckiiA* | 0.028033 | 5.66795 | 1.45E-08 | 0.0639098 | 102538 | 96945.7 |  |
| *A.m.intermissa* | *A.m.adansonii* | *A.m.anatoliaca* | 0.00972241 | 5.61216 | 2.00E-08 | 0.00687933 | 91125.6 | 89370.8 |  |
| *A.m.pomonella* | *A.m.caucasica* | *A.m.lamarckii* | 0.019591 | 5.53665 | 3.08E-08 | 0.00940989 | 40483.9 | 38928.1 |  |
| *A.m.sinisxinyuan* | *A.m.syriaca* | *A.m.adansonii* | 0.024097 | 5.53238 | 3.16E-08 | 0.0654543 | 101578 | 96797.9 |  |
| *A.m.intermissa* | *A.m.adansonii* | *A.m.caucasica* | 0.0095761 | 5.53024 | 3.20E-08 | 0.00586197 | 90913.6 | 89189 |  |
| *A.m.unicolor* | *A.m.monticola* | *A.m.adansonii* | 0.020781 | 5.44415 | 5.21E-08 | 0.0819864 | 106950 | 102595 |  |
| *A.m.ligustica* | *A.m.pomonella* | *A.m.lamarckii* | 0.0240083 | 5.42798 | 5.70E-08 | 0.0207973 | 74921.7 | 71408.5 |  |
| *A.m.sinisxinyuan* | *A.m.anatoliaca* | *A.m.lamarckiiA* | 0.0259265 | 5.4241 | 5.82E-08 | 0.0551276 | 98063.2 | 93106.9 |  |
| *A.m.carnica* | *A.m.iberiensis* | *A.m.lamarckii* | 0.0289995 | 5.42338 | 5.85E-08 | 0.0317306 | 96990.6 | 91523.8 |  |
| *XJblackbee* | *A.m.carnica* | *A.m.intermissa* | 0.013537 | 5.41854 | 6.01E-08 | 0.0138538 | 54364.6 | 52912.4 |  |
| *A.m.monticola* | *A.m.intermissa* | *A.m.anatoliaca* | 0.0219604 | 5.38163 | 7.38E-08 | 0.0165499 | 99888.1 | 95595.2 |  |
| *A.m.monticola* | *A.m.intermissa* | *A.m.caucasica* | 0.0218678 | 5.32376 | 1.02E-07 | 0.0142908 | 99667.6 | 95401.8 |  |
| *A.m.sinisxinyuan* | *A.m.anatoliaca* | *A.m.scutellata* | 0.0247168 | 5.32029 | 1.04E-07 | 0.0895864 | 97898.2 | 93175.5 |  |
| *A.m.scutellata* | *A.m.lamarckii* | *A.m.lamarckii* | 0.00943662 | 5.31759 | 1.05E-07 | 0.013734 | 102812 | 100890 |  |
| *A.m.lamarckiiA* | *A.m.capensis* | *A.m.mellifera* | 0.0132836 | 5.31663 | 1.06E-07 | 0.00933811 | 92600.1 | 90172.2 |  |
| *A.m.capensis* | *A.m.jemenitica* | *XJblackbee* | 0.0112045 | 5.28243 | 1.27E-07 | 0.0106323 | 87700.2 | 85756.7 |  |
| *A.m.anatoliaca* | *A.m.caucasica* | *XJblackbee* | 0.0138795 | 5.26709 | 1.39E-07 | 0.0160869 | 44873.6 | 43645 |  |
| *A.m.scutellata* | *A.m.capensis* | *A.m.lamarckii* | 0.0141309 | 5.20065 | 1.99E-07 | 0.0203741 | 102326 | 99474 |  |
| *A.m.caucasica* | *A.m.capensis* | *A.m.mellifera* | 0.0181017 | 5.04083 | 4.64E-07 | 0.014025 | 103003 | 99340.5 |  |
| *A.m.carnica* | *A.m.ligustica* | *A.m.capensis* | 0.0198311 | 5.01137 | 5.40E-07 | 0.0163742 | 42341.8 | 40695.1 |  |
| *A.m.sinisxinyuan* | *A.m.anatoliaca* | *A.m.unicolor* | 0.0242102 | 5.00928 | 5.46E-07 | 0.0161648 | 97595.6 | 92981.7 |  |
| *A.m.unicolor* | *A.m.monticola* | *A.m.intermissa* | 0.0192163 | 4.98204 | 6.29E-07 | 0.0463448 | 104812 | 100860 |  |
| *A.m.jemenitica* | *A.m.lamarckii* | *A.m.adansonii* | 0.0125504 | 4.94602 | 7.57E-07 | 0.0551074 | 103163 | 100606 |  |
| *A.m.unicolor* | *A.m.intermissa* | *A.m.syriaca* | 0.0209931 | 4.92165 | 8.58E-07 | 0.023423 | 104516 | 100218 |  |
| *A.m.capensis* | *A.m.jemenitica* | *A.m.ligustica* | 0.011166 | 4.90826 | 9.19E-07 | 0.00737944 | 86606.8 | 84694.1 |  |
| *A.m.scutellata* | *A.m.jemenitica* | *A.m.mellifera* | 0.0120367 | 4.89452 | 9.85E-07 | 0.0086057 | 94355.6 | 92111.2 |  |
| *A.m.ligustica* | *XJblackbee* | *A.m.lamarckii* | 0.0153729 | 4.89284 | 9.94E-07 | 0.00756843 | 42233.7 | 40954.8 |  |
| *A.m.lamarckiiA* | *A.m.capensis* | *A.m.iberiensis* | 0.0124569 | 4.88717 | 1.02E-06 | 0.00766926 | 93762.2 | 91455 |  |
| *A.m.scutellata* | *A.m.capensis* | *A.m.intermissa* | 0.0135914 | 4.87053 | 1.11E-06 | 0.0352534 | 100606 | 97907.6 |  |
| *A.m.monticola* | *A.m.jemenitica* | *A.m.intermissa* | 0.0188058 | 4.85508 | 1.20E-06 | 0.0480326 | 106066 | 102151 |  |
| *A.m.pomonella* | *A.m.mellifera* | *A.m.monticola* | 0.0217102 | 4.85028 | 1.23E-06 | 0.0471497 | 97901.5 | 93740.9 |  |
| *A.m.sinisxinyuan* | *A.m.caucasica* | *A.m.lamarckiiA* | 0.0236782 | 4.79156 | 1.65E-06 | 0.0502661 | 97642.8 | 93125.8 |  |
| *A.m.sinisxinyuan* | *A.m.caucasica* | *A.m.scutellata* | 0.0224325 | 4.66558 | 3.08E-06 | 0.0811915 | 97485.5 | 93207.8 |  |
| *A.m.anatoliaca* | *A.m.caucasica* | *A.m.syriaca* | 0.0148736 | 4.6487 | 3.34E-06 | 0.0766326 | 54556.9 | 52957.8 |  |
| *A.m.capensis* | *A.m.jemenitica* | *A.m.carnica* | 0.00948603 | 4.63097 | 3.64E-06 | 0.00837841 | 85180.3 | 83579.4 |  |
| *A.m.unicolor* | *A.m.monticola* | *A.m.iberiensis* | 0.01794 | 4.59721 | 4.28E-06 | 0.0111599 | 98082.3 | 94625.1 |  |
| *A.m.adansonii* | *A.m.lamarckii* | *A.m.unicolor* | 0.0149954 | 4.58294 | 4.58E-06 | 0.012398 | 104368 | 101284 |  |
| *A.m.carnica* | *A.m.ligustica* | *A.m.scutellata* | 0.0167757 | 4.54218 | 5.57E-06 | 0.0216358 | 42229.5 | 40836 |  |
| *A.m.unicolor* | *A.m.monticola* | *A.m.mellifera* | 0.017224 | 4.53226 | 5.84E-06 | 0.0121792 | 96742.8 | 93466.7 |  |
| *A.m.anatoliaca* | *A.m.jemenitica* | *A.m.mellifera* | 0.0149765 | 4.49186 | 7.06E-06 | 0.0114777 | 101697 | 98695.4 |  |
| *XJblackbee* | *A.m.pomonella* | *A.m.lamarckii* | 0.015944 | 4.47076 | 7.79E-06 | 0.013336 | 71261.8 | 69025.1 |  |
| *A.m.intermissa* | *A.m.mellifera* | *A.m.caucasica* | 0.0159098 | 4.39728 | 1.10E-05 | 0.0091365 | 85814.4 | 83126.6 |  |
| *XJblackbee* | *A.m.sinisxinyuan* | *A.m.jemenitica* | 0.0211064 | 4.3772 | 1.20E-05 | 0.03852 | 82264.7 | 78863.9 |  |
| *A.m.sinisxinyuan* | *A.m.caucasica* | *A.m.unicolor* | 0.0218074 | 4.36795 | 1.25E-05 | 0.0145317 | 97138.6 | 92992.4 |  |
| *A.m.unicolor* | *A.m.monticola* | *A.m.sinisxinyuan* | 0.0159554 | 4.33 | 1.49E-05 | 0.012426 | 94553.2 | 91583.3 |  |
| *A.m.carnica* | *A.m.ligustica* | *A.m.unicolor* | 0.0166926 | 4.31126 | 1.62E-05 | 0.00466379 | 42107.1 | 40724.4 |  |
| *A.m.lamarckiiA* | *A.m.capensis* | *A.m.intermissa* | 0.0108337 | 4.29636 | 1.74E-05 | 0.028235 | 100066 | 97920.9 |  |
| *A.m.pomonella* | *A.m.iberiensis* | *A.m.capensis* | 0.0217767 | 4.24258 | 2.21E-05 | 0.0499508 | 101802 | 97462.6 |  |
| *A.m.intermissa* | *A.m.scutellata* | *A.m.anatoliaca* | 0.0148268 | 4.21809 | 2.46E-05 | 0.0110794 | 96730.2 | 93903.7 |  |
| *A.m.adansonii* | *A.m.lamarckii* | *A.m.syriaca* | 0.0152655 | 4.21091 | 2.54E-05 | 0.0165101 | 97108.7 | 94188.5 |  |
| *A.m.intermissa* | *A.m.scutellata* | *A.m.caucasica* | 0.0147164 | 4.15797 | 3.21E-05 | 0.00951481 | 96519 | 93719.4 |  |
| *A.m.sinisxinyuan* | *A.m.pomonella* | *A.m.capensis* | 0.0192352 | 4.11273 | 3.91E-05 | 0.0376329 | 90050.5 | 86651.6 |  |
| *A.m.carnica* | *A.m.ligustica* | *A.m.lamarckiiA* | 0.0155507 | 4.10613 | 4.02E-05 | 1. 0127599 | 42201.4 | 40908.9 |  |
| *A.m.carnica* | *A.m.mellifera* | *A.m.lamarckii* | 0.0214841 | 4.08577 | 4.39E-05 | 0.0219119 | 89847.8 | 86068.4 |  |
| *A.m.sinisxinyuan* | *A.m.mellifera* | *A.m.lamarckii* | 0.0124283 | 4.07482 | 4.60E-05 | 0.00750985 | 52117.5 | 50837.9 |  |
| *A.m.mellifera* | *A.m.pomonella* | *A.m.lamarckii* | 0.0191941 | 4.06457 | 4.81E-05 | 0.0221933 | 99700.6 | 95945.4 |  |
| *A.m.jemenitica* | *A.m.lamarckii* | *A.m.monticola* | 0.00795741 | 4.06188 | 4.87E-05 | 0.0314394 | 102307 | 100692 |  |
| *A.m.scutellata* | *A.m.capensis* | *A.m.adansonii* | 0.0113361 | 4.03437 | 5.47E-05 | 0.0519368 | 102308 | 100015 |  |
| *A.m.carnica* | *A.m.ligustica* | *A.m.monticola* | 0.0139052 | 3.94336 | 8.03E-05 | 0.0113397 | 42022.2 | 40869.6 |  |
| *A.m.lamarckiiA* | *A.m.capensis* | *A.m.adansonii* | 0.0097839 | 3.89086 | 9.99E-05 | 0.0450469 | 101866 | 99892 |  |
| *A.m.jemenitica* | *A.m.lamarckii* | *A.m.scutellata* | 0.00753948 | 3.86521 | 0.000110996 | 0.109499 | 102503 | 100969 |  |
| *A.m.capensis* | *A.m.lamarckii* | *A.m.iberiensis* | 0.00967484 | 3.8614 | 0.000112737 | 0.0060099 | 93616.9 | 91822.8 |  |
| *A.m.caucasica* | *A.m.jemenitica* | *A.m.mellifera* | 0.0134987 | 3.83966 | 0.000123206 | 0.0103689 | 101664 | 98956.3 |  |
| *A.m.jemenitica* | *A.m.scutellata* | *A.m.adansonii* | 0.0109201 | 3.83012 | 0.000128083 | 0.0483081 | 103824 | 101581 |  |
| *A.m.adansonii* | *A.m.jemenitica* | *A.m.unicolor* | 0.0137422 | 3.79992 | 0.00014474 | 0.0114899 | 105420 | 102562 |  |
| *A.m.lamarckiiA* | *A.m.jemenitica* | *A.m.mellifera* | 0.00791926 | 3.78538 | 0.000153475 | 0.00563906 | 93302.3 | 91836.1 |  |
| *A.m.jemenitica* | *A.m.capensis* | *A.m.iberiensis* | 0.00758999 | 3.69112 | 0.000223269 | 0.00468943 | 93358.2 | 91951.7 |  |
| *A.m.scutellata* | *A.m.lamarckii* | *A.m.syriaca* | 0.00663401 | 3.64281 | 0.000269683 | 0.0071014 | 94417 | 93172.5 |  |
| *A.m.scutellata* | *A.m.syriaca* | *A.m.mellifera* | 0.0131361 | 3.63601 | 0.000276891 | 0.0101633 | 102213 | 99562.2 |  |
| *A.m.sinisxinyuan* | *A.m.anatoliaca* | *A.m.monticola* | 0.0160527 | 3.5971 | 0.00032178 | 0.034474 | 96913.6 | 93851.3 |  |
| *A.m.sinisxinyuan* | *XJblackbee* | *A.m.lamarckii* | 0.0169705 | 3.56652 | 0.000361755 | 0.0165568 | 84551.6 | 81729.7 |  |
| *A.m.carnica* | *A.m.sinisxinyuan* | *A.m.lamarckii* | 0.0160297 | 3.42003 | 0.000626144 | 0.0144987 | 79205.9 | 76706.6 |  |
| *A.m.monticola* | *A.m.intermissa* | *A.m.syriaca* | 0.0141591 | 3.37419 | 0.000740339 | 0.0156579 | 102156 | 99303.7 |  |
| *A.m.scutellata* | *A.m.jemenitica* | *A.m.iberiensis* | 0.00866177 | 3.34269 | 0.000829707 | 0.00542393 | 95244.3 | 93608.5 |  |
| *A.m.intermissa* | *A.m.mellifera* | *A.m.anatoliaca* | 0.0117491 | 3.33465 | 0.00085408 | 0.00778569 | 85505.1 | 83519.2 |  |
| *A.m.scutellata* | *A.m.lamarckii* | *A.m.pomonella* | 0.00562857 | 3.31068 | 0.000930706 | 0.00466649 | 90695.7 | 89680.4 |  |
| *A.m.scutellata* | *A.m.lamarckii* | *A.m.carnica* | 0.00544924 | 3.30438 | 0.00095188 | 0.00471932 | 85173.4 | 84250.1 |  |
| *A.m.jemenitica* | *A.m.lamarckii* | *A.m.intermissa* | 0.00816257 | 3.22447 | 0.00126204 | 0.021058 | 100914 | 99279.5 |  |
| *A.m.adansonii* | *A.m.lAmarckii* | *A.m.caucasica* | 0.0112517 | 3.18074 | 0.00146897 | 0.00711031 | 93455.7 | 91376 |  |
| *A.m.adansonii* | *A.m.lAmarckii* | *A.m.anatoliaca* | 0.0111893 | 3.17916 | 0.00147701 | 0.00818042 | 93644 | 91571.6 |  |
| *A.m.pomonella* | *XJblackbee* | *A.m.iberiensis* | 0.0164537 | 3.11334 | 0.00184984 | 0.00932524 | 82627.4 | 79952.3 |  |
| *A.m.capensis* | *A.m.intermissa* | *A.m.ligustica* | 0.0113397 | 3.04593 | 0.00231962 | 0.00794647 | 91848.6 | 89788.9 |  |
| *A.m.unicolor* | *A.m.monticola* | *A.m.capensis* | 0.010809 | 3.03937 | 0.00237075 | 0.0391097 | 106616 | 104335 |  |
| *A.m.sinisxinyuan* | *A.m.caucasica* | *A.m.monticola* | 0.0139503 | 3.01912 | 0.00253509 | 0.0299134 | 96520.9 | 93864.9 |  |
| *A.m.jemenitica* | *A.m.intermissa* | *XJblackbee* | 0.00996485 | 3.00388 | 0.0026656 | 0.0103358 | 94716.6 | 92847.6 |  |

| **Table S8. qpAdm admixture modeling results for Xinjiang black honeybee (XJ) with different source populations.** | | | | |
| --- | --- | --- | --- | --- |
| **target** | **left** | **weight** | **se** | **z** |
| XJ | *A.m.carnica* | 0.9 | 0.0029 | 313.62 |
| XJ | *A.m.sinisxinyuan* | 0.1 | 0.0029 | 33.02 |

|  | **Table S9:Introgressed genes (NCBI accession numbers) were identified using at least one statistical test (Fd, Dxy, or ELAI) and used for GO enrichment analysis.** | | | |
| --- | --- | --- | --- | --- |
| No. | all introgressed gene | Fd TOP gene list | Dxy TOP gene list | ElAI TOP gene list |
| 1 | 5-ht7 | 5-ht7 | 5-ht7 | Glurb |
| 2 | AGLU2 | Crz | AGLU2 | LOC100576262 |
| 3 | A.m.ih | Dcr-1 | A.m.ih | LOC100576471 |
| 4 | Crz | Fibroin1 | Csd | LOC100576484 |
| 5 | Csd | Fibroin2 | Fem | LOC100576855 |
| 6 | Dcr-1 | LOC100576348 | Grp | LOC100577150 |
| 7 | Fem | LOC100576436 | Hbg3 | LOC100577283 |
| 8 | Fibroin1 | LOC100576439 | Hex70a | LOC100577522 |
| 9 | Fibroin2 | LOC100576527 | Ing3 | LOC102654281 |
| 10 | Glurb | LOC100576840 | LOC100576116 | LOC102654331 |
| 11 | Grp | LOC100576871 | LOC100576151 | LOC102655879 |
| 12 | Hbg3 | LOC100576896 | LOC100576462 | LOC102656585 |
| 13 | Hex70a | LOC100576975 | LOC100576497 | LOC107965756 |
| 14 | Ing3 | LOC100577280 | LOC100576531 | LOC107966034 |
| 15 | LOC100576116 | LOC100577285 | LOC100576572 | LOC112939925 |
| 16 | LOC100576151 | LOC100577919 | LOC100576638 | LOC113218555 |
| 17 | LOC100576262 | LOC100577958 | LOC100576722 | LOC113219092 |
| 18 | LOC100576348 | LOC100577997 | LOC100576851 | LOC408394 |
| 19 | LOC100576436 | LOC100578212 | LOC100576885 | LOC408444 |
| 20 | LOC100576439 | LOC100578223 | LOC100576896 | LOC408959 |
| 21 | LOC100576462 | LOC100578529 | LOC100576918 | LOC408960 |
| 22 | LOC100576471 | LOC100578653 | LOC100576919 | LOC408976 |
| 23 | LOC100576484 | LOC100579026 | LOC100576951 | LOC409095 |
| 24 | LOC100576497 | LOC102653641 | LOC100576975 | LOC409653 |
| 25 | LOC100576527 | LOC102654165 | LOC100576990 | LOC409665 |
| 26 | LOC100576531 | LOC102654184 | LOC100577308 | LOC409666 |
| 27 | LOC100576572 | LOC102654594 | LOC100577367 | LOC409667 |
| 28 | LOC100576638 | LOC102654949 | LOC100577423 | LOC409692 |
| 29 | LOC100576722 | LOC102655130 | LOC100577580 | LOC409740 |
| 30 | LOC100576840 | LOC102655470 | LOC100578314 | LOC409783 |
| 31 | LOC100576851 | LOC102655636 | LOC100578450 | LOC410044 |
| 32 | LOC100576855 | LOC102656074 | LOC100578464 | LOC410094 |
| 33 | LOC100576871 | LOC102656283 | LOC100578468 | LOC410317 |
| 34 | LOC100576885 | LOC102656492 | LOC100578680 | LOC410318 |
| 35 | LOC100576896 | LOC102656685 | LOC100578694 | LOC410393 |
| 36 | LOC100576918 | LOC102656893 | LOC100578721 | LOC410470 |
| 37 | LOC100576919 | LOC107965571 | LOC100578782 | LOC410484 |
| 38 | LOC100576951 | LOC107965756 | LOC100578892 | LOC410780 |
| 39 | LOC100576975 | LOC107965825 | LOC100578943 | LOC410926 |
| 40 | LOC100576990 | LOC107966050 | LOC102653798 | LOC411054 |
| 41 | LOC100577150 | LOC113218539 | LOC102654281 | LOC411245 |
| 42 | LOC100577280 | LOC113218575 | LOC102654331 | LOC411336 |
| 43 | LOC100577283 | LOC113218736 | LOC102654781 | LOC411843 |
| 44 | LOC100577285 | LOC113218737 | LOC102654783 | LOC413034 |
| 45 | LOC100577308 | LOC113218745 | LOC102655111 | LOC413382 |
| 46 | LOC100577367 | LOC113218983 | LOC102655126 | LOC413582 |
| 47 | LOC100577423 | LOC113218987 | LOC102656429 | LOC413616 |
| 48 | LOC100577522 | LOC113219003 | LOC102656541 | LOC413829 |
| 49 | LOC100577580 | LOC113219005 | LOC102656579 | LOC413908 |
| 50 | LOC100577919 | LOC113219010 | LOC102656779 | LOC413994 |
| 51 | LOC100577958 | LOC113219412 | LOC102656792 | LOC551170 |
| 52 | LOC100577997 | LOC113219418 | LOC107964839 | LOC551356 |
| 53 | LOC100578212 | LOC406118 | LOC107965022 | LOC551746 |
| 54 | LOC100578223 | LOC406139 | LOC107965449 | LOC551957 |
| 55 | LOC100578314 | LOC406152 | LOC107965775 | LOC552173 |
| 56 | LOC100578450 | LOC408411 | LOC107965795 | LOC724269 |
| 57 | LOC100578464 | LOC408721 | LOC113219102 | LOC724603 |
| 58 | LOC100578468 | LOC408725 | LOC113219105 | LOC724773 |
| 59 | LOC100578529 | LOC408871 | LOC113219416 | LOC725021 |
| 60 | LOC100578653 | LOC408902 | LOC408291 | LOC725890 |
| 61 | LOC100578680 | LOC408903 | LOC408656 | LOC725894 |
| 62 | LOC100578694 | LOC408904 | LOC408657 | LOC726068 |
| 63 | LOC100578721 | LOC408905 | LOC408733 | LOC726167 |
| 64 | LOC100578782 | LOC408969 | LOC408742 | LOC726415 |
| 65 | LOC100578892 | LOC409036 | LOC409111 | LOC726496 |
| 66 | LOC100578943 | LOC409085 | LOC409200 | LOC726690 |
| 67 | LOC100579026 | LOC409102 | LOC409204 | LOC726948 |
| 68 | LOC102653641 | LOC409103 | LOC409285 | LOC727000 |
| 69 | LOC102653798 | LOC409111 | LOC409288 | LOC727598 |
| 70 | LOC102654165 | LOC409217 | LOC409353 | NLG-3 |
| 71 | LOC102654184 | LOC409267 | LOC409438 | Obp3 |
| 72 | LOC102654281 | LOC409279 | LOC409499 | Obp4 |
| 73 | LOC102654331 | LOC409320 | LOC409505 |  |
| 74 | LOC102654594 | LOC409360 | LOC409540 |  |
| 75 | LOC102654781 | LOC409365 | LOC409550 |  |
| 76 | LOC102654783 | LOC409420 | LOC409554 |  |
| 77 | LOC102654949 | LOC409510 | LOC409579 |  |
| 78 | LOC102655111 | LOC409520 | LOC409617 |  |
| 79 | LOC102655126 | LOC409564 | LOC409637 |  |
| 80 | LOC102655130 | LOC409606 | LOC409655 |  |
| 81 | LOC102655470 | LOC409607 | LOC409741 |  |
| 82 | LOC102655636 | LOC409624 | LOC409787 |  |
| 83 | LOC102655879 | LOC409674 | LOC409809 |  |
| 84 | LOC102656074 | LOC409686 | LOC409810 |  |
| 85 | LOC102656283 | LOC409741 | LOC409912 |  |
| 86 | LOC102656429 | LOC409755 | LOC410044 |  |
| 87 | LOC102656492 | LOC409756 | LOC410067 |  |
| 88 | LOC102656541 | LOC409757 | LOC410207 |  |
| 89 | LOC102656579 | LOC409780 | LOC410717 |  |
| 90 | LOC102656585 | LOC409912 | LOC410804 |  |
| 91 | LOC102656685 | LOC409915 | LOC410805 |  |
| 92 | LOC102656779 | LOC409981 | LOC410806 |  |
| 93 | LOC102656792 | LOC410038 | LOC410936 |  |
| 94 | LOC102656893 | LOC410057 | LOC411539 |  |
| 95 | LOC107964839 | LOC410060 | LOC411540 |  |
| 96 | LOC107965022 | LOC410061 | LOC411541 |  |
| 97 | LOC107965449 | LOC410070 | LOC411630 |  |
| 98 | LOC107965571 | LOC410071 | LOC411746 |  |
| 99 | LOC107965756 | LOC410620 | LOC411797 |  |
| 100 | LOC107965775 | LOC410621 | LOC411805 |  |
| 101 | LOC107965795 | LOC410694 | LOC411811 |  |
| 102 | LOC107965825 | LOC410728 | LOC411904 |  |
| 103 | LOC107966034 | LOC411059 | LOC411989 |  |
| 104 | LOC107966050 | LOC411060 | LOC412069 |  |
| 105 | LOC112939925 | LOC411116 | LOC412109 |  |
| 106 | LOC113218539 | LOC411154 | LOC412176 |  |
| 107 | LOC113218555 | LOC411155 | LOC412192 |  |
| 108 | LOC113218575 | LOC411191 | LOC412234 |  |
| 109 | LOC113218736 | LOC411192 | LOC412235 |  |
| 110 | LOC113218737 | LOC411193 | LOC412398 |  |
| 111 | LOC113218745 | LOC411276 | LOC412399 |  |
| 112 | LOC113218983 | LOC411447 | LOC412423 |  |
| 113 | LOC113218987 | LOC411631 | LOC412503 |  |
| 114 | LOC113219003 | LOC411748 | LOC412504 |  |
| 115 | LOC113219005 | LOC411806 | LOC412594 |  |
| 116 | LOC113219010 | LOC411886 | LOC412647 |  |
| 117 | LOC113219092 | LOC411960 | LOC412830 |  |
| 118 | LOC113219102 | LOC412025 | LOC412890 |  |
| 119 | LOC113219105 | LOC412164 | LOC412929 |  |
| 120 | LOC113219412 | LOC412192 | LOC413189 |  |
| 121 | LOC113219416 | LOC412289 | LOC413190 |  |
| 122 | LOC113219418 | LOC412291 | LOC413382 |  |
| 123 | LOC406118 | LOC412343 | LOC413490 |  |
| 124 | LOC406139 | LOC412355 | LOC413578 |  |
| 125 | LOC406152 | LOC412396 | LOC413616 |  |
| 126 | LOC408291 | LOC412452 | LOC413947 |  |
| 127 | LOC408394 | LOC412510 | LOC550698 |  |
| 128 | LOC408411 | LOC412522 | LOC550735 |  |
| 129 | LOC408444 | LOC412531 | LOC550834 |  |
| 130 | LOC408656 | LOC412546 | LOC550915 |  |
| 131 | LOC408657 | LOC412661 | LOC551020 |  |
| 132 | LOC408721 | LOC412674 | LOC551053 |  |
| 133 | LOC408725 | LOC412714 | LOC551079 |  |
| 134 | LOC408733 | LOC412784 | LOC551094 |  |
| 135 | LOC408742 | LOC412818 | LOC551149 |  |
| 136 | LOC408871 | LOC412829 | LOC551201 |  |
| 137 | LOC408902 | LOC412885 | LOC551240 |  |
| 138 | LOC408903 | LOC413024 | LOC551308 |  |
| 139 | LOC408904 | LOC413080 | LOC551376 |  |
| 140 | LOC408905 | LOC413081 | LOC551408 |  |
| 141 | LOC408959 | LOC413126 | LOC551497 |  |
| 142 | LOC408960 | LOC413256 | LOC551554 |  |
| 143 | LOC408969 | LOC413450 | LOC551566 |  |
| 144 | LOC408976 | LOC413467 | LOC551571 |  |
| 145 | LOC409036 | LOC413468 | LOC551588 |  |
| 146 | LOC409085 | LOC413524 | LOC551696 |  |
| 147 | LOC409095 | LOC413590 | LOC551754 |  |
| 148 | LOC409102 | LOC413591 | LOC551888 |  |
| 149 | LOC409103 | LOC413758 | LOC551905 |  |
| 150 | LOC409111 | LOC413759 | LOC552002 |  |
| 151 | LOC409200 | LOC413907 | LOC552036 |  |
| 152 | LOC409204 | LOC413926 | LOC552063 |  |
| 153 | LOC409217 | LOC414021 | LOC552065 |  |
| 154 | LOC409267 | LOC414022 | LOC552089 |  |
| 155 | LOC409279 | LOC550689 | LOC552091 |  |
| 156 | LOC409285 | LOC550692 | LOC552115 |  |
| 157 | LOC409288 | LOC550698 | LOC552273 |  |
| 158 | LOC409320 | LOC550745 | LOC552284 |  |
| 159 | LOC409353 | LOC550780 | LOC552291 |  |
| 160 | LOC409360 | LOC550969 | LOC552301 |  |
| 161 | LOC409365 | LOC551097 | LOC552315 |  |
| 162 | LOC409420 | LOC551188 | LOC552372 |  |
| 163 | LOC409438 | LOC551222 | LOC552467 |  |
| 164 | LOC409499 | LOC551308 | LOC552490 |  |
| 165 | LOC409505 | LOC551371 | LOC552616 |  |
| 166 | LOC409510 | LOC551381 | LOC552642 |  |
| 167 | LOC409520 | LOC551398 | LOC552644 |  |
| 168 | LOC409540 | LOC551445 | LOC552654 |  |
| 169 | LOC409550 | LOC551469 | LOC552660 |  |
| 170 | LOC409554 | LOC551523 | LOC552678 |  |
| 171 | LOC409564 | LOC551524 | LOC552686 |  |
| 172 | LOC409579 | LOC551554 | LOC724333 |  |
| 173 | LOC409606 | LOC551557 | LOC724380 |  |
| 174 | LOC409607 | LOC551576 | LOC724389 |  |
| 175 | LOC409617 | LOC551620 | LOC724417 |  |
| 176 | LOC409624 | LOC551644 | LOC724467 |  |
| 177 | LOC409637 | LOC551673 | LOC724551 |  |
| 178 | LOC409653 | LOC551677 | LOC724596 |  |
| 179 | LOC409655 | LOC551682 | LOC724669 |  |
| 180 | LOC409665 | LOC551696 | LOC724673 |  |
| 181 | LOC409666 | LOC551724 | LOC724681 |  |
| 182 | LOC409667 | LOC551754 | LOC724757 |  |
| 183 | LOC409674 | LOC551769 | LOC724826 |  |
| 184 | LOC409686 | LOC551784 | LOC724869 |  |
| 185 | LOC409692 | LOC551790 | LOC724870 |  |
| 186 | LOC409740 | LOC551818 | LOC724886 |  |
| 187 | LOC409741 | LOC551824 | LOC724893 |  |
| 188 | LOC409755 | LOC551907 | LOC724903 |  |
| 189 | LOC409756 | LOC551936 | LOC725039 |  |
| 190 | LOC409757 | LOC551939 | LOC725097 |  |
| 191 | LOC409780 | LOC552025 | LOC725268 |  |
| 192 | LOC409783 | LOC552106 | LOC725316 |  |
| 193 | LOC409787 | LOC552125 | LOC725524 |  |
| 194 | LOC409809 | LOC552150 | LOC725599 |  |
| 195 | LOC409810 | LOC552172 | LOC725673 |  |
| 196 | LOC409912 | LOC552198 | LOC725770 |  |
| 197 | LOC409915 | LOC552249 | LOC725792 |  |
| 198 | LOC409981 | LOC552273 | LOC725850 |  |
| 199 | LOC410038 | LOC552289 | LOC725905 |  |
| 200 | LOC410044 | LOC552291 | LOC726007 |  |
| 201 | LOC410057 | LOC552293 | LOC726056 |  |
| 202 | LOC410060 | LOC552345 | LOC726108 |  |
| 203 | LOC410061 | LOC552421 | LOC726220 |  |
| 204 | LOC410067 | LOC552435 | LOC726272 |  |
| 205 | LOC410070 | LOC552442 | LOC726277 |  |
| 206 | LOC410071 | LOC552488 | LOC726320 |  |
| 207 | LOC410094 | LOC552502 | LOC726326 |  |
| 208 | LOC410207 | LOC552549 | LOC726368 |  |
| 209 | LOC410317 | LOC552662 | LOC726389 |  |
| 210 | LOC410318 | LOC552677 | LOC726405 |  |
| 211 | LOC410393 | LOC552709 | LOC726431 |  |
| 212 | LOC410470 | LOC724118 | LOC726444 |  |
| 213 | LOC410484 | LOC724131 | LOC726488 |  |
| 214 | LOC410620 | LOC724167 | LOC726740 |  |
| 215 | LOC410621 | LOC724193 | LOC726778 |  |
| 216 | LOC410694 | LOC724215 | LOC726811 |  |
| 217 | LOC410717 | LOC724269 | LOC726822 |  |
| 218 | LOC410728 | LOC724296 | LOC727049 |  |
| 219 | LOC410780 | LOC724329 | LOC727106 |  |
| 220 | LOC410804 | LOC724421 | LOC727119 |  |
| 221 | LOC410805 | LOC724450 | LOC727122 |  |
| 222 | LOC410806 | LOC724466 | LOC727153 |  |
| 223 | LOC410926 | LOC724481 | LOC727157 |  |
| 224 | LOC410936 | LOC724513 | LOC727243 |  |
| 225 | LOC411054 | LOC724536 | LOC727290 |  |
| 226 | LOC411059 | LOC724538 | LOC727299 |  |
| 227 | LOC411060 | LOC724560 | LOC727378 |  |
| 228 | LOC411116 | LOC724585 | LOC727436 |  |
| 229 | LOC411154 | LOC724634 | LOC727522 |  |
| 230 | LOC411155 | LOC724673 | LOC727634 |  |
| 231 | LOC411191 | LOC724676 |  |  |
| 232 | LOC411192 | LOC724706 |  |  |
| 233 | LOC411193 | LOC724760 |  |  |
| 234 | LOC411245 | LOC724794 |  |  |
| 235 | LOC411276 | LOC724843 |  |  |
| 236 | LOC411336 | LOC724875 |  |  |
| 237 | LOC411447 | LOC724912 |  |  |
| 238 | LOC411539 | LOC724999 |  |  |
| 239 | LOC411540 | LOC725024 |  |  |
| 240 | LOC411541 | LOC725031 |  |  |
| 241 | LOC411630 | LOC725040 |  |  |
| 242 | LOC411631 | LOC725049 |  |  |
| 243 | LOC411746 | LOC725105 |  |  |
| 244 | LOC411748 | LOC725130 |  |  |
| 245 | LOC411797 | LOC725150 |  |  |
| 246 | LOC411805 | LOC725227 |  |  |
| 247 | LOC411806 | LOC725237 |  |  |
| 248 | LOC411811 | LOC725258 |  |  |
| 249 | LOC411843 | LOC725279 |  |  |
| 250 | LOC411886 | LOC725481 |  |  |
| 251 | LOC411904 | LOC725486 |  |  |
| 252 | LOC411960 | LOC725596 |  |  |
| 253 | LOC411989 | LOC725776 |  |  |
| 254 | LOC412025 | LOC725807 |  |  |
| 255 | LOC412069 | LOC725842 |  |  |
| 256 | LOC412109 | LOC725938 |  |  |
| 257 | LOC412164 | LOC726002 |  |  |
| 258 | LOC412176 | LOC726007 |  |  |
| 259 | LOC412192 | LOC726054 |  |  |
| 260 | LOC412234 | LOC726139 |  |  |
| 261 | LOC412235 | LOC726174 |  |  |
| 262 | LOC412289 | LOC726193 |  |  |
| 263 | LOC412291 | LOC726283 |  |  |
| 264 | LOC412343 | LOC726362 |  |  |
| 265 | LOC412355 | LOC726929 |  |  |
| 266 | LOC412396 | LOC726948 |  |  |
| 267 | LOC412398 | LOC727180 |  |  |
| 268 | LOC412399 | LOC727199 |  |  |
| 269 | LOC412423 | LOC727237 |  |  |
| 270 | LOC412452 | LOC727349 |  |  |
| 271 | LOC412503 | LOC727378 |  |  |
| 272 | LOC412504 | LOC727599 |  |  |
| 273 | LOC412510 | Nrx-1 |  |  |
| 274 | LOC412522 |  |  |  |
| 275 | LOC412531 |  |  |  |
| 276 | LOC412546 |  |  |  |
| 277 | LOC412594 |  |  |  |
| 278 | LOC412647 |  |  |  |
| 279 | LOC412661 |  |  |  |
| 280 | LOC412674 |  |  |  |
| 281 | LOC412714 |  |  |  |
| 282 | LOC412784 |  |  |  |
| 283 | LOC412818 |  |  |  |
| 284 | LOC412829 |  |  |  |
| 285 | LOC412830 |  |  |  |
| 286 | LOC412885 |  |  |  |
| 287 | LOC412890 |  |  |  |
| 288 | LOC412929 |  |  |  |
| 289 | LOC413024 |  |  |  |
| 290 | LOC413034 |  |  |  |
| 291 | LOC413080 |  |  |  |
| 292 | LOC413081 |  |  |  |
| 293 | LOC413126 |  |  |  |
| 294 | LOC413189 |  |  |  |
| 295 | LOC413190 |  |  |  |
| 296 | LOC413256 |  |  |  |
| 297 | LOC413382 |  |  |  |
| 298 | LOC413450 |  |  |  |
| 299 | LOC413467 |  |  |  |
| 300 | LOC413468 |  |  |  |
| 301 | LOC413490 |  |  |  |
| 302 | LOC413524 |  |  |  |
| 303 | LOC413578 |  |  |  |
| 304 | LOC413582 |  |  |  |
| 305 | LOC413590 |  |  |  |
| 306 | LOC413591 |  |  |  |
| 307 | LOC413616 |  |  |  |
| 308 | LOC413758 |  |  |  |
| 309 | LOC413759 |  |  |  |
| 310 | LOC413829 |  |  |  |
| 311 | LOC413907 |  |  |  |
| 312 | LOC413908 |  |  |  |
| 313 | LOC413926 |  |  |  |
| 314 | LOC413947 |  |  |  |
| 315 | LOC413994 |  |  |  |
| 316 | LOC414021 |  |  |  |
| 317 | LOC414022 |  |  |  |
| 318 | LOC550689 |  |  |  |
| 319 | LOC550692 |  |  |  |
| 320 | LOC550698 |  |  |  |
| 321 | LOC550735 |  |  |  |
| 322 | LOC550745 |  |  |  |
| 323 | LOC550780 |  |  |  |
| 324 | LOC550834 |  |  |  |
| 325 | LOC550915 |  |  |  |
| 326 | LOC550969 |  |  |  |
| 327 | LOC551020 |  |  |  |
| 328 | LOC551053 |  |  |  |
| 329 | LOC551079 |  |  |  |
| 330 | LOC551094 |  |  |  |
| 331 | LOC551097 |  |  |  |
| 332 | LOC551149 |  |  |  |
| 333 | LOC551170 |  |  |  |
| 334 | LOC551188 |  |  |  |
| 335 | LOC551201 |  |  |  |
| 336 | LOC551222 |  |  |  |
| 337 | LOC551240 |  |  |  |
| 338 | LOC551308 |  |  |  |
| 339 | LOC551356 |  |  |  |
| 340 | LOC551371 |  |  |  |
| 341 | LOC551376 |  |  |  |
| 342 | LOC551381 |  |  |  |
| 343 | LOC551398 |  |  |  |
| 344 | LOC551408 |  |  |  |
| 345 | LOC551445 |  |  |  |
| 346 | LOC551469 |  |  |  |
| 347 | LOC551497 |  |  |  |
| 348 | LOC551523 |  |  |  |
| 349 | LOC551524 |  |  |  |
| 350 | LOC551554 |  |  |  |
| 351 | LOC551557 |  |  |  |
| 352 | LOC551566 |  |  |  |
| 353 | LOC551571 |  |  |  |
| 354 | LOC551576 |  |  |  |
| 355 | LOC551588 |  |  |  |
| 356 | LOC551620 |  |  |  |
| 357 | LOC551644 |  |  |  |
| 358 | LOC551673 |  |  |  |
| 359 | LOC551677 |  |  |  |
| 360 | LOC551682 |  |  |  |
| 361 | LOC551696 |  |  |  |
| 362 | LOC551724 |  |  |  |
| 363 | LOC551746 |  |  |  |
| 364 | LOC551754 |  |  |  |
| 365 | LOC551769 |  |  |  |
| 366 | LOC551784 |  |  |  |
| 367 | LOC551790 |  |  |  |
| 368 | LOC551818 |  |  |  |
| 369 | LOC551824 |  |  |  |
| 370 | LOC551888 |  |  |  |
| 371 | LOC551905 |  |  |  |
| 372 | LOC551907 |  |  |  |
| 373 | LOC551936 |  |  |  |
| 374 | LOC551939 |  |  |  |
| 375 | LOC551957 |  |  |  |
| 376 | LOC552002 |  |  |  |
| 377 | LOC552025 |  |  |  |
| 378 | LOC552036 |  |  |  |
| 379 | LOC552063 |  |  |  |
| 380 | LOC552065 |  |  |  |
| 381 | LOC552089 |  |  |  |
| 382 | LOC552091 |  |  |  |
| 383 | LOC552106 |  |  |  |
| 384 | LOC552115 |  |  |  |
| 385 | LOC552125 |  |  |  |
| 386 | LOC552150 |  |  |  |
| 387 | LOC552172 |  |  |  |
| 388 | LOC552173 |  |  |  |
| 389 | LOC552198 |  |  |  |
| 390 | LOC552249 |  |  |  |
| 391 | LOC552273 |  |  |  |
| 392 | LOC552284 |  |  |  |
| 393 | LOC552289 |  |  |  |
| 394 | LOC552291 |  |  |  |
| 395 | LOC552293 |  |  |  |
| 396 | LOC552301 |  |  |  |
| 397 | LOC552315 |  |  |  |
| 398 | LOC552345 |  |  |  |
| 399 | LOC552372 |  |  |  |
| 400 | LOC552421 |  |  |  |
| 401 | LOC552435 |  |  |  |
| 402 | LOC552442 |  |  |  |
| 403 | LOC552467 |  |  |  |
| 404 | LOC552488 |  |  |  |
| 405 | LOC552490 |  |  |  |
| 406 | LOC552502 |  |  |  |
| 407 | LOC552549 |  |  |  |
| 408 | LOC552616 |  |  |  |
| 409 | LOC552642 |  |  |  |
| 410 | LOC552644 |  |  |  |
| 411 | LOC552654 |  |  |  |
| 412 | LOC552660 |  |  |  |
| 413 | LOC552662 |  |  |  |
| 414 | LOC552677 |  |  |  |
| 415 | LOC552678 |  |  |  |
| 416 | LOC552686 |  |  |  |
| 417 | LOC552709 |  |  |  |
| 418 | LOC724118 |  |  |  |
| 419 | LOC724131 |  |  |  |
| 420 | LOC724167 |  |  |  |
| 421 | LOC724193 |  |  |  |
| 422 | LOC724215 |  |  |  |
| 423 | LOC724269 |  |  |  |
| 424 | LOC724296 |  |  |  |
| 425 | LOC724329 |  |  |  |
| 426 | LOC724333 |  |  |  |
| 427 | LOC724380 |  |  |  |
| 428 | LOC724389 |  |  |  |
| 429 | LOC724417 |  |  |  |
| 430 | LOC724421 |  |  |  |
| 431 | LOC724450 |  |  |  |
| 432 | LOC724466 |  |  |  |
| 433 | LOC724467 |  |  |  |
| 434 | LOC724481 |  |  |  |
| 435 | LOC724513 |  |  |  |
| 436 | LOC724536 |  |  |  |
| 437 | LOC724538 |  |  |  |
| 438 | LOC724551 |  |  |  |
| 439 | LOC724560 |  |  |  |
| 440 | LOC724585 |  |  |  |
| 441 | LOC724596 |  |  |  |
| 442 | LOC724603 |  |  |  |
| 443 | LOC724634 |  |  |  |
| 444 | LOC724669 |  |  |  |
| 445 | LOC724673 |  |  |  |
| 446 | LOC724676 |  |  |  |
| 447 | LOC724681 |  |  |  |
| 448 | LOC724706 |  |  |  |
| 449 | LOC724757 |  |  |  |
| 450 | LOC724760 |  |  |  |
| 451 | LOC724773 |  |  |  |
| 452 | LOC724794 |  |  |  |
| 453 | LOC724826 |  |  |  |
| 454 | LOC724843 |  |  |  |
| 455 | LOC724869 |  |  |  |
| 456 | LOC724870 |  |  |  |
| 457 | LOC724875 |  |  |  |
| 458 | LOC724886 |  |  |  |
| 459 | LOC724893 |  |  |  |
| 460 | LOC724903 |  |  |  |
| 461 | LOC724912 |  |  |  |
| 462 | LOC724999 |  |  |  |
| 463 | LOC725021 |  |  |  |
| 464 | LOC725024 |  |  |  |
| 465 | LOC725031 |  |  |  |
| 466 | LOC725039 |  |  |  |
| 467 | LOC725040 |  |  |  |
| 468 | LOC725049 |  |  |  |
| 469 | LOC725097 |  |  |  |
| 470 | LOC725105 |  |  |  |
| 471 | LOC725130 |  |  |  |
| 472 | LOC725150 |  |  |  |
| 473 | LOC725227 |  |  |  |
| 474 | LOC725237 |  |  |  |
| 475 | LOC725258 |  |  |  |
| 476 | LOC725268 |  |  |  |
| 477 | LOC725279 |  |  |  |
| 478 | LOC725316 |  |  |  |
| 479 | LOC725481 |  |  |  |
| 480 | LOC725486 |  |  |  |
| 481 | LOC725524 |  |  |  |
| 482 | LOC725596 |  |  |  |
| 483 | LOC725599 |  |  |  |
| 484 | LOC725673 |  |  |  |
| 485 | LOC725770 |  |  |  |
| 486 | LOC725776 |  |  |  |
| 487 | LOC725792 |  |  |  |
| 488 | LOC725807 |  |  |  |
| 489 | LOC725842 |  |  |  |
| 490 | LOC725850 |  |  |  |
| 491 | LOC725890 |  |  |  |
| 492 | LOC725894 |  |  |  |
| 493 | LOC725905 |  |  |  |
| 494 | LOC725938 |  |  |  |
| 495 | LOC726002 |  |  |  |
| 496 | LOC726007 |  |  |  |
| 497 | LOC726054 |  |  |  |
| 498 | LOC726056 |  |  |  |
| 499 | LOC726068 |  |  |  |
| 500 | LOC726108 |  |  |  |
| 501 | LOC726139 |  |  |  |
| 502 | LOC726167 |  |  |  |
| 503 | LOC726174 |  |  |  |
| 504 | LOC726193 |  |  |  |
| 505 | LOC726220 |  |  |  |
| 506 | LOC726272 |  |  |  |
| 507 | LOC726277 |  |  |  |
| 508 | LOC726283 |  |  |  |
| 509 | LOC726320 |  |  |  |
| 510 | LOC726326 |  |  |  |
| 511 | LOC726362 |  |  |  |
| 512 | LOC726368 |  |  |  |
| 513 | LOC726389 |  |  |  |
| 514 | LOC726405 |  |  |  |
| 515 | LOC726415 |  |  |  |
| 516 | LOC726431 |  |  |  |
| 517 | LOC726444 |  |  |  |
| 518 | LOC726488 |  |  |  |
| 519 | LOC726496 |  |  |  |
| 520 | LOC726690 |  |  |  |
| 521 | LOC726740 |  |  |  |
| 522 | LOC726778 |  |  |  |
| 523 | LOC726811 |  |  |  |
| 524 | LOC726822 |  |  |  |
| 525 | LOC726929 |  |  |  |
| 526 | LOC726948 |  |  |  |
| 527 | LOC727000 |  |  |  |
| 528 | LOC727049 |  |  |  |
| 529 | LOC727106 |  |  |  |
| 530 | LOC727119 |  |  |  |
| 531 | LOC727122 |  |  |  |
| 532 | LOC727153 |  |  |  |
| 533 | LOC727157 |  |  |  |
| 534 | LOC727180 |  |  |  |
| 535 | LOC727199 |  |  |  |
| 536 | LOC727237 |  |  |  |
| 537 | LOC727243 |  |  |  |
| 538 | LOC727290 |  |  |  |
| 539 | LOC727299 |  |  |  |
| 540 | LOC727349 |  |  |  |
| 541 | LOC727378 |  |  |  |
| 542 | LOC727436 |  |  |  |
| 543 | LOC727522 |  |  |  |
| 544 | LOC727598 |  |  |  |
| 545 | LOC727599 |  |  |  |
| 546 | LOC727634 |  |  |  |
| 547 | NLG-3 |  |  |  |
| 548 | Nrx-1 |  |  |  |
| 549 | Obp3 |  |  |  |
| 550 | Obp4 |  |  |  |

| **Table S10. Top enriched GO terms among candidate introgressed regions (Biological Process, Molecular Function, Cellular Component)** | | | | | |
| --- | --- | --- | --- | --- | --- |
| **（Biological Process, BP）** | | | | | |
| **ID** | **Description** | **GeneRatio** | **pvalue** | **geneID** | **Count** |
| GO:0007268 | chemical synaptic transmission | 28/427 | 5.23E-06 | slo/Ih/Dys/mfr/Shab/Ank2/HDAC6/Ten-m/stac/Nmdar2/X11Lbeta/X11L/Arl2/Dcp-1/Thor/Syx13/Arl1/5-HT7/CG6927/Exo70/Pka-R1/Arl8/ohgt/stmA/dnc/na/Hsc70-4/Sol1 | 28 |
| GO:0098916 | anterograde trans-synaptic signaling | 28/427 | 5.23E-06 | slo/Ih/Dys/mfr/Shab/Ank2/HDAC6/Ten-m/stac/Nmdar2/X11Lbeta/X11L/Arl2/Dcp-1/Thor/Syx13/Arl1/5-HT7/CG6927/Exo70/Pka-R1/Arl8/ohgt/stmA/dnc/na/Hsc70-4/Sol1 | 28 |
| GO:0099537 | trans-synaptic signaling | 28/427 | 6.99E-06 | slo/Ih/Dys/mfr/Shab/Ank2/HDAC6/Ten-m/stac/Nmdar2/X11Lbeta/X11L/Arl2/Dcp-1/Thor/Syx13/Arl1/5-HT7/CG6927/Exo70/Pka-R1/Arl8/ohgt/stmA/dnc/na/Hsc70-4/Sol1 | 28 |
| GO:0099536 | synaptic signaling | 28/427 | 1.10E-05 | slo/Ih/Dys/mfr/Shab/Ank2/HDAC6/Ten-m/stac/Nmdar2/X11Lbeta/X11L/Arl2/Dcp-1/Thor/Syx13/Arl1/5-HT7/CG6927/Exo70/Pka-R1/Arl8/ohgt/stmA/dnc/na/Hsc70-4/Sol1 | 28 |
| GO:0007269 | neurotransmitter secretion | 13/427 | 2.69E-05 | Ih/Dys/mfr/HDAC6/stac/Arl2/Syx13/Arl1/Exo70/Arl8/stmA/Hsc70-4/Sol1 | 13 |
| GO:0099643 | signal release from synapse | 13/427 | 2.69E-05 | Ih/Dys/mfr/HDAC6/stac/Arl2/Syx13/Arl1/Exo70/Arl8/stmA/Hsc70-4/Sol1 | 13 |
| GO:0007365 | periodic partitioning | 9/427 | 2.70E-05 | hh/ci/sgg/hop/smo/frc/Dcr-1/SoxN/kn | 9 |
| GO:0051240 | positive regulation of multicellular organismal process | 20/427 | 3.26E-05 | Ih/hh/dar1/Dap160/Shab/Ank2/Tmc/RhoGAP68F/Rac1/mbt/lva/Nf-YB/Sting/Pka-R1/RIOK2/RIOK1/Rheb/Nca/ari-1/mnb | 20 |
| GO:0007367 | segment polarity determination | 8/427 | 3.84E-05 | hh/ci/sgg/smo/frc/Dcr-1/SoxN/kn | 8 |
| **（Molecular Function, MF）** | | | | | |
| **ID** | **Description** | **GeneRatio** | **pvalue** | **geneID** | **Count** |
| GO:0019899 | enzyme binding | 33/392 | 3.47E-06 | nsl1/CG42673/ci/CG10508/Msp300/RhoGAP68F/Rac1/cos/Sumo/ben/mbt/S6kII/smo/POSH/Drice/trbl/abo/Rtel1/Gapvd1/Brms1/CG15160/Cul4/PPP1R15/mats/CG6607/barr/CG30496/ago/pbl/Fancd2/ari-1/CycY/Pcif1 | 33 |
| GO:0140657 | ATP-dependent activity | 28/392 | 7.87E-05 | jar/Taf1/Klp98A/Klp67A/Mcm3/spn-B/Top2/Mlh1/cos/Rtel1/CCT2/Myo10A/Vps4/mtDNA-helicase/SoYb/CG17904/sick/APP-BP1/ais/Fancm/SMC1/CG6178/snu/eIF4A/Prp16/AcCoAS/Hsc70-4/dpa | 28 |
| GO:0019901 | protein kinase binding | 10/392 | 4.09E-04 | ci/Msp300/Rac1/cos/S6kII/smo/POSH/trbl/mats/CycY | 10 |
| GO:0019900 | kinase binding | 11/392 | 4.79E-04 | ci/CG10508/Msp300/Rac1/cos/S6kII/smo/POSH/trbl/mats/CycY | 11 |
| GO:1901981 | phosphatidylinositol phosphate binding | 9/392 | 5.75E-04 | CG33966/Klp98A/smo/Exo70/Sec3/pinta/CG10237/pbl/CG2663 | 9 |
| GO:0030234 | enzyme regulator activity | 28/392 | 6.26E-04 | CG17883/Dap160/Nup358/RhoGAP68F/ArfGAP3/Socs16D/trbl/Gapvd1/Zir/Sirup/Trs23/CG30440/CG8243/CG17739/PPP1R15/Pka-R1/CG12241/mats/Spn27A/Socs36E/barr/CG30496/Oda/pbl/RSG7/CycY/RhoGDI/mnb | 28 |
| GO:0042802 | identical protein binding | 13/392 | 8.38E-04 | Atf-2/ci/Oscillin/Ten-m/smo/Orco/trbl/Roe1/GlyP/IPIP/Ork1/Liprin-beta/Scm | 13 |
| GO:0035091 | phosphatidylinositol binding | 10/392 | 1.23E-03 | CG33966/Klp98A/smo/hob/Exo70/Sec3/pinta/CG10237/pbl/CG2663 | 10 |
| GO:0004386 | helicase activity | 9/392 | 1.31E-03 | Mcm3/Rtel1/mtDNA-helicase/SoYb/ais/Fancm/eIF4A/Prp16/dpa | 9 |
| **(****Cellular Component, CC）** | | | | | |
| **ID** | **Description** | **GeneRatio** | **pvalue** | **geneID** | **Count** |
| GO:0045202 | synapse | 26/406 | 3.53E-05 | slo/Dys/mfr/Dap160/Ank2/CG42673/sgg/Ten-m/stac/Nmdar2/X11Lbeta/X11L/Thor/S6kII/S6k/RabX1/CG6927/RabX5/Exo70/Arl8/stmA/na/Liprin-beta/Septin4/Sol1/mnb | 26 |
| GO:0031461 | cullin-RING ubiquitin ligase complex | 13/406 | 5.34E-05 | CG7568/CG42784/CG12084/abo/APC4/Cul4/dmpd/Roc2/ohgt/CG30496/CG9945/ago/CG32085 | 13 |
| GO:0000151 | ubiquitin ligase complex | 15/406 | 2.42E-04 | CG7568/CG42784/CG12084/abo/APC4/Ube4A/Cul4/dmpd/Roc2/ohgt/CG30496/CG9945/ago/CG32085/ari-1 | 15 |
| GO:0036477 | somatodendritic compartment | 21/406 | 3.19E-04 | slo/CG4537/Tmc/mas/Ten-m/X11Lbeta/X11L/Dcp-1/S6kII/smo/Or30a/Orco/hdc/5-HT7/Or2a/Myo10A/TrpgA.m.ma/br/na/Tao/Or82a | 21 |
| GO:0031410 | cytoplasmic vesicle | 28/406 | 6.40E-04 | jar/CG33966/hh/mfr/Dap160/Klp98A/RhoGAP68F/stac/Rme-8/Rac1/Pi3K59F/TSG101/RabX1/Gapvd1/CG8134/SWIP/Vps4/Trs23/CG5946/AP-2sigma/CG6607/snu/IPIP/CG10237/Mvb12/car/CG2663/Septin4 | 28 |
| GO:0097708 | intracellular vesicle | 28/406 | 6.40E-04 | jar/CG33966/hh/mfr/Dap160/Klp98A/RhoGAP68F/stac/Rme-8/Rac1/Pi3K59F/TSG101/RabX1/Gapvd1/CG8134/SWIP/Vps4/Trs23/CG5946/AP-2sigma/CG6607/snu/IPIP/CG10237/Mvb12/car/CG2663/Septin4 | 28 |
| GO:0031982 | vesicle | 30/406 | 6.87E-04 | jar/CG33966/hh/mfr/Dap160/Klp98A/RhoGAP68F/stac/Rme-8/Rac1/Pi3K59F/TSG101/RabX1/Myo10A/Gapvd1/CG8134/SWIP/Vps4/Trs23/RabX5/CG5946/AP-2sigma/CG6607/snu/IPIP/CG10237/Mvb12/car/CG2663/Septin4 | 30 |
| GO:0080008 | Cul4-RING E3 ubiquitin ligase complex | 4/406 | 8.32E-04 | CG42784/Cul4/ohgt/CG9945 | 4 |
| GO:0030054 | cell junction | 29/406 | 9.12E-04 | slo/Dys/mfr/Dap160/Ank2/CG42673/sgg/Ten-m/ArfGAP3/stac/Nmdar2/X11Lbeta/X11L/Thor/mbt/S6kII/S6k/RabX1/CG6927/parvin/RabX5/Exo70/Arl8/stmA/na/Liprin-beta/Septin4/Sol1/mnb | 29 |

| **Table S11 Summary gene annotation of candidate genes under putative introgression** | | | |
| --- | --- | --- | --- |
| **Gene identified** | **DESCRIPTION** | **Association** | **Reference** |
| LOC551696 （Also known as GB41973）TXNDC9 | Trx-1，thioredoxin domain-containing protein 9 | Physiology and Pathology,cell differentiation | <https://www.frontiersin.org/articles/10.3389/fmolb.2022.813637/full> |
| LOC727378(Also known as GB10906) | peptidyl-prolyl cis-trans isomerase-like 1 | a key signalling mechanism in diverse physiological and pathological processes | / |
| LOC726948 | CRnnnn，suppressor of lurcher protein 1 | synaptic plasticity, learning, and memory abilities | / |
| LOC726007 | polycomb protein Scm | This gene encodes a member of the Polycomb group proteins. These proteins form the Polycomb repressive complexes which are involved in transcriptional repression. The encoded protein binds histone peptides that are monomethylated at lysine residues and may be involved in regulating homeotic gene expression during development. [provided by RefSeq, Jun 2010] | / |
| LOC724673 | / | olfactory sensory (olfactory sensory neurons detect odorants and direct sensory axons toward precise target locations in the brain.) | |
| LOC724269 | RpL3，connectin | ovary state | [10.1007/s13592-020-00760-7](http://dx.doi.org/10.1007/s13592-020-00760-7) |
| LOC552291 | DNA replication licensing factor MCM4 | modulate the pattern of replication in response to environmental conditions through the replicative helicase. | doi/10.1073/pnas.1404063111 |
| LOC552273 | Diptericin，phosphoribosyl pyrophosphate synthase-associated protein 2， | spermatogenic cell apoptosis and is correlated with hypospermatogenesis | 10.4103/aja.aja_122_19 |
| LOC551754 | phosphorylated CTD-interacting factor 1，Syntaxin1A | mRNA synthesis | / |
| LOC551554 | ras-related protein Rac1 | cell proliferation | (McCormick, 1995) |
| LOC551308 | probable serine/threonine-protein kinase ndrD | cell proliferation | / |
| LOC550698 | cyclin-Y | cell cycle and transcription | / |
| LOC413616 | tyrosine-protein kinase transmembrane receptor Ror | RORs function in developmental processes including skeletal and neuronal development, cell movement and cell polarity. | / |

| **Table S12: Kyoto Encyclopedia of Genes and Genomes (KEGG) pathway enrichment analysis of putatively introgressed genes** | | | | | | | | |
| --- | --- | --- | --- | --- | --- | --- | --- | --- |
| **category** | **subcategory** | **ID** | **Description** | **GeneRatio** | **BgRatio** | **pvalue** | **geneID** | **Count** |
| Metabolism | Carbohydrate metabolism | dme00500 | Starch and sucrose metabolism - Drosophila melanogaster (fruit fly) | 6/158 | 32/3533 | 0.002504437 | 39065/37419/34597/33386/34598/45368 | 6 |
| Metabolism | Nucleotide metabolism | dme00240 | Pyrimidine metabolism - Drosophila melanogaster (fruit fly) | 5/158 | 38/3533 | 0.025536742 | 34392/32396/33495/318908/31858 | 5 |
| Environmental Information Processing | Signal transduction | dme04341 | Hedgehog signaling pathway - fly - Drosophila melanogaster (fruit fly) | 5/158 | 40/3533 | 0.031178302 | 42737/43767/31248/35653/33196 | 5 |
